# Carbon Fluxes of Contrasting Degraded Peatland Pilot Sites During Early-stage Restoration: Ex-milled Bare Peat and Grazed Grassland Conversion

**DOI:** 10.1007/s00267-026-02445-w

**Published:** 2026-04-11

**Authors:** Anna T. Keightley, Christopher D. Field, Michael Longden, Simon J. M. Caporn

**Affiliations:** 1 Manchester Metropolitan University, School of Engineering and Environment, Manchester, UK; 2 Lancashire Wildlife Trust, Preston, UK

**Keywords:** degraded peatlands, Sphagnum, Eriophorum, water table, greenhouse gases

## Abstract

The recovery of degraded peatlands can make significant contributions to reducing greenhouse gas emissions and climate warming. This study examines restoration techniques on shallow ex-milled peatland and intensively grazed pasture on deeper peat, both subject to prior drainage. Carbon greenhouse gases (GHGs) were monitored for 3 years following restoration treatment. After drainage-blocking measures, the ex-milled peatland was ‘companion planted’ with *Eriophorum* species and *Sphagnum*. The carbon balance was highly dependent on plant age and condition, with a high CO_2_ equivalent (CO_2_e) uptake when plants were vigorously growing (year 1: −22.4 ± 32.9 t CO_2_e ha^−1^ yr^−1^), and high emission when plants were mature and in various stages of senescence (year 2: 26.1 ± 26.4 and year 3: 16.4 ± 9.7 t CO_2_e ha^−1^ yr^−1^). Bare peat controls had a mean emission of 6.21 ± 1.68 t CO_2_e ha^−1^ yr^−1^ over the study period. At the other site, the grazed pasture was stripped, the bare surface planted with *Sphagnum* plugs, and irrigation was intensively managed via bunding, ditches, and automatic water pumping. Carbon GHG emissions were significantly reduced on this ‘carbon farm’ (2.77 ± 0.95 t CO_2_e ha^−1^ yr^−1^) compared to a neighbouring drained, grazed pasture control (31.7 ± 10.3 t CO_2_e ha^−1^ yr^−1^) over the study period (mean ± SD throughout). It appears clear that the cyclical nature of *Eriophorum* plant growth may only deliver carbon benefits on shallow peat over the long term if groundwater levels can be adequately supported and if climatic conditions are favourable. Conversion of grazed pasture to wetter farming crops, such as *Sphagnum*, can potentially deliver immediate carbon benefits, although, in this pilot, any potential loss of CO_2_e due to degraded topsoil removal, creation of bunds and irrigation ditches was not accounted for.

## Introduction

Undisturbed peatlands sequester and retain carbon under conditions of high moisture and acidity that decrease the microbial activities involved in decomposition of dead plant material, thereby reducing CO_2_ emissions (Clymo 1984; Limpens et al. 2008; Andersen et al. 2013; Jayasekara et al. 2025). *Sphagnum* mosses (also termed ‘peat mosses’) are key to this process in northern hemisphere bogs (Rochefort 2000), growing continually from the tip of the plants to sequester carbon, releasing acids and promoting water retention, with the layers below compressing over time in anaerobic conditions to create high carbon-content peat. However, peatlands across the world have been increasingly degraded through drainage and conversion to various uses, mainly agriculture, forestry, and extraction for horticulture, causing oxidation and releasing stored carbon into the atmosphere as CO_2_, diminishing peatland stocks and destroying peatland biodiversity (UNEP 2022). Degraded peatlands may contribute 5–10% of all anthropogenic CO_2_ emissions, contributing to global climate heating (Loisel and Gallego-Sala 2022).

Preventing further damage to degraded peatlands, primarily through re-wetting, is therefore important to slow climate warming by protecting current carbon stocks (Leifeld and Menichetti 2018; Günther et al. 2020). Moreover, there are also economic and societal costs (Glenk and Martin-Ortega 2018; Glenk et al. 2021) to doing nothing and allowing degraded peatlands to continue emitting greenhouse gases (GHGs). Conversion of agriculture on peatlands to wetter forms of farming (paludiculture) could be a key component (Wichtmann et al. 2016) but efforts are compromised by food production needs, the lack of funding for development of wetter farming techniques (Freeman et al. 2022), and the challenges of securing potential markets for paludiculture products (Ross 2025). The benefits of growing *Sphagnum* moss for ecosystem services, both for restoration and as a paludiculture crop, have been demonstrated (Temmink et al. 2024), but funding streams to support biodiversity gains and carbon savings are also underdeveloped (Liu et al. 2023). It is therefore difficult to compete with demands for higher income crops on drained land (Wichmann et al. 2020). There are, however, promising new initiatives to encourage the development of paludiculture crops (NIAB 2024) and the conversion of ‘carbon into cash’ can be supported by more evidence of methods by which peatlands can rewet and reduce emissions, giving confidence to financial bodies and governments to support schemes. Emissions factors for a range of peatland uses have been developed (e.g., IPCC 2014; Evans et al. 2017), and their scope and precision are continually improving, enabling validation of decisions on large-scale land use change towards wetter scenarios.

The Interreg North West Europe-funded Care-Peat Project, active from 2019 to 2023 (Care-Peat 2023), aimed to work towards restoring the carbon storage capacity of peatlands, using a range of innovative techniques in diverse scenarios, across five countries in NW Europe (Belgium, France, Ireland, Netherlands, UK). Two peatland sites in North West England owned by Lancashire Wildlife Trust (LWT) served as the UK pilots: an ex-milled area on Little Woolden Moss (now within the Risley, Holcroft and Chat Moss National Nature Reserve) and a grazed pasture adjacent to the conservation peatland of Winmarleigh Moss Site of Special Scientific Interest (SSSI). Techniques based on established restoration practices of re-wetting and re-vegetating were employed on both pilots. The objectives of this research were to understand the effect on the rates of net CO_2_ and CH_4_ exchange of (i) ‘companion planting’ *Sphagnum* moss and *Eriophorum* spp. (Cottongrasses) on the ex-milled peat site, with minimal interventions post-planting and (ii) ‘Carbon Farming’: returning grazed grassland to a peatbog following turf-stripping, rewetting and *Sphagnum* moss planting, with highly engineered and intensive water level management.

The rationale for the companion planting was that *Eriophorum* species with different growth habits would rapidly colonize the bare peat, sequestering CO_2_ through photosynthesis, and also support *Sphagnum* establishment by providing environmental protection (Heijmans et al. 2001; Pouliot et al. 2011) based on work on an adjacent site by Keightley et al. (2023). This arrangement could potentially sequester more CO_2_e through mutual plant support (Kivimäki et al. 2008) and mitigate some methane emissions from *Eriophorum* plants, as the methane-filtering properties of *Sphagnum* (through methane-oxidizing bacteria) are well-documented (Parmentier et al. 2011; Kox et al. 2021; Daun et al. 2023).

The rationale for the Carbon Farm set-up of turf-stripping, bunding, irrigating and densely planting with fast-growing *Sphagnum* was to provide favourable conditions for the *Sphagnum* to establish quickly and thrive (Käärmelahti et al. 2024), therefore optimising carbon sequestion potential. The time-constraints of a 3-year project precluded an alternative, long-term (and perhaps preferable) approach of cessation of drainage coupled with nutrient-absorbing planting (e.g., *Typha latifolia*, Vroom et al. 2018) as ground preparation to both re-wet the peat and remove previous agricultural inputs prior to *Sphagnum*-planting. Rewetting only, without topsoil removal, of agricultural peatlands may be a reasonable option in the short term as it immediately reduces CO_2_ losses related to drainage, but CH_4_ emission may rise substantially (Huth et al. 2022) and mobilisation of agricultural nutrients can lead to eutrophication, high dissolved organic carbon losses, and poor suitability for *Sphagnum* establishment and growth (Harpenslager et al. 2015; Zak et al. 2018). Preferably, removed topsoil can be utilized elsewhere, for example, in levelling agricultural subsidence (Harpenslager et al. 2015), infilling ditches, or creation of peat bunds, for an overall carbon benefit.

It was anticipated that the approach used to create the Carbon Farm would offset emissions through rewetting, since near-natural bogs are generally carbon neutral (Evans et al. 2017), eliminate probable emissions from surface peats subject to historic agricultural inputs (Quadra et al. 2023), and potentially actively store carbon within a short time-scale. ‘Carbon farming’ is a relatively novel concept, whereby farmers may potentially be paid for ensuring a quantity of carbon is ‘farmed’ from the atmosphere and retained in the soil rather than removed through traditional farming practices and crops (Kennedy et al. 2023b).

The *Sphagnum* in both pilots was micropropagated and supplied by BeadaMoss® (East Leake, Leicestershire, UK) as described by Keightley et al. (2023, 2024). Monitoring of GHGs, site vegetation, and environmental variables continued after the end of the Care-Peat project until January 2024 so that three full years of data were collected to demonstrate the efficacy of the methods employed in terms of GHG emissions and planting success.

## Methods

### Site Descriptions

#### Companion Planting Pilot (Little Woolden Moss)

**Fig. 1:**
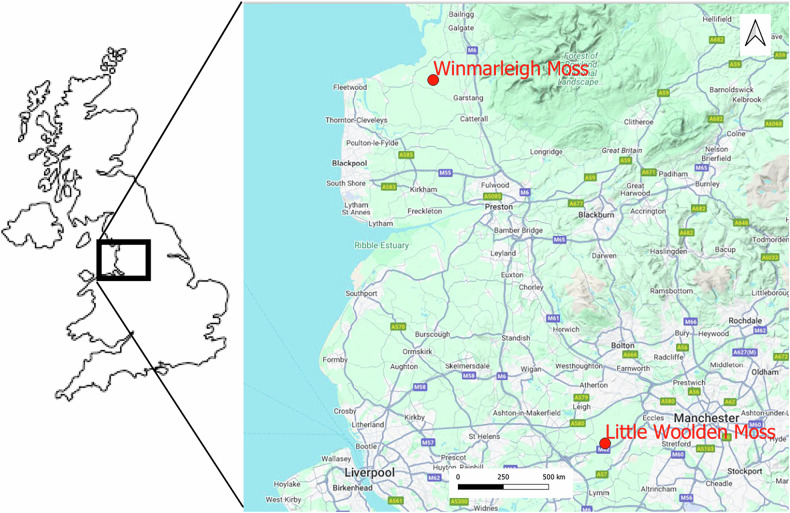
Location of pilot sites, Northern England. Winmarleigh Moss, in a coastal, rural location; Little Woolden Moss close to the large conurbation of Manchester (map created using QGIS)

**Fig. 2:**
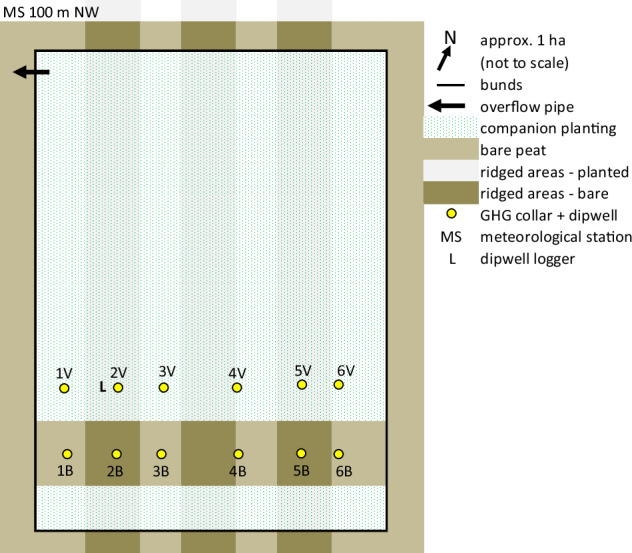
Diagram (not to scale) of Companion Planting (Little Woolden Moss) pilot layout. Plots 1 & 3 on infilled ditches, plots 2 & 5 on old peat extraction beds, plots 4 and 6 on intermediate areas; V = vegetated; B = bare. Similar version previously printed in Kennedy et al. (2023a)

Little Woolden Moss (LWM) is a 107 ha ex-peat-milled lowland bog in Lancashire, North West England (53°26'47.1“N, 2°27'57.8“W) (Fig. 1), owned by LWT since 2012, and under restoration measures of ditch-infilling, levelling, peat-bunding and re-introduction of typical bog vegetation (Osborne et al. 2021). Industrial peat harvesting ceased in two phases: in 2012 on approximately 60% of the site, allowing restoration in that area; and in December 2017 on the remainder of the site, after an existing peat extraction tenure ended, allowing restoration thereafter. The 1 ha pilot was within the 2^nd^ phase area, enclosed with peat bunding (Fig. 2: diagram of the pilot site set-up).

Coarse bare peat (underlaid with wood, sand and clay) to a mean depth of 1.17 ± 0.19 m remained on the pilot site, with a slightly sloping topography and a legacy of ridges (milled beds) and depressions (infilled drainage ditches). Restoration used methods of ‘companion planting’ in which groups of *Eriophorum angustifolium* and *E. vaginatum* plug plants (produced by BeadaMoss®) at a ratio of 3:1 were planted randomly in March 2020, with groups spaced approximately 1 m apart. Subsequently, three plugs of BeadaMoss® 5-species mix *Sphagnum* (BeadaHumok™) comprised of *S. medium*, *S. palustre*, *S. papillosum*, *S. rubellum* and *S. subnitens* were planted within each *Eriophorum spp*. group in September 2020, but were immediately pulled up by corvid birds and little survived. A repeat planting of *Sphagnum* was anticipated once a complete sward of *Eriophorum* spp. had established, but was not implemented. An area was left deliberately bare as a Control.

The pilot site was subject to flooding, particularly in the north-west corner (not near GHG monitoring plots), during the winter and spring months, and much of the above-ground plant material was lost in that area due to wave action. *E. vaginatum* plants were a little more resilient than *E. angustifolium*, having a tighter growth habit. *E. angustifolium* plants showed subsequent signs of regrowth in some areas. An overflow pipe was re-sited to reduce flood water more effectively (full pilot details are given by Kennedy et al. 2023a).

#### Carbon Farm (Winmarleigh Moss)

The Winmarleigh Moss pilot (‘Carbon Farm’) is in Lancashire, North West England (53°55'32.8“N, 2°50'58.0“W) (Fig. 1), on a former lowland bog converted to agricultural land (grazed pasture) in the 1970s and acquired by LWT in 2019. LWT also owns the adjacent 89-hectare Winmarleigh and Cockerham Moss Site of Special Scientific Interest (SSSI), a lowland raised bog.

**Fig. 3:**
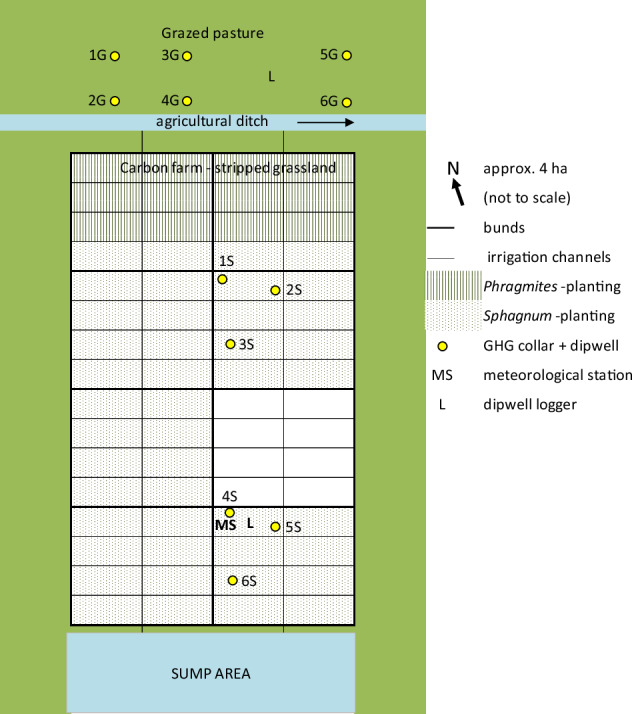
Diagram (not to scale) of Carbon Farm (Winmarleigh Moss) pilot layout. G = Grazed Pasture; S = Carbon Farm (Sphagnum). Plots 1S & 4S near bunding, furthest from water sources; plots 2S & 5S close to irrigation ditches; plots 3S & 6S on intermediate areas; Similar version previously printed in Kennedy et al. (2023b)

There was 1.5 to 1.7 m of peat remaining in the area, humified at the surface with a transition from *Sphagnum* to fen peat, underlaid with glacial clay (occasional woody deposits). The area was drained and grazed prior to study, leaving oxidized surface peat containing high levels of farming nutrients (nitrate = 156.7 ± 76.8 mg^−1^ kg^−1^ within the top 10 cm of peat). The upper 10 cm surface of the 4-hectare study site intended for the Carbon Farm (just under 2 hectares = 704 m^3^ carbon removed as the peat carbon content was 38.7%) was stripped in May 2020 to remove these effects, along with agricultural plant roots and seeds, then laser-levelled and peat-bunded into 8 cells of 50 × 50 m divided by water channels to irrigate the cells. The channels were fed from a sump, automatically pumped, controlled, and solar-powered, with the system targeting a water table of 10 cm below the surface, designed to minimize CO_2_ emissions but avoid elevated methane emissions associated with flooded peat (Evans et al. 2021). The adjacent grazed pasture acted as a Control area. (Fig. 3: diagram of the pilot site set-up).

Six of the cells were planted in September 2020 with 150,000 BeadaMoss® 5-species mix *Sphagnum* (BeadaHumok™) plugs plants (plugs) spaced 20 cm apart (i.e., 25 per m^2^), comprised of *S. medium*, *S. palustre*, *S. papillosum*, *S. rubellum* and *S. subnitens*. The *Sphagnum* was protected during establishment by a thin layer of straw, which also aided moisture retention (Fig. 18 [Appendix]); full pilot details are given by Kennedy et al. 2023b)

### Peat core measurements

Three peat cores were extracted from each treatment (‘Restoration’ and ‘Control’) area of each site, using a Russian corer. The fragile or friable surface peats were extracted using a sharp knife to prevent compression before extracting the deeper cores. The samples were divided into sections, bagged and refrigerated until analysis. The sections down the core were in 5 cm increments from 0-30 cm, then 30–40 cm, 40–50 cm, 50–70 cm, 70–100 cm, and finally in 25 cm increments until the mineral layer was reached.

Fresh samples were weighed, oven-dried (60 °C to a consistent mass for 48 to 72 hours), and dry weighed. The bulk density was calculated (Eq. 1) then samples were incinerated at 550 °C for 3 hours to obtain the loss-on-ignition (LOI) weight of organic material. The Carbon content of each section was calculated using an equation developed by Bojko and Kabala (2014) for organic soils (Eq. 2). The carbon stock in t ha^−1^ for each section was calculated (Eq. 3) and the section carbon stocks were combined for each core to give a total value for each area. Where there was an overall CGHG emission, the annual amount of carbon lost could then be calculated (Eq. 4).

1 $${\rm{Dry\; weight}}({\rm{g}})/{\rm{sample\; volume}}({{\rm{cm}}}^{3})({\rm{g}}{{\rm{cm}}}^{3})$$

2 $$(0.522{\rm{x}} \% \mathrm{LOI})-0.553\,( \% )$$

3 $${\rm{Dry\; Weight\; bulk\; density}}({\rm{g}}{{\rm{cm}}}^{3}){\rm{x\; Carbon\; content}}( \% ){\rm{x\; sample\; length}}({\rm{cm}})\,({{\rm{t\; ha}}}^{-1})$$

4 $$\mathrm{Annual\; CGHG\; balance}({\rm{t}}{\mathrm{CO}2\mathrm{e\; ha}}^{-1}{\mathrm{yr}}^{-1}){\rm{x}}12\,/\,44\,({\mathrm{t\; C\; ha}}^{-1}\,{\mathrm{yr}}^{-1})$$

### Measurements of GHG flux, water table, and environmental variables

Carbon Greenhouse Gas (CGHG: CO_2_ and CH_4_) monitoring collars (30 cm internal diameter) were inserted (5 cm below ground and 5 cm above) with accompanying dipwells (within 20 cm of each collar) in areas with a likely range of moisture levels: legacy ridges and depressions on LWM and at varying distances from irrigation ditches on the Carbon Farm, with 6 collars in each Restoration and Control area (see Figs. 2 & 3). Sections of boardwalk were installed next to each CGHG monitoring point to prevent disturbance of the peat during measurements.

On the LWM pilot, collars were monitored for 12 months until vegetation became too dense (due to *E. vaginatum* expansion) to be representative of the pilot, and collars were becoming distorted. Collars were then relocated to adjacent stands of *E. angustifolium* that had naturally colonized during the first year and monitoring resumed after a 6-month gap. This gave an ‘establishment’ year from June 2020 (year 1) and a ‘post-establishment/mature’ year (year 2) from December 2021. Thereafter, clumps of *Sphagnum* material were taken from the pilot site, as a repeat application of *Sphagnum* across the pilot site was anticipated (although not implemented), and added to three of the collars (as described below) and monitoring continued for a further year (year 3) from February 2023. On the Carbon Farm pilot, measurements were made over a three-year period from December 2020 to January 2024, which was continuous apart from a 2-month gap from December 2022 to January 2023.

**Fig. 4:**
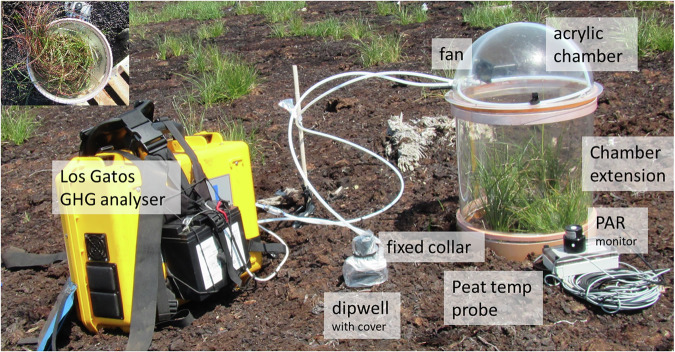
Typical GHG monitoring set-up, here at the Companion Planting (Little Woolden Moss) pilot site in the early stages of restoration (boardwalk not shown; inset: another collar with chamber extension and adjacent boardwalk). Figure previously printed in Kennedy et al. (2023a, b)

CGHG fluxes were measured monthly (Table S1) for three full years using a Los Gatos Ultraportable Analyzer (LGR) connected to a clear (acrylic) hemi-spherical chamber (0.0141 m^3^) placed over collars in the peat to enclose samples of either Restoration or Control scenarios (Fig. 4). An extruded acrylic extension tube (0.0119 m^3^) accommodated taller vegetation. A blackout cloth over the chamber simulated night-time (for Ecosystem Respiration: RECO and Methane: FCH_4_, measured concurrently), a mesh cloth (which reduced light by 36.3 ± 14.5%) simulated cloud cover and no cloth used for full daylight (for Net Ecosystem Exchange: NEE in semi- and full-light) in a range of light conditions (i.e., throughout the year), from which Gross Primary Productivity (GPP) could be calculated (NEE = RECO + GPP) for each measurement in the light.

Measurements were made, in this order (dark, semi-light, full-light), on each collar and monitoring occasion (i.e., 36 measurements per pilot site) for 2 minutes (once the measurement trajectory was obvious and stable), and the chamber was removed between measurements to flush gases until ambient conditions were achieved. Dark measurements were monitored first to reduce any effects of chamber heating. Peat temperature, photosynthetically active radiation (PAR), water table level below the surface (WTL) via dipwells, and CGHGs were measured concurrently at each monitoring collar. Peat temperature (5 cm depth) and PAR were measured close to the outside of collars (but within any cloths used), and the PAR value was adjusted to allow for a light reduction of 7.5% through the chamber wall.

An automated dipwell logger (Hobo MX2001, Onset Computer Corporation) logged water table level every 15 minutes on the LWM pilot and on each of the Restoration (*Sphagnum*) and Control (Pasture) areas on the Carbon Farm. The WTL loggers proved unreliable at times, and infilled data for each dipwell was used for calculations in the first year at LWM and the second year at Winmarleigh. Hourly WTL for each dipwell was modelled from measured and logged data, where possible, to create a dataset for each plot. Meteorological stations (GP2, Delta T Devices Ltd., Cambridge, UK) located 100 m to the northwest of the LWM pilot and within the Carbon Farm pilot each measured air and peat temperatures (5 cm depth), rainfall, and PAR every 15 minutes. Thereby, measured CGHG fluxes and environmental variables could be modelled and the annual CGHG budget calculated.

There is no standardized approach to GHG data collection or modelling, which leads to uncertainties across the literature, and makes comparisons between results difficult (Huth et al. 2017). Chamber measurements tend to be made across the hours of brightest daylight (i.e., within two hours before and after midday), with an emphasis on the non-dormant season, incorporating a light response curve at each monitoring visit using a range of light shrouds. It is also recognised that plants and the soil microbiome respond differently depending on the time of day and that changes in water table levels do not elicit an immediate change in respiration from peat soils. This study standardized methods across all treatments on both pilot sites, with monthly visits throughout the year, taking measurements at the brightest time of day with one additional light-shroud measurement on each visit, which was the optimum method within the project constraints. This ensured an inherently wide range of temperature, light and moisture levels. In the first year of the project, a larger range of light-shroud measurements was taken during two summer measuring visits on the Winmarleigh site, which did not result in a reliable improvement in linear relationships between GPP and the primary environmental driver.

### Vegetation Measurements

#### Companion Planting Pilot (Little Woolden Moss)

The percentage cover of each *Eriophorum* species, bare peat areas and competing scrub plants across the pilot were measured in nineteen randomly-placed 2 × 2 m quadrats in areas not affected by site flooding or left deliberately bare. Measurements were made approximately yearly at 18, 32 and 43 months after planting. As the *Sphagnum* was disturbed by corvid birds immediately after planting, causing widespread failure to establish, monitoring of *Sphagnum* cover across the pilot was not possible.

Measurements of plant volume in the CGHG monitoring collars were made, both to include in calculations of the chamber headspace for each flux measurement and to establish any relationships between the volume of vegetation and the magnitude of CGHGs. During the establishment year, a pin-touch method was used (25 pins per collar). When collars were relocated, the sward within the old collars was cut and all leaves were counted, measured and the volume calculated. The pin-touch data was then calibrated against volume measurements. In the post-establishment years, on each CGHG monitoring visit, leaves were counted in each collar and 5% of the leaf lengths were measured. The volume of plant material was calculated for each collar using a pre-determined length:volume regression.

Clumps of *Sphagnum* material were taken from the pilot site and added to three of the CGHG monitoring collars at the beginning of the third year of measurements to reflect the anticipated inclusion of *Sphagnum*, although pilot-scale *Sphagnum* planting was not implemented. These clumps were measured as described below (*Sphagnum* plug area) on the Carbon Farm, and percentage cover was calculated for each collar at each monthly monitoring visit.

#### Carbon Farm (Winmarleigh Moss)

*Sphagnum* plug size was calculated by measuring plug length (*l*) and width (*w*) using the equation for an oval, incorporating the percentage cover within the plug parameters (Eq. 5).

5 $${Area}=\left(\pi \left(\frac{l}{2}* \frac{w}{2}\right)\right) \% {cover}$$

*Sphagnum* cover across the Carbon Farm was measured quarterly using twenty 1 × 1 m quadrats, placed randomly but accessibly, measuring the area of 10 plugs per quadrat and finding the average plug size, and calculating overall cover from the number of plugs in each quadrat. *Sphagnum* cover within CGHG collars was measured as a percentage cover of plugs within the collar area.

### CGHG flux measurement data management

Carbon Greenhouse Gas (CGHG) flux data were managed as described by Keightley et al. (2023), whereby *R*^2^ and *p*-values were obtained for each 2-minute flux measurement. Linear regression graphs of time and flux demonstrated where high CO_2_ uptake by plants (reducing chamber CO_2_ sufficiently to affect plant uptake), a rapid change in PAR, or equipment malfunction had caused an artificial non-linearity, when the data section could be safely removed (Renou-Wilson et al. 2019). The maximum number of observations (seconds) retained per measurement was 124 and the minimum number was 60, apart from 4.9% (LWM) and 1.8% (Winmarleigh) of measurements in semi- or full light, when representative measurements were less than 60 observations due to conditions described above. Data were accepted if either CO_2_ or CH_4_ fluxes (as they were measured together) met thresholds of *R*^2^ > 0.7 and *p* < 0.05 (Evans et al. 2016), which would negate a system error and accommodate low *R*^2^ values when fluxes were small, e.g., in cold weather or low light. Only 1 flux measurement was discarded from the LWM pilot control, and 5 outliers from the Winmarleigh pilot site due to disturbance by outside factors. The micrometeorological sign convention was adopted, whereby negative fluxes indicate removal from the atmosphere and positive fluxes indicate addition to the atmosphere.

Fluxes were calculated using the following equation (Eq. 6) adapted from Dossa et al. (2015):

6 $${Flux}=\frac{\Delta {CO}2}{t}* \frac{{PV}}{{RT}}* \frac{1}{{As}}* \left(\frac{44* 60* 60}{1000}\right)$$

[Flux = g CO_2_ (or CH_4_) m^−2^ h^−1^; P (atm) = atmospheric pressure; V (m^3^) = chamber headspace volume; R (L atm mol^−1^ K) = universal gas constant; T (K) = gas temperature in Kelvin; A_s_ (m^2^) = surface area within collar; 44 g mol^−1^ = molecular weight of CO_2_ (or 16 g mol^−1^ = molecular weight of CH_4_)].

Methane fluxes (FCH_4_) were converted to CO_2_ equivalents (CO_2_e) using GWP_100_ = 28 (Myhre et al. 2013) (i.e., CH_4_ x 28) as adopted in the 1997 Kyoto Protocol.

### CGHG Flux Modelling Methods

Empirical modelling was used to calculate annual CGHG budgets, which employed a basic, iterative process of incorporating environmental drivers of measured GHG flux, extrapolated into a predictive model to calculate an annual CO_2_e budget (as described by Keightley et al. [2023], and similar to methods employed by Creevy et al. [2020]), using hourly environmental variables measured by the on-site micro-meteorological station and WTL loggers. Microsoft Excel (2019) was used throughout. Probable primary, secondary and tertiary environmental drivers were identified using best-fit regression equations. The primary driver regression reflected a realistic flux trajectory, e.g., did not predict a negative value for RECO at the intersect, and an exponential curve resolved this potential problem on occasions (15% of all primary drivers). The primary driver best-fit regression equation was used to create a primary model. These values were subtracted from the measured data, and the residual data fitted against the secondary driver using a linear or polynomial order 2 regression to produce a second model. This process continued to produce a third model if necessary. At each stage, the modelled values were fitted against the measured values to check for a continually improving linear regression *R*^2^ value and trendline gradient, and the process continued with each driver until there was no improvement. Different orders of drivers, and regression equations of the primary driver (exponential, polynomial orders, etc.) were tried to find the best fit throughout. These regression equations were then fitted against an hourly data set from the site meteorological station and WTL logger data to provide an annual budget for each flux.

FCH_4_ values were very small with low variability, or appeared as both emission and uptake across plots, and could not be modelled individually. Therefore, measured FCH_4_ (as CO_2_e) and RECO values were combined before modelling (and actually improved the *R*2 value against environmental variables compared to RECO alone) to create a combined Emissions model. Measured GPP was calculated from NEE and RECO measurements, and modelled GPP values were converted to zero when PAR was zero. GPP could not be calculated on LWM Bare peat plots; therefore, NEE was modelled directly, and Emission values were used when PAR was zero. Other than LWM Bare peat plots, annual CO_2_e budgets were calculated from the addition of Emission and GPP annual budgets. A different model was needed for each flux dataset, which accommodated the high variability in biotic and abiotic conditions. The relationship between graphed measured and modelled data was carefully examined for measured data lying sensibly within modelled parameters to produce annual flux budgets (examples in Fig. S1).

Annual CGHG budgets were calculated using both the modelled combined data from all collars and the mean of CGHG collars modelled individually (e.g., as for Renou-Wilson et al. [2019]), for each full monitoring year. As plots varied widely in terms of vegetation cover (particularly on restoration plots) and WTL, individual plot data tended to model more closely with environmental variables than collated plot data, and subsequently, a closer relationship between modelled and measured data for individual plots was found compared to combined plots. Statistically significant differences between modelled and measured data were tested (Table S2). There was less difference statistically between modelled and measured values on an individual plot basis than on a combined plot basis across all pilot values. The percentage of *p*-values at < 0.05 (Emissions and GPP/NEE, respectively) for combined data was 33.3% and 58.3% and for individual plot data was 0% and 20.8%.

Plot data was also used to assess the influence of vegetation change and/or maturity and variation in WTL on carbon balance over time. WTL from the dipwell logger data was calibrated to measured data to give a continuous hourly dataset for each collar dipwell.

A carbon balance for each monitoring year was also derived by using a calculation from measured values devised by the Care Peat Project, and which may be useful as a rudimentary check or a general guide during the modelling process to determine whether values are within an appropriate range. It used the annual median for each type of measured flux in g m^−2^ h^−1^. RECO and FCH_4_ (as CO_2_e) were combined into CO_2_e emission values, as above, and NEE in both semi- and full light were averaged to give CO_2_ uptake values. Medians were multiplied by 12 (which is an average for either day or night length in hours across the year) and multiplied again by 365 to calculate the net amount of CO_2_ uptake and emission per year. The combination of uptake and emission gave a yearly CO_2_e budget.

### Data analysis

Data were analyzed using Microsoft Excel 2019 and PAST: Palaeontological Statistics Software Package for Education and Data Analysis (Hammer et al. 2001), where indicated. Data were tested for normality using the Shapiro–Wilk test. Data were found to be non-normally distributed throughout and non-parametric Mann-Whitney and one-way ANOVA tests (Kruskal-Wallis) with Dunn’s post-doc tests were used where appropriate to test statistically significant differences. The dependency of plant volume on WTL and peat temperature was tested using linear regression. Statistical significance was determined with a *p*-value < 0.05.

## Results

### Companion Planting Pilot (Little Woolden Moss)

#### Water Table Level and Environmental Variables

**Fig. 5:**
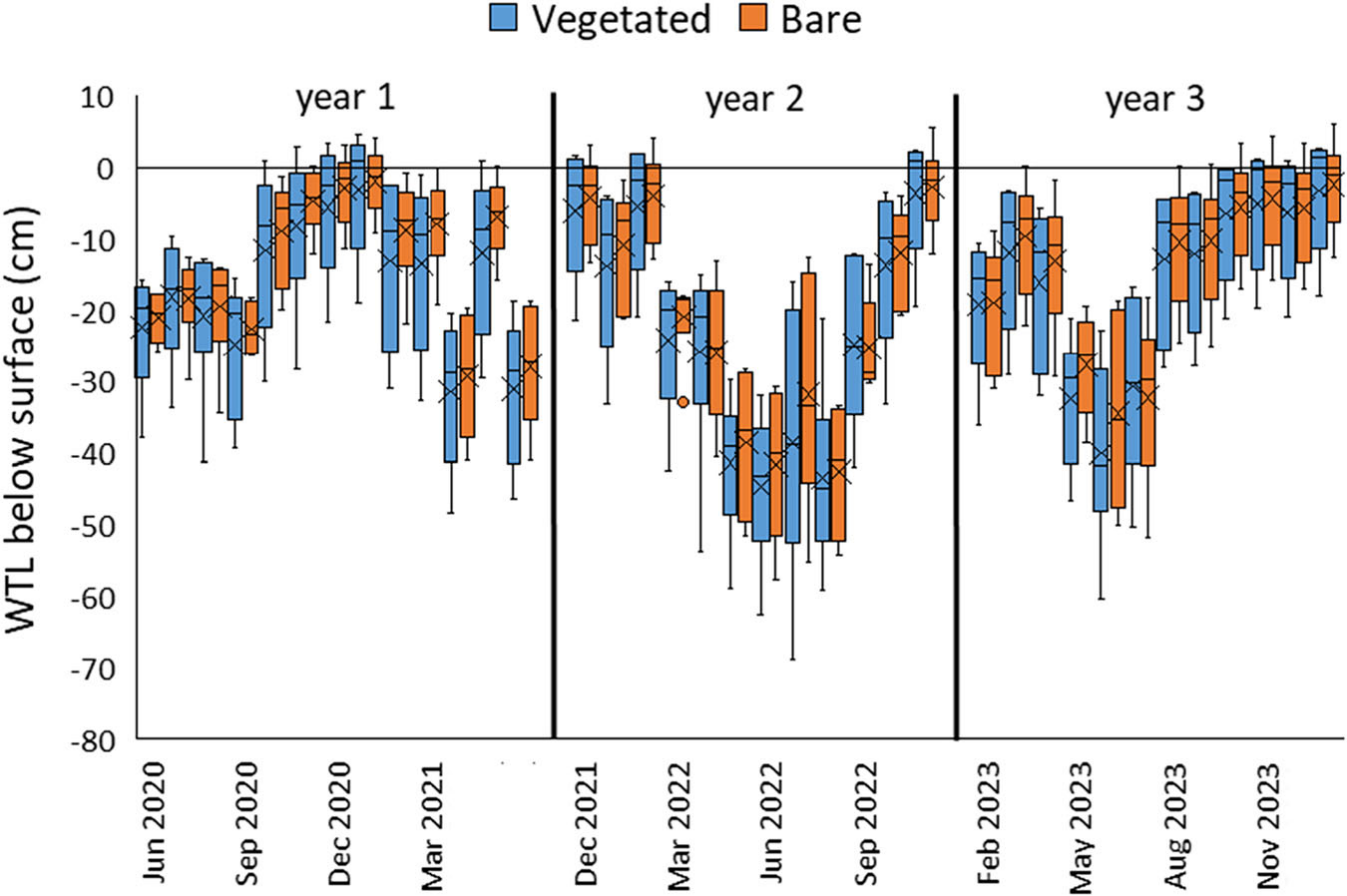
Monthly Water table level (WTL) measurements on the LWM companion planting pilot at CGHG monitoring collars, showing seasonal variation and wide variation between monitoring points; box plots show collated plot data, crosses indicate the mean value, lines indicate the median, and the interquartile range is exclusive

**Fig. 6:**
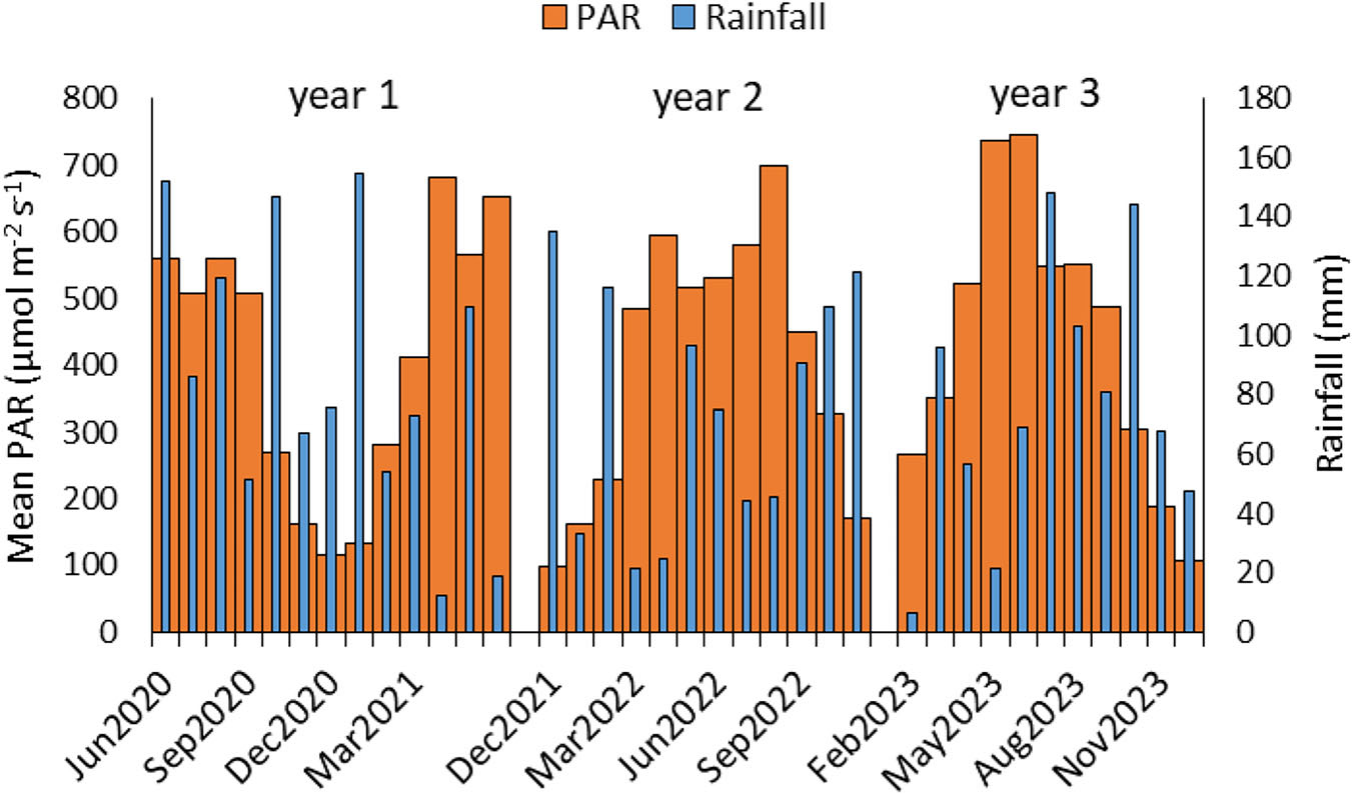
Monthly mean PAR and mean Rainfall per month over the 3-year measurement period on the LWM pilot

Water table level below the surface (WTL) followed expected seasonal fluctuations (Fig. 5) with measurements in the Bare (Control) area being slightly higher than the Vegetated (Restoration) area (−16.6 ± 14.2 and −18.9 ± 15.9, respectively, mean ± SD), but with no statistically significant difference overall. Due to the positioning of monitoring points in both ridges and depressions, there was a wide range of water table levels between them. Compared to the long-term average (LTA) values of rainfall and air temperature (AT) for the area, all years had higher rainfall, particularly year 1, and year 2 July AT was higher and years 1 and 3 lower than the LTA (Table 1). There were several dry periods: April 2021, summer 2022, and early summer 2023, and periods of high rainfall (outside of cooler seasons) were often coupled with low PAR due to cloudiness (Fig. 6). Water table levels in the establishment year (year 1) of CGHG monitoring (June 2020 to June 2021) were higher overall (Vegetated: −16.7 ± 13.1, min: −48.2 cm; Bare: −13.8 ± 10.9, min: −40.9 cm) than in year 2 (December 2021 to November 2022) (Vegetated −23.7 ± 18.5, min: −68.9 cm; Bare: −21.6 ± 16.5, min: −57.6), with a long period of high rainfall in the latter part of in year 3 (February 2023 to January 2024) leading overall to similar mean levels to year 1 (Vegetated: −16.3 ± 14.9, min: −60.3 cm; Bare: −14.5 ± 13.5, min: −51.6 cm) (mean ± SD and minimum, i.e., lowest level). Differences within treatments between year 2 and years 1 and 3 were statistically significant on Dunn’s post-hoc tests (Vegetated *p* < 0.05; Bare *p* < 0.01), whereas there was no significant difference between years 1 and 3.

**Table 1:** Meteorological conditions for the LWM site area: long-term average and each monitoring year

	Area LTA^a^	Site year 1	Site year 2	Site year 3
Annual Rainfall (mm)	868	1102	916	933
Mean Annual AT^b^ °C	9.7	9.4	10.8	10.8
Mean January AT °C	4.1	2.7	3.4	5.0
Mean July AT °C	16.2	14.4	17.4	15.7

^a^Long-term average (LTA) weather data between 1991 and 2020 was derived from the nearest Met Station at Woodford 53°20'24.0“N 2°09'14.4“W 88 m asl 23.8 km SE (Met office, 2022); ^b^AT = air temperature. Year 1: from June 2020, Year 2: from December 2021, Year 3: from February 2023: measured from the site met station

#### Peat Quality and Carbon Stock Measurements

**Fig. 7:**
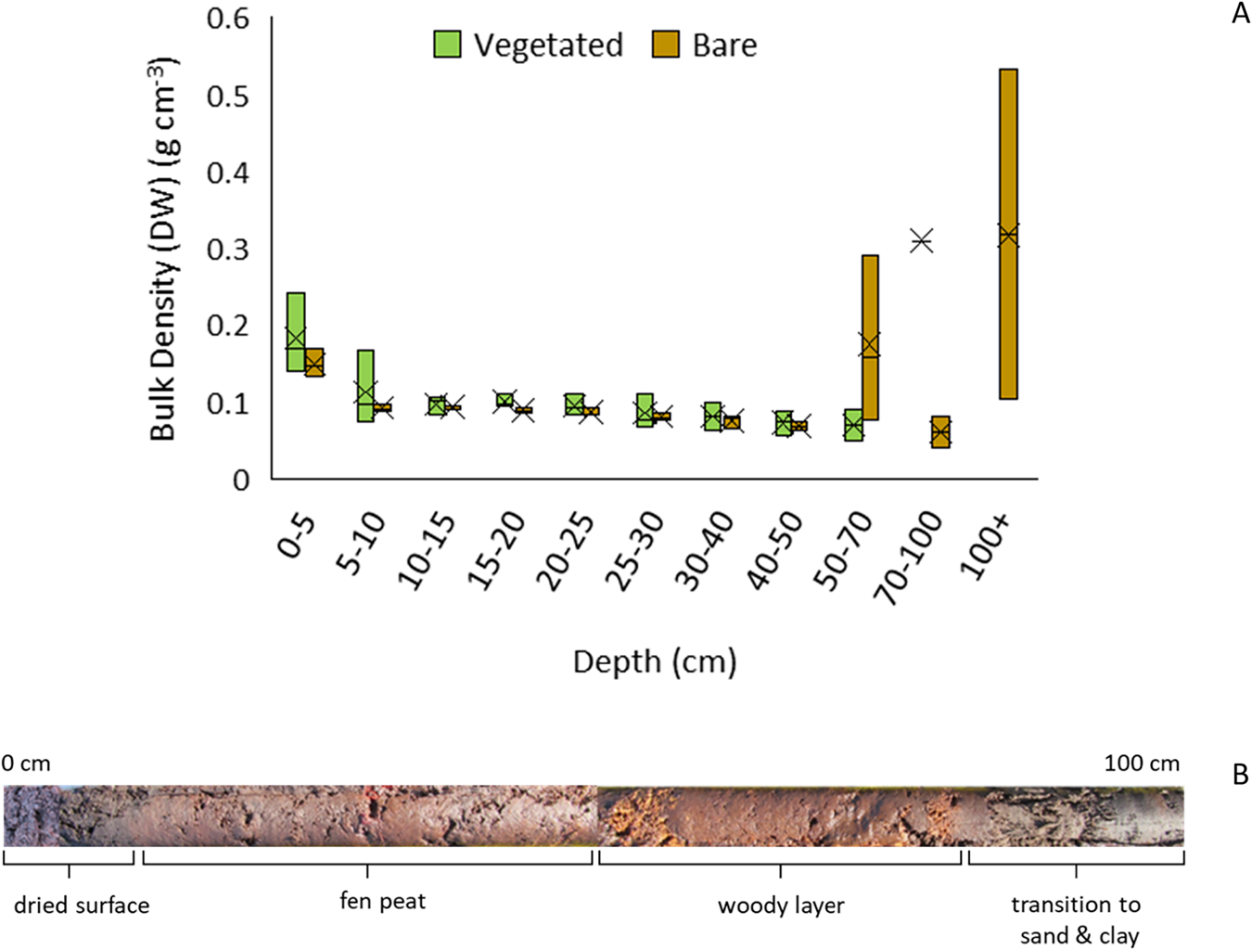
**A**: Bulk density (dry weight per volume) of peat core samples in each monitoring area (*n* = 3); **B**: composite image of typical peat core showing change in peat quality from surface to mineral substrate

The peat surface layer was highly decomposed, and variously hard or friable, with a higher bulk density (Fig. 7A) than the underlying layers of fen peat and wood, until the mineral substrate (sand and clay) was reached at between 55 and 125 cm depth (Fig. 7B). The total remaining carbon stock (organic material above the mineral layer; wood particles were removed from samples) on the Vegetated and Bare peat areas was calculated as 312 ± 126 and 390 ± 114 t ha^−1^ (mean ± SD) respectively (Vegetated plots were on slightly shallower peat than Bare peat plots).

#### Vegetation Measurements

*Eriophorum* species (particularly *E. angustifolium*) rapidly spread across the bare peat (Fig. 19 [Appendix]). After 18 months, over half of the site was vegetated, increasing at a similar rate in the second year and slowing considerably in the third year as the mature *E. angustifolium* plants senesced and regrowth slowed. However, ratios of the original 3:1 *E. angustifolium* to *E. vaginatum* had increased to 10:1 after 3 years (Table 2).

**Table 2:** Results of vegetation monitoring (19 survey plots) on LWM companion planting pilot in 2021 (September), 2022 (November) and 2023 (October) (18, 32 and 43 months after planting, respectively) (Mean ± SD)

Mean % cover	2021	2022	2023
*E. angustifolium*	49.1 ± 28.2	72.3 ± 27.6	78.2 ± 23.6
*E. vaginatum*	6.7 ± 5.2	8.8 ± 9.5	7.7 ± 7.8
Bare peat	44.2 ± 26.9	19.6 ± 24.8	15.2 ± 22.9
*Betula sp*.	0.0 ± 0.0	0.5 ± 1.2	4.4 ± 7.9

By 2023, 9 plots had live plants throughout, and the remainder were in various stages of plant senescence

**Fig. 8:**
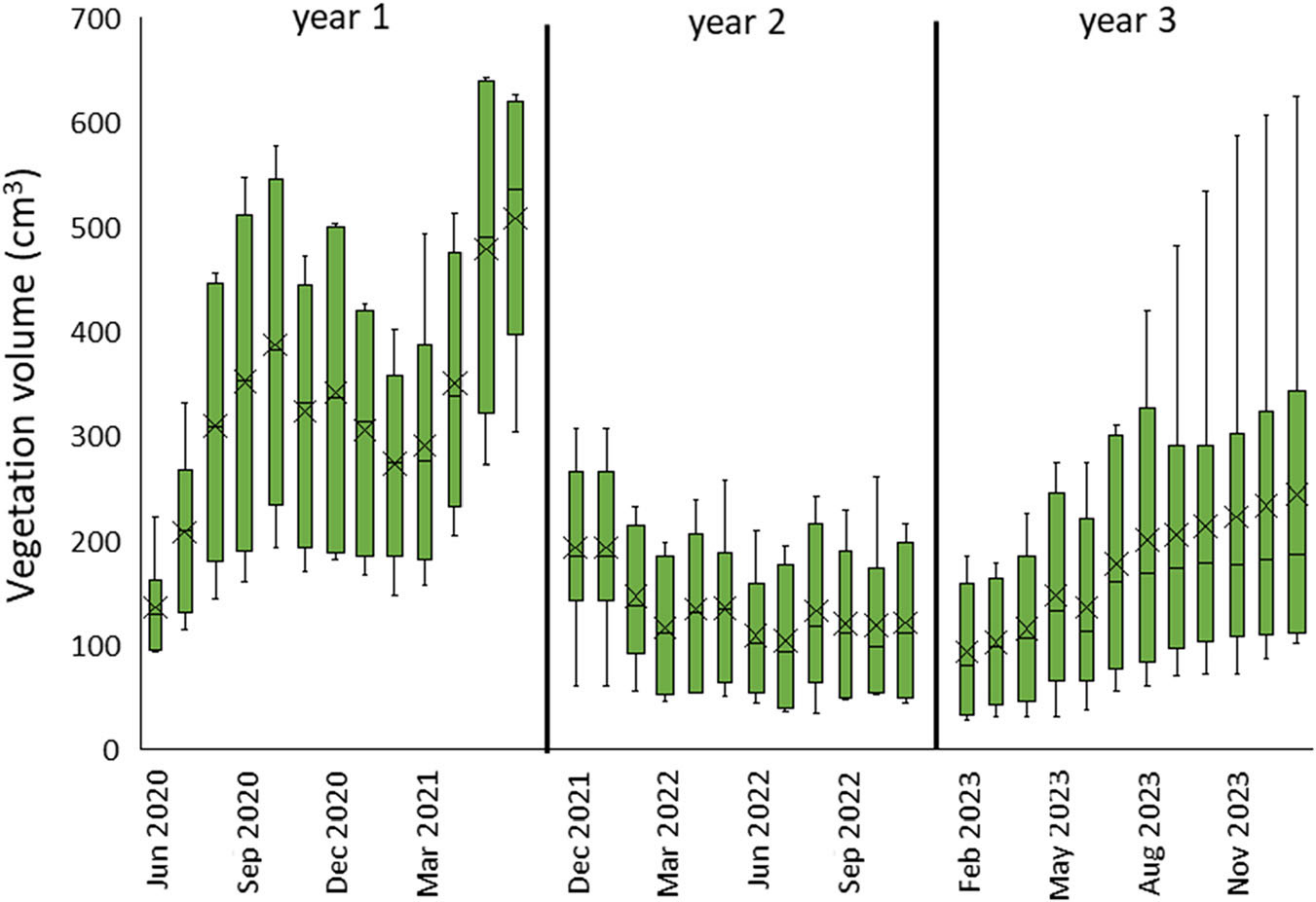
Monthly measured vascular plant cover within the CGHG monitoring collars, separated into the ‘establishment’ phase (year 1) (*E. angustifolium* and *E. vaginatum* growing from plug plants, planted at 3:1 ratio) and subsequent mature years 2 and 3 following collar relocation into mature stands of *E. angustifolium*; box plots show collated plot data, crosses indicate the mean value, lines indicate the median, and interquartile range is exclusive

There was a wide variation in the CGHG collar vascular plant volume between plots throughout (Fig. 8), and the mean volume was significantly higher in the establishment year (*E. angustifolium* and *E. vaginatum* combined), when plants were vigorously growing, than in subsequent years, with statistically significant differences between year 1 and years 2 and 3 on Dunn’s post-hoc tests (*p* < 0.001) but not between years 2 and 3. In the second year of measurements, after the collars had been moved to mature stands of *E. angustifolium* (Fig. 8), there was also no expected increase in plant volume during the growing period. There was early senescence of plant material during a 2022 summer drought (Fig. 6) and, moreover, the depth of plant litter increased from 0.5 ± 0.2 cm in December 2021 to 2.2 ± 0.5 cm in July 2022, changing little thereafter. In the third year, the volume of vascular plant material increased over the growing period, with a slight reduction due to dry conditions in early summer, although differences between collars was increasingly large as the year progressed due to high plant die-off/senescence in some plots. Regression models between plant volume and WTL showed small positive relationships between higher plant volume and higher (i.e., nearer the surface) WTL (*R*^2^ = 0.12, *p* < 0.01; 0.22, *p* < 0.001; 0.22, *p* < 0001 for years 1, 2 and 3, respectively), but there was no relationship between plant volume and peat temperature.

There was no significant difference in the peat moisture content due to the presence or not of *Sphagnum* in collars in year 3, and although *Sphagnum* appeared to support *E. angustifolium* growth in terms of volume, the difference between plots with (190 ± 172 cm^3^) and without (159 ± 83 cm^3^) *Sphagnum* was not statistically significant (mean ± SD). The mean cover of *Sphagnum* within collars in year 3 was 73 ± 17%, reducing from 90% cover overall in the spring to below 60% by the autumn due to either summer desiccation or increasing shade competition from *Eriophorum*.

#### Measured CGHG Flux

**Fig. 9:**
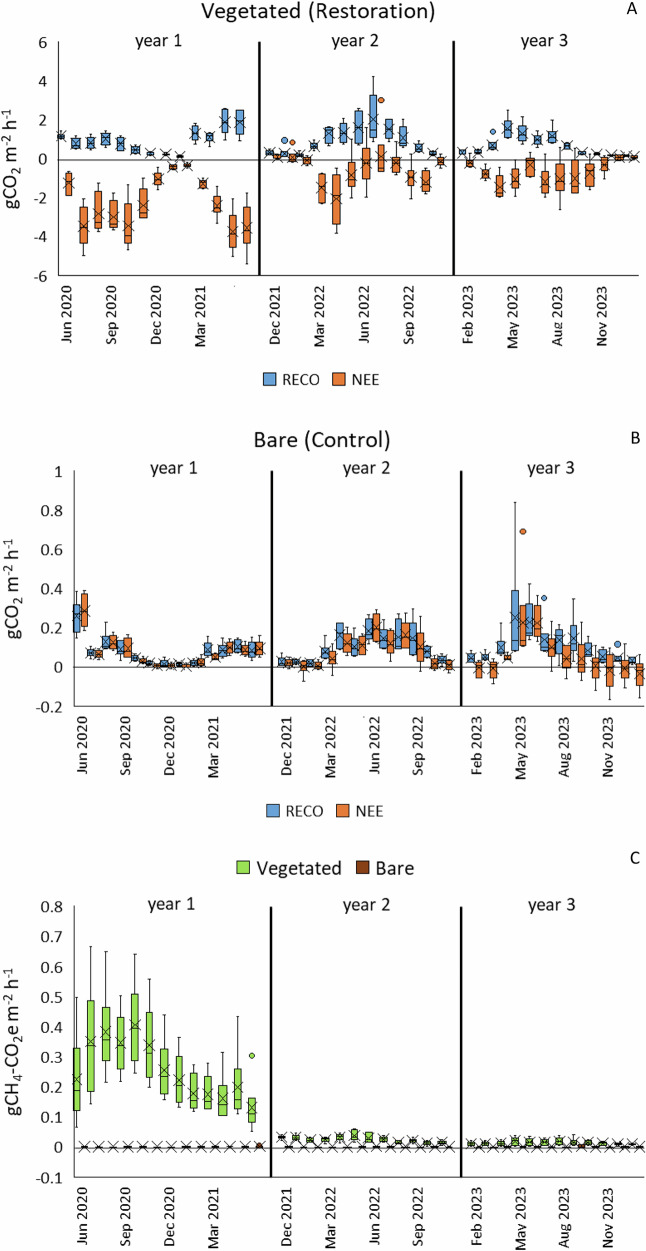
Measured CGHG data from the LWM Companion Planting pilot in the ‘establishment’ phase (year 1) and post-establishment years (years 2 and 3): **A** RECO and NEE (full light only) on Vegetated (Restoration) plots; **B** RECO and NEE (full light only) on Bare (Control) plots; **C** methane flux on both treatments, converted to CO_2_ equivalents; box plots show collated plot data; crosses indicate the mean value, lines indicate the median, and interquartile range is exclusive

Rapid vascular plant growth promoted a greater NEE (CO_2_ uptake) than RECO (CO_2_ emission) (Fig. 9A) in the Restoration ‘establishment’ phase (year 1). During the post-establishment phase, RECO was the stronger flux for much of the year (i.e., resulting in overall emission), with NEE CO_2_ emissions from some collars during warmer, drier months. Across all vegetated plots in years 1, 2 and 3 respectively, RECO (mean ± SD) was 0.89 ± 0.63, 0.91 ± 0.79 and 0.65 ± 0.54 g CO_2_ m^−2^ h^−1^ (differences were not statistically significant), and NEE (in full light) was -2.25 ± 1.41, −0.59 ± 1.03 and −0.67 ± 0.74 g CO_2_ m^−2^ h^−1^ (statistically significant differences between year 1 and years 2 and 3 (*p* < 0.001) but not between years 2 and 3).

CO_2_ emissions were an order of magnitude lower but continuous from bare peat (Control) throughout the monitoring period, rising with warmer, drier conditions (Fig. 9B), becoming larger and more variable overall in the post-establishment years compared to the establishment year, with a small CO_2_ uptake in some plots in the 3rd year. Where a higher NEE than RECO emission occurred, it was likely due to a slightly warmer chamber during measurements in the light (gas temperature during warmer months, May to September, 24.3 ± 4.5 °C and 24.9 ± 3.8 °C, dark and light, respectively, mean ± SD), which increased respiration. Across all Bare plots in years 1, 2 and 3 respectively, the pooled RECO and NEE (in full light) were 0.076 ± 0.075, 0.086 ± 0.076 and 0.079 ± 0.118 g CO_2_ m^−2^ h^−1^ (mean ± SD, no statistically significant differences).

Measured CH_4_ fluxes as CO_2_ equivalents (using GWP_100_ = 28) in the establishment year across vegetated plots were much higher than in subsequent years: 0.26 ± 0.14, 0.025 ± 0.012 and 0.013 ± 0.009 g CO_2_e m^−2^ h^−1^ (mean ± SD) in years 1, 2 and 3 respectively (statistically significant differences between year 1 and years 2 and 3 (*p* < 0.001) but not between years 2 and 3). These fluxes were negligible in bare plots: 4.0^−4^ ± 7.5^−4^, 1.6^-4^ ± 8.3^−4^ and 4.3^−4^ ± 1.4^−3 ^g CO_2_e m^−2^ h^−1^ in years 1, 2 and 3, respectively (differences were statistically significant between years 1 and 2 only at *p* < 0.05) (Fig. 9C).

#### Measured Flux in relation to environment and plant variables

Regression models between measured fluxes and environmental variables across the whole monitoring period showed greater RECO fluxes with increasing water table level (WTL) below the surface (i.e., deepening) and increasing peat soil temperature (TS), on both treatments and greater GPP with increasing TS and PAR on Vegetated plots (there was no or negligible CO_2_ uptake on Bare plots) with a minimal influence of WTL. The linear regression *R*^2^ values for WTL and TS, respectively, against RECO were 0.21 and 0.40 (Vegetated) and 0.28 and 0.34 (Bare); the *R*^2^ values for WTL, TS and PAR, respectively, against GPP were 0.02, 0.26 and 0.37 on Vegetated plots, with *p* < 0.001 throughout apart from WTL against GPP, where *p* < 0.05.

There was no strong or even consistent relationship between the volume of plant material and CGHG fluxes on an overall basis. The addition of *Sphagnum* to plots in year 3 made no statistically significant difference to RECO, GPP or FCH_4_ flux compared to those without, although differences in *E. angustifolium* volume and health were a likely confounding factor.

In the first year, when plant growth was rapid, particularly on plots with a higher WTL, regression models showed increasing RECO and GPP with increasing vegetation volume (*R*^2^ = 0.32 and 0.35, respectively), but these relationships did not continue into subsequent years. Relationships between FCH_4_ and vegetation volume were slight overall. RECO tended to slightly increase with increasing plant volume in year one, but decrease in subsequent years, apart from one plot with significant plant senescence where RECO increased greatly along with increasing plant volume in year 3 (*R*^2^ = 0.65). GPP increased with plant volume in year one but generally decreased in year 2 (summer drought), then increased again in year 3, apart from the plot with significant plant senescence. FCH_4_ was inconsistently related to vegetation volume in year 1, but increased with plant volume in subsequent years, particularly in the two drier plots in year 3 (*R*^2^ = 0.86 and 0.87).

#### Modelled CGHG Flux Budget

RECO plus FCH4 (as combined emission values) was modelled using peat temperature (TS) as a primary driver and WTL as the secondary driver. Primary, secondary, and tertiary drivers of Gross Primary Productivity (GPP) or Net Ecosystem Exchange (NEE) fluxes (as appropriate) varied between collars, and a breakdown of the drivers with *R*^2^ values of linear regression equations used for modelling are shown in Table 6 (Appendix). GPP could not be modelled for Bare plots, and so NEE was modelled directly. The CGHG balance figures derived from median measured values (Table 7 [Appendix]) show the same general trajectory and a similar range across the project as the modelled values.

The major difference in yearly CGHG budgets was a large CO_2_e uptake in the establishment year in the vegetated plots followed by large CO_2_e emissions in subsequent years, particularly the drier year 2, whether modelled with combined collar data or the mean of individual modelled collar data (Table 3). The anomaly was plot 2 (situated on an old raised peat bed), which had large CO_2_e emissions in year 1, contrary to all other plots, and reduced emissions thereafter, related to improved vascular plant growth, which influenced GPP (Table 3; Fig. 20 [Appendix]). Addition of *Sphagnum* into three collars in year 3 appeared to have little effect on CO_2_e fluxes, which were mainly driven by vascular plant volume and health. Plot 1 was the only collar with a CO_2_e uptake in year 2, but additional *Sphagnum* in year 3 did not result in a continued uptake. Conversely, *Sphagnum* addition in plot 4 appeared to help reduce emissions to far lower levels in year 3 than year 2, although it was also concurrently supported by improved growth of *E. angustifolium*. Year 3 CO_2_e flux in Plots 5 and 6 were similarly influenced by *E. angustifolium* growth, with large improvements in plot 5 (coupled to reduced RECO in year 3) and early plant senescence increasing emission in plot 6 (Table 3; Fig. 20 [Appendix]).

**Table 3:** Annual modelled CGHG balance (t CO_2_e ha^−1^ y^−1^) at the LWM site for each treatment in each monitoring year (sum of hourly modelled values for each year) with associated Carbon losses (t C ha^−−1^ y^−1^) (CGHG balance x ^12^/_44_)

	Restoration (Vegetated)						Control (Bare)					
	Year 1		Year 2		Year 3		Year 1		Year 2		Year 3	
	CGHG	C loss	CGHG	C loss	CGHG	C loss	CGHG	C loss	CGHG	C loss	CGHG	C loss
Combined data	−33.17	0.00	39.58	10.80	29.73	8.11	4.10	1.12	8.63	2.35	8.00	2.18
Plot 1	−19.61	0.00	-4.01	0.00	9.16	2.50	3.23	0.88	8.76	2.39	4.89	1.33
Plot 2	31.18	8.50	9.76	2.66	14.61	3.98	4.42	1.20	4.36	1.19	2.30	0.63
Plot 3	−4.05	0.00	20.46	5.58	19.96	5.44	4.20	1.14	9.92	2.71	9.18	2.50
Plot 4	−48.64	0.00	31.54	8.60	4.59	1.25	3.61	0.98	5.38	1.47	4.92	1.34
Plot 5	−59.37	0.00	73.64	20.08	17.65	4.81	6.99	1.91	11.80	3.22	13.53	3.69
Plot 6	−33.98	0.00	25.13	6.85	32.47	8.86	3.34	0.91	4.57	1.25	6.34	1.73
Mean plot data	−22.41	1.42	26.09	7.30	16.41	4.47	4.30	1.17	7.47	2.04	6.86	1.87
±SD	32.88	3.47	26.44	6.97	9.67	2.64	1.40	0.38	3.13	0.85	3.97	1.08
						*Sphagnum* added to collars						

Values shown as modelled from collated plot data, and modelled from individual plot data Year 1: *E. angustifolium* and *E. vaginatum* (plug plants 3:1 ratio) developing from 2 months after planting to mature stands; years 2 and 3: mature *E. angustifolium* only; year 3 *Sphagnum* clumps added to collars 1, 2 and 4. Negative values are CO_2_e uptake and positive values are CO_2_e emission

### Carbon Farm Pilot (Winmarleigh Moss)

#### Water table level and environmental variables

**Fig. 10:**
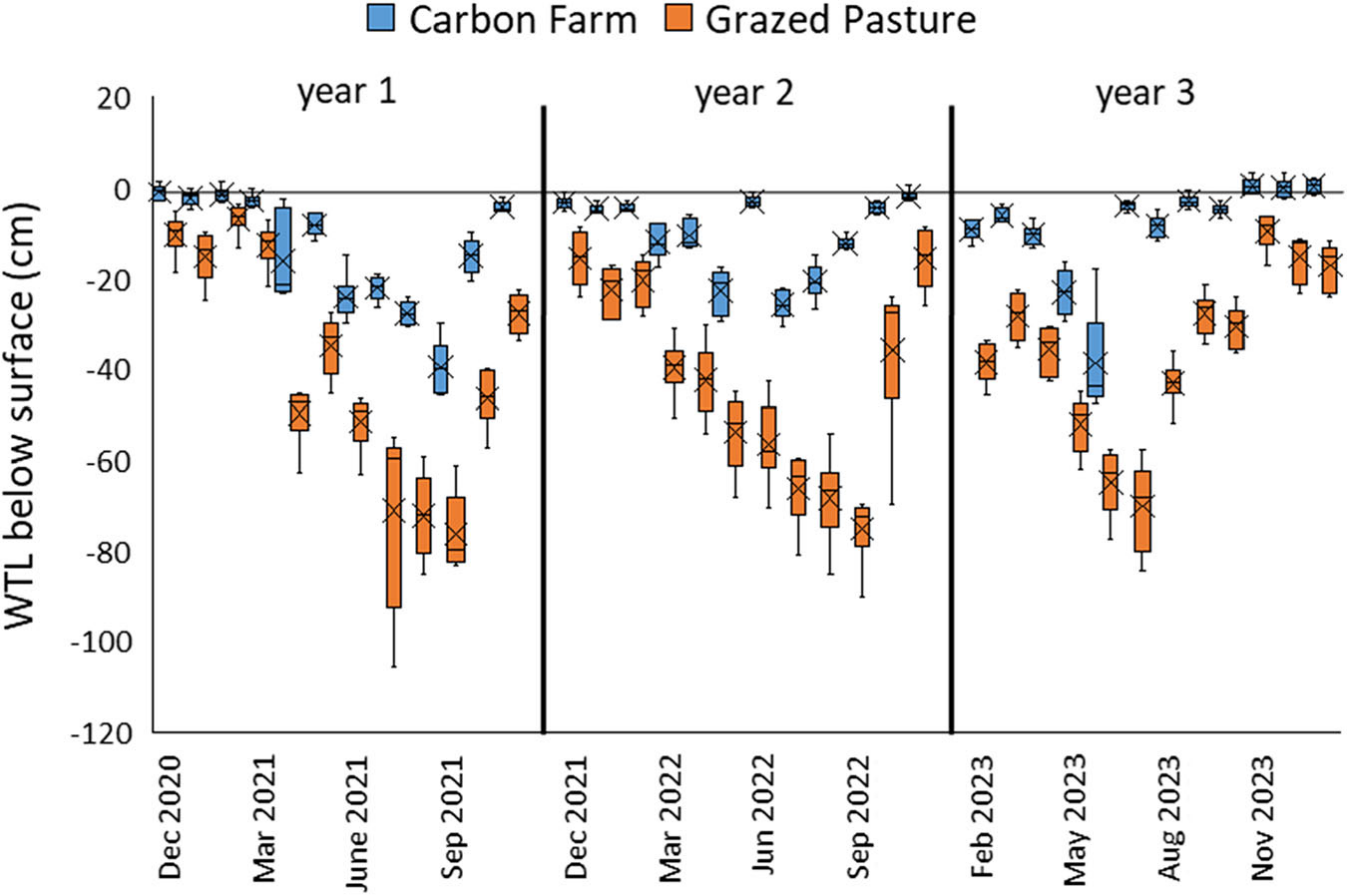
Monthly measured water table level below the surface (WTL) at CGHG monitoring collars on the Carbon Farm pilot, showing seasonal variation and wide differences between levels on the Carbon Farm (Restoration) and Grazed Pasture (Control); box plots show collated plot data, crosses indicate the mean value, lines indicate the median, and the interquartile range is exclusive

Creation of the ‘Carbon Farm’ through conversion from drained, grazed pasture to re-wetted peat with *Sphagnum* resulted in continually higher groundwater levels throughout the monitoring period than on the adjacent drained pasture: −11.0 ± 11.2 cm and −39.3 ± 22.4 cm respectively (U = 5453.5, *p* = <0.001, *n* = 216) (Fig. 10). Improvements in water availability to the Carbon Farm in late summer 2021 resulted in an immediate rise of the peat surface due to swelling of the peat (5.2 ± 3.3 cm mean ± SD), recorded during measurements from the ground to the top of dipwells (Fig. 21 [Appendix]).

WTL in years 1, 2 and 3 respectively on the Carbon Farm were (mean ± SD and minimum i.e., lowest level): −13.7 ± 12.7, min −45.8 cm; −10.4 ± 8.5, min −30.5 cm; −8.9 ± 11.6, min −47.9 cm; and on the Grazed Pasture were: −39.6 ± 25.7, min −105.7 cm; −42.1 ± 21.9, min −90.3 cm; −36.1 ± 19.1, min −84.5 cm. Differences were statistically significant between the Carbon Farm and Grazed Pasture in each year (*p* < 0.001 throughout on Dunn’s post-hoc tests) but not within each treatment between any of the three measurement years.

**Fig. 11:**
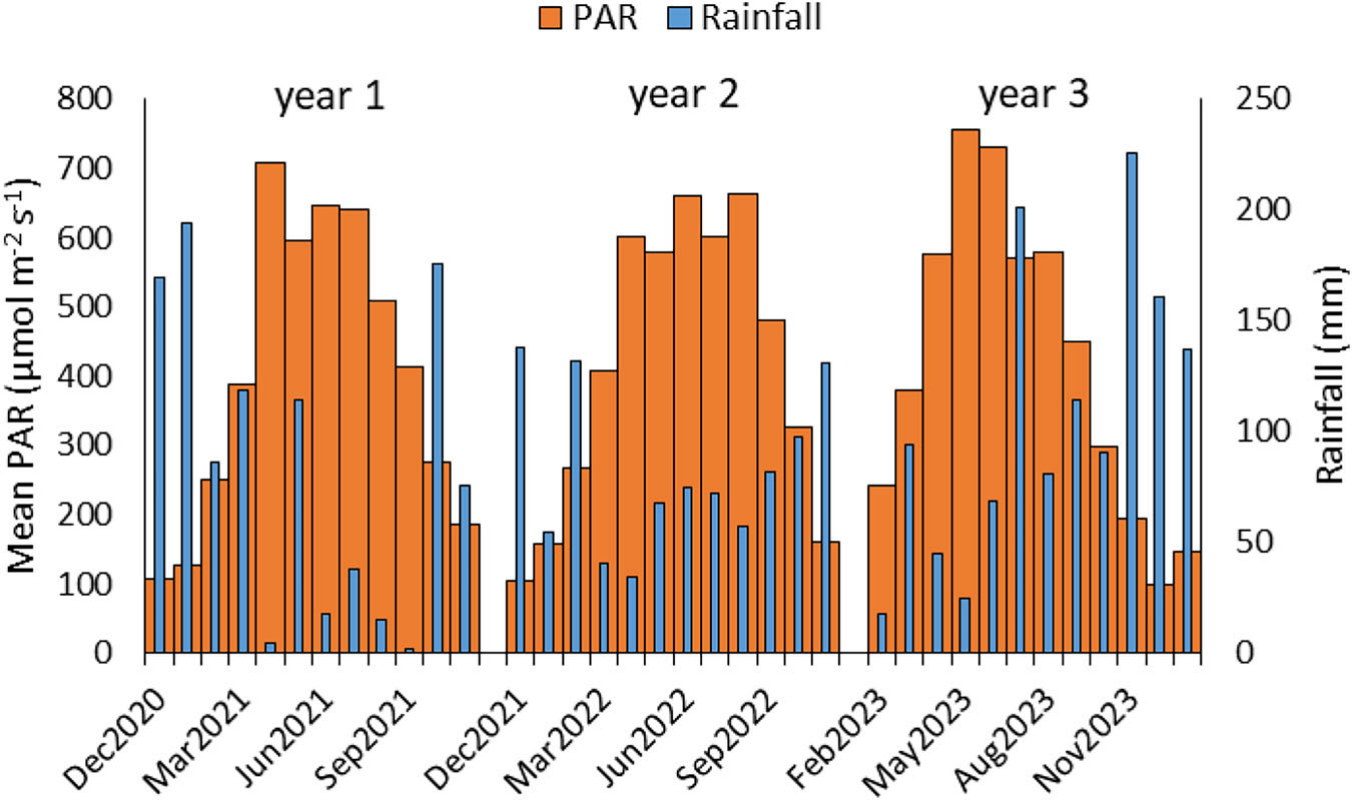
Monthly mean PAR and mean Rainfall per month over the 3-year measurement period on the Carbon Farm pilot

Rainfall was lower in the first two years, and higher in year 3 than the long-term averages for the area, and the mean air temperature in year 2 was noticeably higher (Table 4). Very dry periods in spring and summer in years 1 and 2, and spring in year 3 (Fig. 11) and occasional technical issues with the irrigation system reduced optimum moisture levels overall on the Carbon Farm. However, enlargement of the sump area in late summer 2021 and clearing irrigation ditches in spring 2023 provided a more reliable water supply. Along with regular rainfall over summer and autumn in year 3 (Fig. 11), this ensured groundwater levels were generally higher as the project progressed and more regularly close to the target of 10 cm below the peat surface. Some periods of high rainfall (outside of cooler seasons) were coupled with low PAR due to cloudiness (Fig. 11), but this was not as marked as on the LWM site.

**Table 4:** Meteorological conditions for the Winmarleigh site area: long-term average and each monitoring year

	Area LTA^a^	Site Year 1	Site Year 2	Site Year 3
Annual Rainfall (mm)	1041	1011	980	1259
Mean Annual AT^b^ °C	10.0	9.8	14.4	11.9
Mean January AT °C	4.6	2.9	5.2	6.4
Mean July AT °C	16.4	17.0	17.4	17.1

^a^Long-term average weather data between 1991 and 2020 was derived from the nearest Met Stations at Myerscough 53°51'13.0“N 2°45'55.5“W 14 m asl 9.74 km SE of pilot, and Morecambe 54°04'26.4“N 2°51'49.0“W 3 m asl 16.62 km N (Met office, 2022); ^b^AT = air temperature. Year 1: from December 2020 Year 2: from December 2021 Year 3: from February 2023: measured from site met station

#### Peat quality and carbon stock measurements

**Fig. 12:**
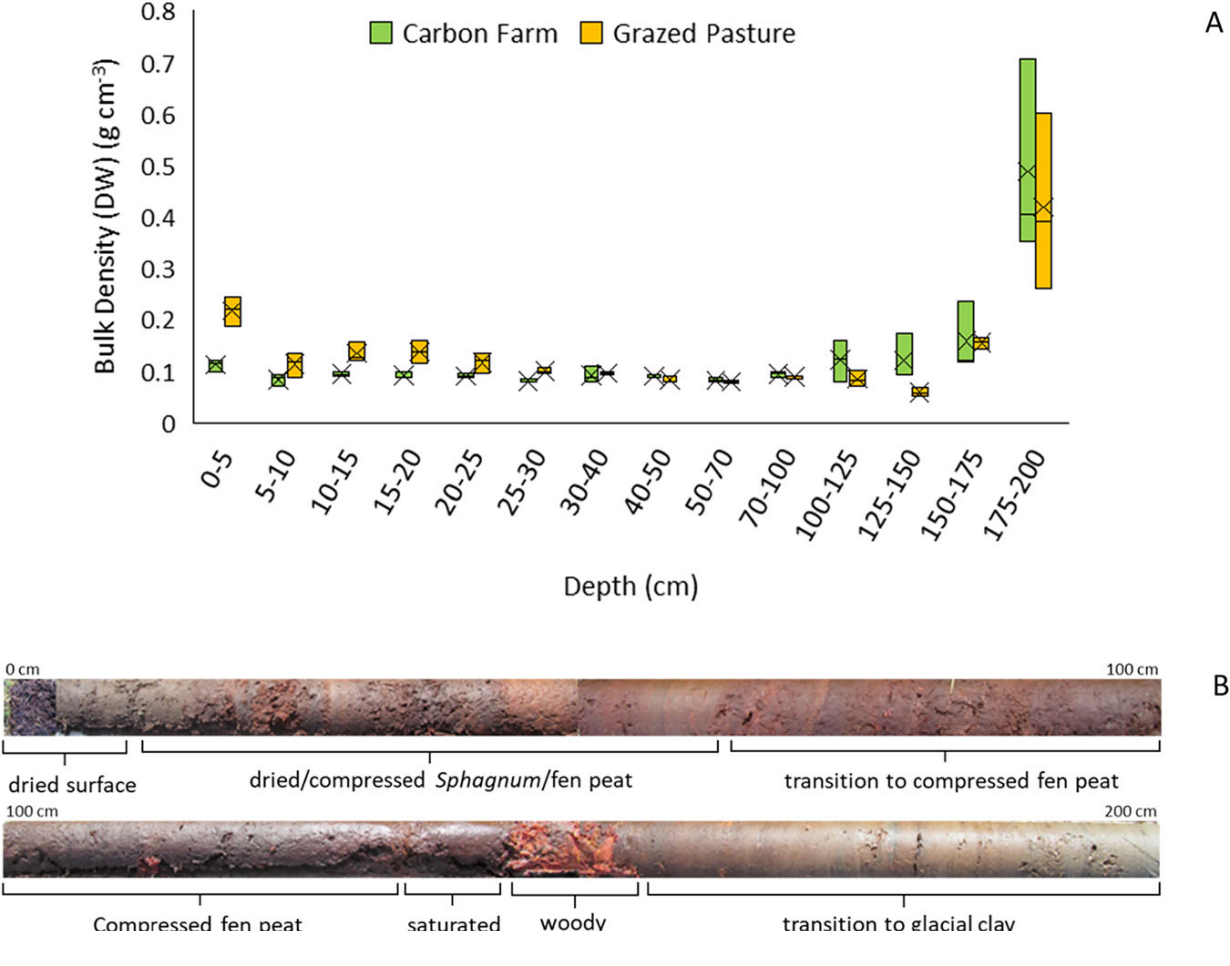
**A** Bulk density (dry weight per volume) of peat core samples in each monitoring area (*n* = 3); **B** composite image of typical peat core showing change in peat quality from surface to mineral substrate

The peat surface layer was humified with a higher bulk density in the Grazed Pasture than the Carbon Farm within the top 40 cm (Fig. 12A). Subsurface layers transitioned through more heterogeneous layers of *Sphagnum* and fen peat, which were compressed throughout. The peat was saturated at the base above a woody layer, which transitioned into glacial clay at between 150 and 175 cm depth (Fig. 12B). The remaining total carbon stock on the Carbon Farm and Grazed Pasture areas was calculated as 844 ± 115 and 692 ± 29 t ha^−1^ (mean ± SD), respectively.

#### Vegetation measurements

**Fig. 13:**
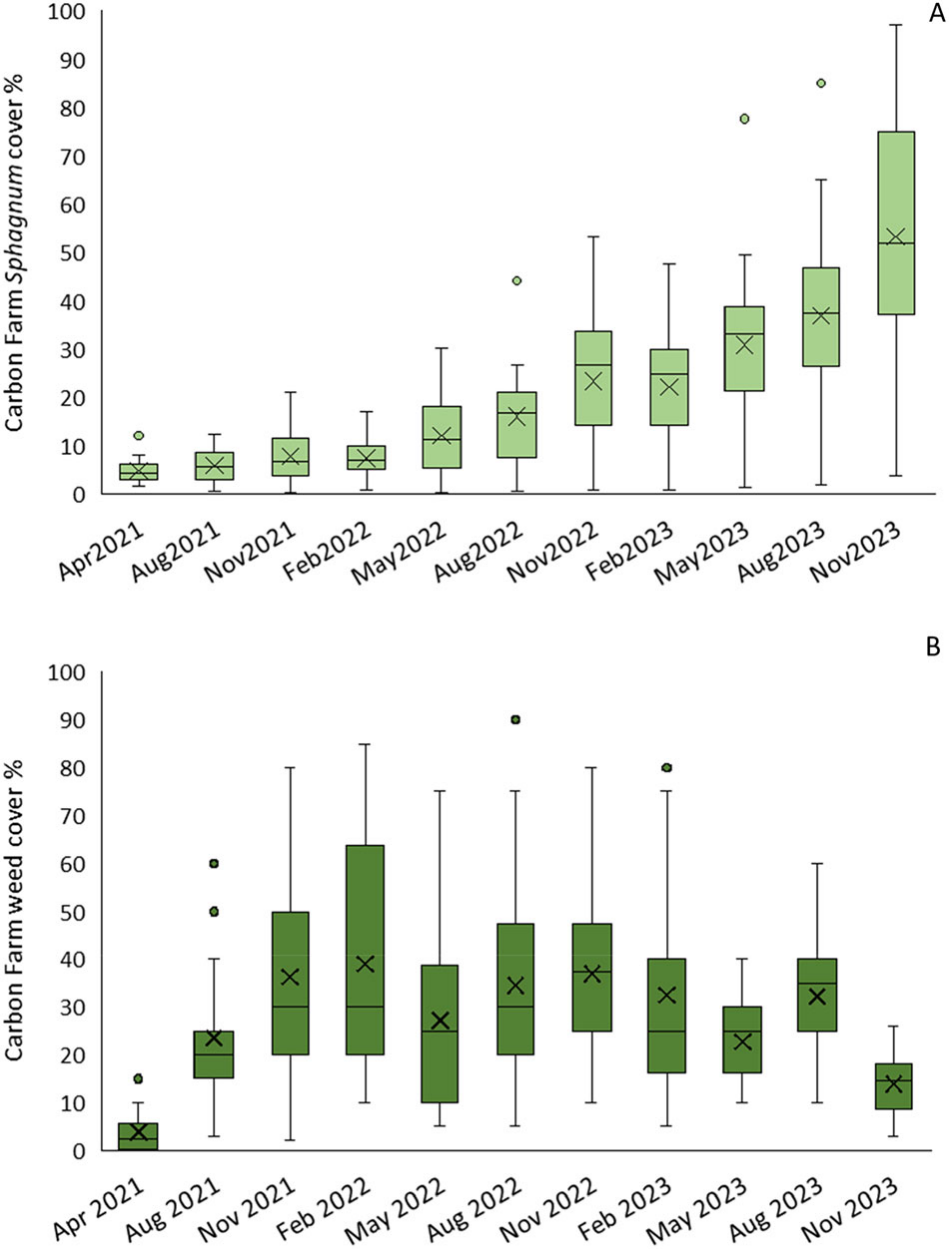
Seasonal measurements of (**A**) *Sphagnum* cover (%) and (**B**) estimates of weed cover (%) using 20 1 × 1 m quadrats across the Carbon Farm; box plots show collated plot data, crosses indicate the mean value, lines indicate the median, and the interquartile range is exclusive

The spread of *Sphagnum* across the whole site was slow over the first two years, partly due to low rainfall coupled with irrigation system technical issues, but increased rapidly in the third year, although with a very large variation in measurements (Fig. 13A). The site has an uneven topography with limited growth in both drier and regularly flooded areas. Weed growth increased rapidly in the first year, but reduced thereafter, particularly in the third year (Fig. 13B). Weeds were a mixture of agricultural grassland forbs and grasses, *Juncus effusus* and *Betula* and *Salix* saplings. Active weed control was limited to regular strimming of *J. effusus*.

**Fig. 14:**
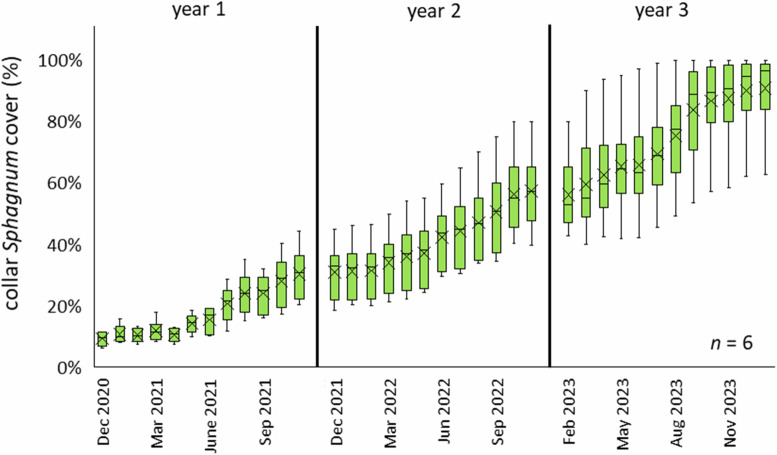
Monthly measured *Sphagnum* cover (%) within CGHG monitoring collars on the Carbon Farm (*n* = 6); box plots show collated plot data, crosses indicate the mean value, lines indicate the median, and the interquartile range is exclusive

**Fig. 15:**
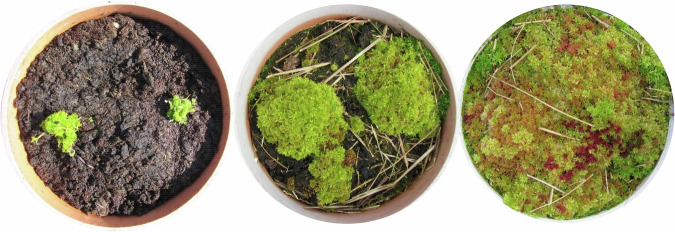
Examples of *Sphagnum* plugs within one collar in September 2020 (at planting), May 2022, and September 2023. A light straw cover was applied post-planting to protect plugs during establishment and reduce water loss through transpiration

*Sphagnum* cover in CGHG collars was 30.3 ± 8.6% (*n* = 6) at the end of year 1 (Fig. 14). Improvements to the irrigation system retained higher peat moisture levels in the second year, which supported continuous *Sphagnum* growth throughout the growing season, giving 57.3 ± 13.3% and 90.78 ± 0.14% cover at the end of years 2 and 3, respectively (*n* = 6). (Fig. 14 and examples in Fig. 15).

#### Measured CGHG Flux

Carbon Farm CO_2_ fluxes were an order of magnitude lower than those on the Grazed Pasture overall. There was greater RECO than NEE overall (apart from year 3 on the Carbon Farm), but the margin of difference between RECO and NEE was greater on the Grazed Pasture and hence led to greater overall emissions.

**Fig. 16:**
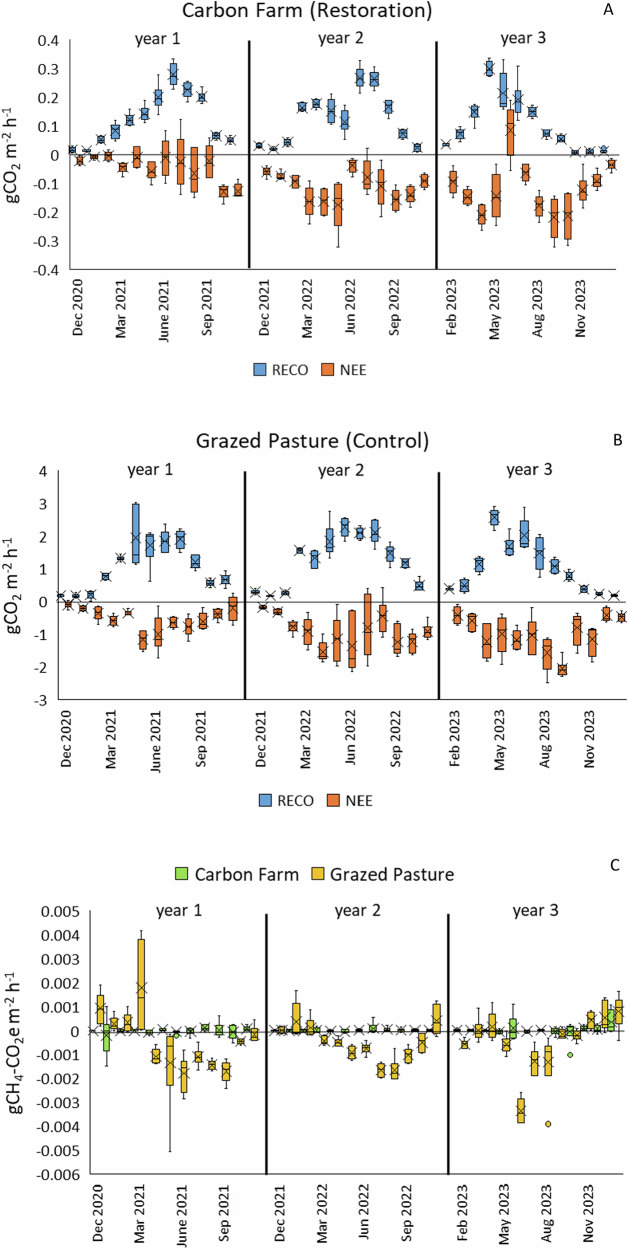
Measured CGHG data on the Carbon Farm pilot in the ‘establishment’ phase (year 1) and post-establishment years (years 2 and 3): **A** RECO and NEE (full light only) on Carbon Farm (Restoration) plots; **B** RECO and NEE (full light only) on Grazed Pasture (Control) plots; **C** methane flux on both treatments, converted to CO_2_ equivalents; box plots show collated plot data, crosses indicate the mean value, lines indicate the median, and interquartile range is exclusive

**Fig. 17:**
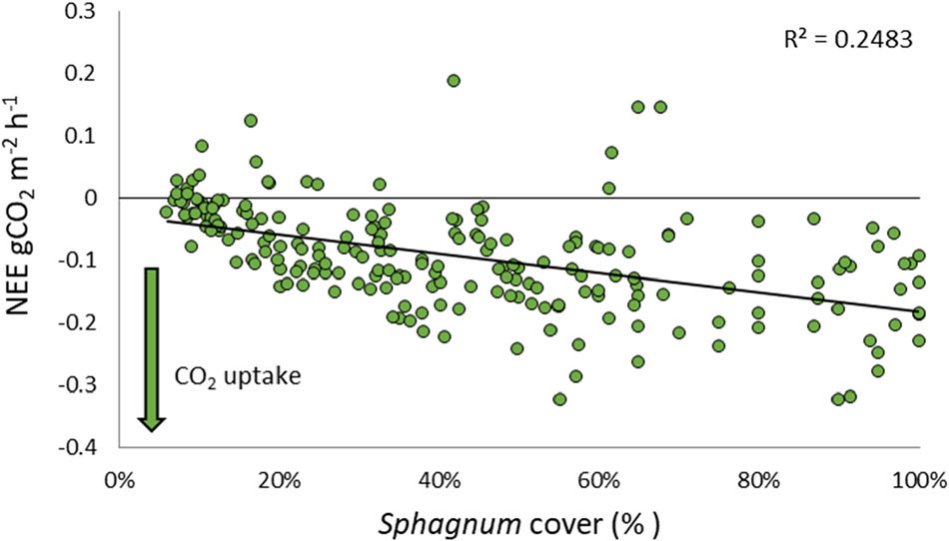
Monthly measured *Sphagnum* cover (%) within CGHG monitoring collars plotted against Net Ecosystem Exchange (full-light) over the 3-year monitoring period, showing increasing CO_2_ uptake with increasing *Sphagnum* cover

**Fig. 18:**
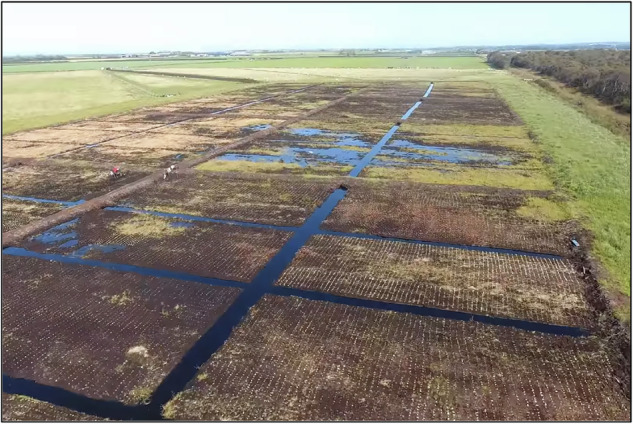
Carbon Farm, Winmarleigh Moss, immediately after *Sphagnum* plug planting (September 2020). Image: M. Longden

**Fig. 19:**
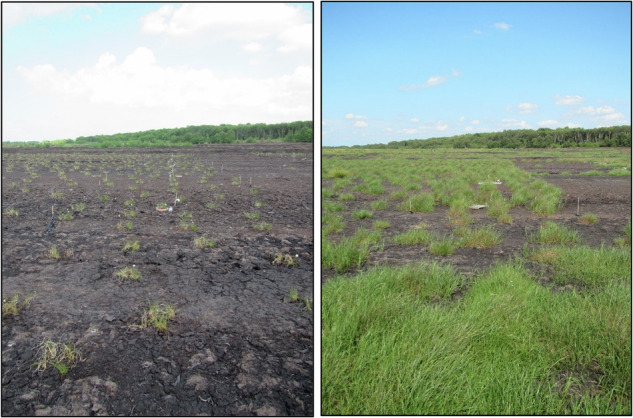
Companion Planting (LWM) GHG monitoring area, May 2020 and July 2021, 2 months and 16 months after planting, respectively

Across all Carbon Farm plots in years 1, 2 and 3 respectively (Fig. 16A) RECO (mean ± SD) was 0.13 ± 0.13, 0.12 ± 0.088 and 0.11 ± 0.095 g CO_2_ m^−2^ h^−1^ (no statistically significant differences) and NEE (in full light, mean ± SD) was −0.037 ± 0.088, −0.11 ± 0.062 and −0.12 ± 0.098 g CO_2_ m^−2^ h^−1^ (statistically significant differences between years 1 and 2, *p* < 0.001, and years 1 and 3, *p* < 0.01 but not years 2 and 3). CO_2_ uptake increased with greater *Sphagnum* cover on the Carbon Farm (*R*^2^ = 0.25, *p* < 0.001) (Fig. 17).

Across Grazed Pasture plots in years 1, 2 and 3 respectively (Fig. 16B) RECO (mean ± SD) was 1.03 ± 0.74, 1.26 ± 0.78 and 1.01 ± 0.77 g CO_2_ m^−2^ h^−1^ and NEE (in full-light, mean ± SD) was −0.53 ± 0.39, −0.90 ± 0.62 and −0.99 ± 0.61 g CO_2_ m^−2^ h^−1^ (no statistically significant differences throughout).

Mean measured CH_4_ fluxes converted to CO_2_ equivalents (Fig. 16C) were very small and highly variable (i.e., negligible) overall: Carbon Farm 1.9^−5^ ± 2.2^−4^, Grazed Pasture: −4.9^−4^ ± 1.1^−3^ (mean ± SD) g CO_2_e m^−2^ h^−1^ across all measurement years. It is assumed that any CH_4_ uptake is due to oxidation to CO_2_, particularly in warm, dry conditions.

#### Measured Flux and environmental variables

Regression models between measured fluxes and environmental variables across the whole monitoring period showed greater RECO fluxes with lowering WTL and particularly increasing TS, and greater GPP with lowering WTL and increasing TS and PAR, which was most evident on Grazed Pasture plots. This partly reflects both the more dynamic nature of the environmental variables and flux measurements on the Grazed Pasture, and the growing season being accompanied by low WTL and high TS for their effect on GPP. The *R*^2^ values for WTL and TS, respectively, against RECO were 0.51 and 0.67 (Carbon Farm) and 0.52 and 0.70 (Grazed Pasture); the *R*^2^ values for WTL, TS and PAR, respectively, against GPP were 0.13, 0.40 and 0.37 (Carbon Farm) and 0.37, 0.60 and 0.55 (Grazed Pasture), with *p* < 0.001 throughout.

#### Modelled CGHG flux budget

RECO plus FCH_4_ (as combined Emission values) was modelled using peat temperature (TS) as the primary driver and WTL as the secondary driver throughout. Primary drivers of GPP fluxes were peat temperature (TS) in year 1 and mostly PAR in subsequent years for both Restoration and Control sites, with secondary and tertiary drivers varying between collars. A breakdown of the drivers with *R*^2^ values of linear regression equations used for modelling is shown in Table 6 (Appendix). Data were highly variable between collars and between monitoring years due to fluctuating environmental conditions and the position of collars in relation to irrigation channels and the sump area (Carbon Farm), and a drainage ditch (Grazed Pasture) (Table 5). The carbon balance figures derived from median measured values (Table 7 [Appendix]) show the same general trajectory and a similar range across the project as the modelled values.

**Table 5:** Annual CGHG balance (t CO_2_e ha^−1^ y^−1^) at the Carbon Farm for each treatment in each monitoring year (sum of hourly modelled values for each year), with associated Carbon losses (t C ha^−1^ y^−1^) (CGHG balance x ^12^/_44_).

	Restoration (Carbon Farm)						Control (Grazed Pasture)					
	Year 1		Year 2		Year 3		Year 1		Year 2		Year 3	
	CGHG	C loss	CGHG	C loss	CGHG	C loss	CGHG	C loss	CGHG	C loss	CGHG	C loss
Combined data	3.40	0.93	3.72	1.01	3.83	1.04	24.40	6.66	47.22	12.88	42.96	11.72
Plot 1	0.96	0.26	4.43	1.21	1.37	0.37	27.72	7.56	40.64	11.08	30.46	8.31
Plot 2	1.96	0.53	2.82	0.77	2.74	0.75	24.42	6.66	62.26	16.98	43.56	11.88
Plot 3	0.23	0.06	3.05	0.83	2.19	0.60	13.23	3.61	34.25	9.34	41.00	11.18
Plot 4	6.59	1.80	5.58	1.52	1.87	0.51	31.78	8.67	38.74	10.57	40.44	11.03
Plot 5	2.95	0.80	5.34	1.46	3.54	0.97	16.50	4.50	33.76	9.21	21.67	5.91
Plot 6	1.71	0.47	1.89	0.52	0.69	0.19	10.90	2.97	38.17	10.41	20.62	5.62
Mean plot data	2.40	0.65	3.85	1.05	2.06	0.56	20.76	5.66	41.30	11.26	32.96	8.99
± SD	2.25	0.61	1.49	0.41	1.01	0.27	8.43	2.30	10.61	2.89	10.19	2.78

Values shown as modelled from collated plot data, and modelled from individual plot data. Values are all positive, i.e., CO_2_e emission

Carbon Farm plot collar to nearest irrigation ditch: 1: 7.5 m, 2: 1.5 m, 3: 4 m, 4: 8.5 m, 5: 1 m, 6: 4 m (4, 5 & 6 in cell closest to water sump); Grazed Pasture plot collar to drainage ditch: 1: 5 m, 2: 1 m, 3: 5 m, 4: 1 m, 5: 5 m, 6: 1 m

High rainfall and low temperatures in year 1 compared to subsequent years led to lower emissions (due to comparatively low RECO) on both Restoration and Control plots compared to year 2, despite low cover of *Sphagnum* on the Carbon Farm. Year 2 was the driest and warmest year and led to the highest emissions over the study period. Despite increasing *Sphagnum* cover the low WTL periods during spring and summer were sufficient to keep RECO levels high and suppress *Sphagnum* photosynthesis on the Carbon Farm, giving sub-optimal GPP levels. High rainfall in year 3 helped to reduce emissions compared to year 2 by lowering RECO. Good, healthy *Sphagnum* cover on the Carbon Farm in year 3, despite occasional irrigation issues, allowed comparatively improved GPP levels and the lowest annual emission over the study period.

### Discussion Section Companion Planting Pilot (Little Woolden Moss)

Evidence from studies on previously exploited peatlands with natural vascular plant colonisation suggests that *Eriophorum*-dominated sites are generally Carbon GHG (CGHG) sinks in the longer term, but fluxes fluctuate between sink and source on restoration sites due to changes in climatic conditions (Wilson et al. 2016). CGHG balances in t CO_2_e ha^−1^ y^−1^ are reported by Beyer and Höper (2015): −2.81 to 6.45 over 2 years, and in each year of measurement, Wilson et al. (2013): −19.76, −3.77 and −9.48, Renou-Wilson et al. (2019): −0.46, 1.74, 13.36 and 10.59, and Keightley et al. (2023): −5.55 and −1.22. In this study, the CGHG balance on *Eriophorum* plots for each year was −22.41, 26.09 and 16.41 t CO_2_e ha^−1^ y^−1^ (individual plot means), which were comparable to values in the literature but generally larger. There are no other studies with results from a newly planted site using micro-propagated plug plants, which were grown under controlled and supportive greenhouse conditions prior to planting out on site. In natural conditions, *E. angustifolium* displays continuous new growth as rhizomes expand outwards from parent plants and produce new shoots, then senescence of original material after flowering, and slower regrowth towards the spaces left by dead plants (Phillips 1954). Younger stands of perennial sedges and grasses typically show faster rates of net photosynthesis than mature plants as the age structure of the canopies progresses (Robertson and Woolhouse 1984; Tejera et al. 2022). We surmise that the rapidly growing new plant material assimilated high levels of CO_2_ but, due to being the same age, there was then generalised senescence and typically slower regrowth, as described above. A more even sward will develop over time and then future variance in carbon GHG fluxes due to climatic variability is likely to follow those of other studies.

There was rapid *Eriophorum* growth in the first year of study across plots, apart from on plot 2, which was on an old peat bed, although growth on plot 5, also an old peat bed, was mid-range. Initial growth was also supported by regular rainfall, with the best growth associated with the highest water table levels (WTL). An adequate WTL also helped to minimize CO_2_ losses (oxidation of carbon) from the peat substrate. Additionally, there was no plant litter (dead plant material) to add to CO_2_ losses through microbial respiration (Liang et al. 2021). Thereafter, periods of drought in the following two years (particularly year 2) are likely to have caused early plant senescence (Bubier et al. 2003), reducing photosynthetic CO_2_ uptake (Kokkonen et al. 2022), and high emission of CO_2_ from the peat substrate through oxidation (Rydin and Jeglum 2013) (measured data: RECO: 0.89 ± 0.63, 0.91 ± 0.79, 0.65 ± 0.54 g CO_2_ m^−2^ h^−1^ years 1, 2 and 3 respectively; GPP −3.15 ± 1.87, −1.50 ± 1.20, −1.32 ± 1.03 g CO_2_ m^−2^ h^−1^ years 1, 2 and 3, respectively [mean ± SD]). This, coupled with the lower CO_2_ uptake necessary to support mature plants (not in a highly-active growth phase), as well as CO_2_ emission from an increasing collection of plant litter, resulted in large overall CO_2_e emissions in years 2 and 3. Other factors were a surprisingly low average PAR during the prolonged summer drought of year 2 (Fig. 6), reducing photosynthesis, and a comparatively higher PAR in spring and early summer of year 3, promoting photosynthesis. The high and persistent rainfall from summer onwards in year 3 raised groundwater levels and reduced CGHG emissions from the peat substrate, and likely supported vascular plant and *Sphagnum* growth, but also reduced PAR through cloud cover, subsequently reducing photosynthesis and CO_2_ uptake.

WTL was not a strong driver of GHG emissions in this pilot (contrary to other research evidence, e.g., Holden 2005; Lazcano et al. 2020; Evans et al. 2021), and RECO was surprisingly similar across study years, but peat moisture is likely to affect the plant quality, either supporting healthy growth or promoting desiccation, thereby influencing the photosynthetic capacity of the plants to take up CO_2_ (Keane et al. 2025). Additionally, although WTL measurements in the Bare (Control) area were slightly higher than the Vegetated (Restoration) area, perhaps due to slightly deeper peat in the Control area, which helped retain higher moisture levels (Moore et al. 2021), or uptake by plants in vegetated areas, it was not sufficient to significantly affect CGHG fluxes.

CGHG fluxes on Bare plots were low throughout (roughly a tenth of those on Vegetated plots) (Fig. 9B) and followed an expected pattern of higher emission in warmer, drier months and lower emission in cooler, wetter months. Over time, there was more difference between NEE and RECO on some plots as acrocarpous mosses developed and photosynthesized on the peat surface. However, the driest plots (e.g., plot 5) and those with poor-quality (hard, humified) peat (e.g., plot 6) continued to emit CGHGs in increasing amounts over the study period (Table 3). Methane fluxes were negligible throughout (Fig. 9C), even on plots with regular inundation (e.g., plot 3). CGHG emission from bare peat (4.3 to 7.5 t CO_2_e ha^−1^ yr^−1^, mean plot data) was higher than on the adjoining Cadishead Moss, where peat is deeper (3.79 t CO_2_e ha^−1^ yr^−1^ from Keightley et al. (2023)), but within the range of UK Tier 2 Emissions factors for Extracted (industrial) bare peat: 6.6 (low 4.8, high 8.1) t CO_2_e ha^−1^ yr^−1^ (Evans et al. 2017).

Annual Carbon losses averaged over the study period were 4.4 ± 2.9 t C ha^−1^ yr^−1^ on the Restoration plots and 1.7 ± 0.5 t C ha^−1^ yr^−1^ on the Control plots (based on mean plot data), which suggests that there may be a complete decomposition of peat stock in 70 years with current Restoration practice (stock = 312 t ha^−1^) and 229 years on bare peat areas (stock = 390 t ha^−1^) unless there is a change in site management (Brouns et al. 2015). Future management needs to involve more reliable water retention in the peat (Price et al. 2003) and the development of an effective acrotelm (Lucchese et al. 2010) through the re-introduction of *Sphagnum* mosses.

Methane emission, although high in the initial stages of plant growth (Fig. 9C), was not sufficient to negate high CO_2_ uptake on vegetated plots in year 1. It was not possible to quantify the influence of each *Eriophorum* species on CGHG fluxes in the first year. When collars were moved to mature stands of *E. angustifolium* after the first year of measurements (due to overcrowding by *E. vaginatum* plants), methane emissions reduced to negligible amounts. It is not clear whether this was due to reduced efficiency of mature plants to convey methane through aerenchymatous tissues, greater oxidation in the rhizosphere, which reduced methane production (Bhullar et al. 2013), or merely the reduced amount of plant material (Fig. 8), both above and below ground. As the volume of plant material increased throughout year 3 and methane emissions did not, the former cases could be assumed, or the larger root system and biomass of *E. vaginatum* in year 1 may have led to higher production rates, but the relationships between *Eriophorum* spp. and biotic and abiotic factors that affect CH_4_ flux are complex (Jordan et al. 2016). Periods of drought could also have reduced methane production in the peat substrate due to oxidation (Boonman et al. 2023). Some precise research to unravel this aspect of restoration practice would be useful.

**Fig. 20:**
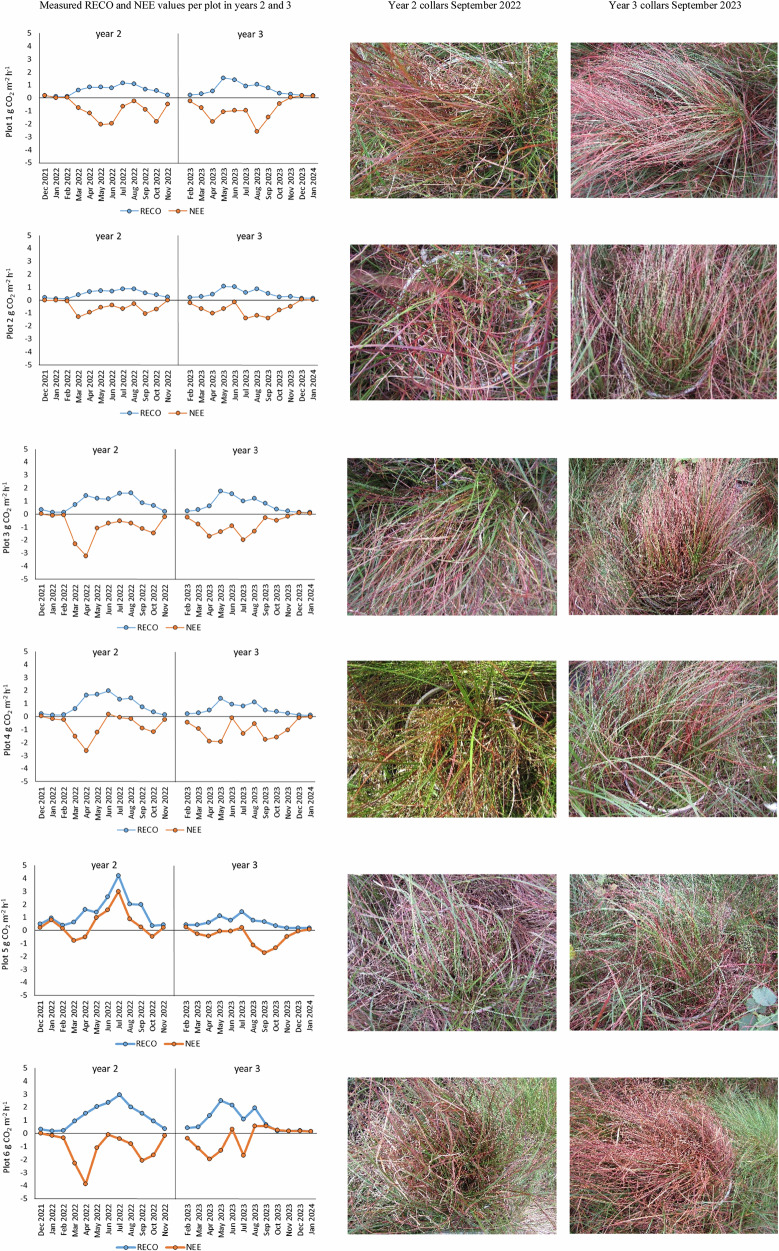
Measured RECO and NEE values from each Companion Planting (LWM) GHG plot for years 2 and 3 with images alongside showing vegetation condition in each year

**Fig. 21:**
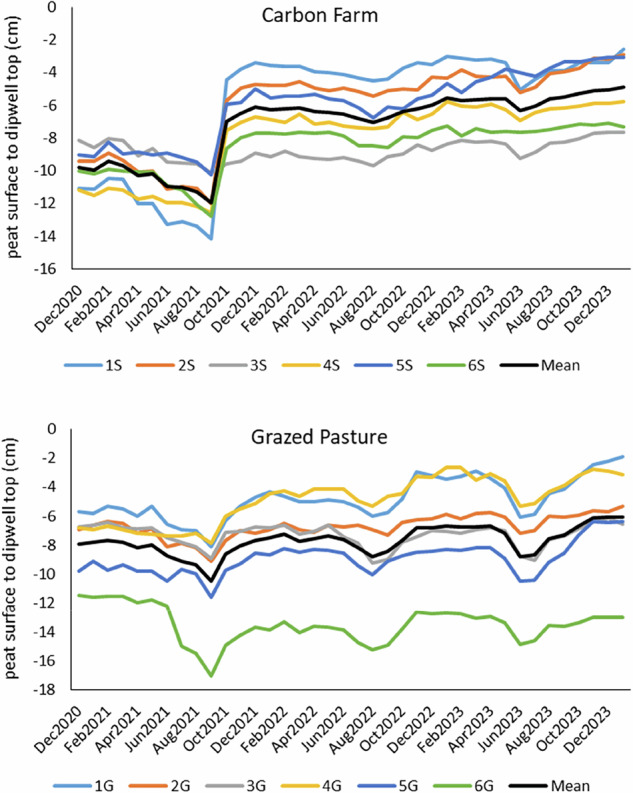
Measurements of the distance between the peat surface and top of dipwells, showing a sudden rise in the Carbon Farm peat surface after irrigation became more effective in late summer 2021, and further gradual rise thereafter, interspersed with drops during dry periods. The peat surface on the drained Grazed Pasture responds to changes in rainfall patterns only

The addition of *Sphagnum* to half of the vegetated plots in year 3, coupled with the recovery of *E. angustifolium* in some plots and collapse in others, made for interesting observations between years 2 and 3 (Fig. 20 [Appendix]), which have the potential to support future restoration decisions based on a carbon balance basis, although broader work in this area is needed to explore these assumptions. Most notable were Plots 4 and 5 due to the improvement in CO_2_ uptake and reduction in RECO in year 3 compared to the previous year, likely resulting from new growth of *E. angustifolium* after senescence/poor growth in year 2, and the addition of *Sphagnum* to plot 4 which probably increased moisture at the peat surface and continued to photosynthesize at lower light levels than the *E. angustifolium* (Keightley et al. 2024). Plot 6 was notable for far lower CO_2_ uptake in year 3 compared to year 2 due to complete senescence of plant material during the summer. All plots had lower RECO in the latter part of year 3 than year 2, likely due to the higher WTL.

These observed differences may highlight the changing nature of *E. angustifolium* as a ground cover plant and thus its variable efficacy for CO_2_ uptake, particularly with varying annual climatic conditions (e.g., Beyer and Höper 2015; Renou-Wilson et al. 2019; Mazzola et al. 2021), although this study was complicated by the need to relocate the collars from combined stands of *E. vaginatum* and *E. angustifolium* to *E. angustifolium* only. While the growth cycle of *E. angustifolium* described above allows space for *Sphagnum* to thrive and come to dominate in due course, it is a long-term strategy towards carbon sequestration and eventual storage. But, although slower, it is more likely to develop into *Sphagnum*-dominated, hydrologically-functioning bog (Telgenkamp et al. 2025) than the more difficult task of keeping bare peat wet enough (but not inundated) for more immediate *Sphagnum* application, particularly where the peat substrate is shallow, so with poorer water-holding capacity (Dixon et al. 2017), and of poor-quality, as on this site. Studies on the neighbouring site of Cadishead Moss (Keightley et al. 2023) demonstrated that, on a site dominated by established *E. angustifolium* vegetation, with introduced *Sphagnum*, more mature plant stands bring greater climate benefits in terms of CGHGs, and are more resilient to climate fluctuations than immature stands, albeit coupled with greater peat depth that supports higher ground-water levels and subsequently plant health (Lindsay and Clough 2016). There is a balanced growth/senescence regime on an established site, unlike on the Care Peat pilot, where there was overcrowding of plants and mass senescence because plants were immature and of the same age.

### Carbon Farm Pilot (Winmarleigh Moss)

The differences in measured CGHG fluxes between Restoration and Control plots were clearly seen, with fluxes on the re-wetted Carbon Farm being a tenth of those on the Grazed Pasture. Both followed expected trends of greater RECO and NEE values in warmer, drier months and reduced values in colder, wetter months. Periods of drought, e.g., spring of year 3, caused a spike in emissions and a reduction in NEE, particularly on the Carbon Farm, where surface desiccation of the *Sphagnum* reduced photosynthetic potential (Bortoluzzi et al. 2006; Helfter et al. 2014; Keane et al. 2025). As inundation on the Carbon Farm was not intended, with the target WTL being 10 cm below the surface (Evans et al. 2021), this could be viewed as conversion from deep to shallow drainage rather than paludiculture. However, although the target WTL was not regularly achieved, transitioning grazed agricultural peat to *Sphagnum*-dominated bog mitigated 88, 91 and 94% of CGHG emissions from the Grazed Pasture in years 1, 2 and 3, respectively (mean plot data).

Methane fluxes were highly variable but low enough throughout to be negligible. The Carbon Farm mean methane flux across all measurement years was 0.02 g CH_4_ m^−2^ yr^−1,^ whereas fluxes in other studies for rewetted *Sphagnum*-dominated bogs were far higher; for example, 31.1 (Beyer and Höper 2015) and 46.8 (Evans et al. 2016) g CH_4_ m^−2^ yr^−1^. There are several possible explanations for this. Perhaps peat oxidation due to a fluctuating water table (Boonman et al. 2023), or denitrification after previous application of agricultural fertilizer, whereby “denitrifiers outcompet(e) methanogens for organic substrate” (Vroom et al. 2018), had reduced methane production. Alternatively, the methanogenic community may not have re-established after a long period of previous peat drainage and oxidation (Juottonen et al. 2012). Also, perhaps oxidation of available CH_4_ to CO_2_ during warmer, drier months by methanotrophs (methane-oxidizing bacteria) (Wang et al. 2017) occurred, shown by an uptake of CH_4_ on the Grazed Pasture. On the Carbon Farm, there may also have been methanotrophic consumption of methane in the *Sphagnum* layer (van Winden et al. 2012; Nugent et al. 2018).

WTL (Fig. 10) was considerably higher throughout on the Carbon Farm compared to the deep-drained Grazed Pasture, despite problems with provision of irrigation water due to occasional pumping failures and sump capacity, demonstrating that cessation of drainage was key to maintaining adequate peat moisture (observed) to support *Sphagnum* health overall. A tested alternative to relying on groundwater is overhead irrigation, which was used in an Innovate UK-funded ‘Sphagnum Farming UK’ project (data unpublished) and promoted a more rapid *Sphagnum* cover (Wright et al. 2021). Increasing *Sphagnum* cover on the Carbon Farm led to a corresponding increase in CO_2_ uptake over the study period (Fig. 17), and although *Sphagnum* cover increased steadily, achieving a full cover in some CGHG monitoring collars towards the end of the 3-year study period (Fig. 14), growth stalled during drier periods when water provision was limited e.g., summer year 1 and spring years 2 and 3 (Fig. 11). Hence, maintaining high peat moisture will promote optimum *Sphagnum* growth (Gaudig et al. 2017) and is likely to result in better carbon outcomes. *Sphagnum* cover measured across the whole site initially lagged behind cover measured in the CGHG collars, probably due to greater weed proliferation on the western half of the pilot, which compromised *Sphagnum* growth, whereas the collars were positioned on the eastern half. Competition from weeds reduced gradually after regular strimming of *Juncus effusus* and allowed an increase in *Sphagnum* growth (Gaudig et al. 2017), which subsequently mostly overcame the growth of other low-growing weed species (van Breemen 1995).

CGHG emissions from the Grazed Pasture (20.8 to 41.3 t CO_2_e ha^−1^ yr^−1^, mean plot data) are similar to, although a little higher overall than, the range of UK Tier 2 Emissions factors for Intensive Grassland: 23.5 (low 13.6, high 33.4) t CO_2_e ha^−1^ yr^-1^ (Evans et al. 2017). The variation each year is likely related to the prolonged summer drought in year 2, which increased soil respiration and reduced plant CO_2_ uptake due to desiccation stress, and a combination of spring drought and prolonged periods of heavy rain in year 3, which increased and then reduced soil respiration, but suppressed plant CO_2_ uptake on the Grazed Pasture due to desiccation and then low light levels under greater cloud cover. This highlights the importance of updating emissions factors to take responses to climate change into account. The cloud cover was of reduced influence on the Carbon Farm as *Sphagnum* photosynthesizes at lower light levels than vascular plants (Laing et al. 2014; Keightley et al. 2024).

Carbon losses averaged over the study period were 0.8 ± 0.3 t C ha^−1^ yr^−1^ on the Carbon Farm plots and 8.6 ± 2.8 t C ha^−1^ yr^−1^ on the Grazed Pasture plots (based on mean plot data), which suggests that there may be a complete decomposition of peat stock in 1,055 years on Carbon Farm areas (stock = 844 t ha^−1^) and only 80 years with current Grazed Pasture practice (stock = 692 t ha^−1^). While there is an expectation that the Carbon Farm will sequester carbon once a complete acrotelm of *Sphagnum* develops, and that peat degradation will cease, current farming practices on the Grazed Pasture are not sustainable (Brouns et al. 2015).

The stripped topsoil was relocated to the adjacent grazed pasture to grass over, as the project was not permitted to dispose of it elsewhere, and it is recognized that, despite already having a reduced carbon content (38.7 ± 5.2% mean ± SD in the top 10 cm), it would be a source of CO_2_ emission. Huth et al. (2022) estimated 1391 t CO_2_e ha^−1^ loss for 60 cm depth of topsoil removal (TSR), and van den Berg et al. (2024) 557 t CO_2_e ha^−1^ loss with 20 cm depth, although these values assume the usual carbon content in the peat, of around 50% or above whereas the peat on the Grazed Pasture was only around 40%. So, for the ~2 ha Carbon Farm with 10 cm TSR of 40% carbon-content peat, there could have been a loss of approximately 408 t CO_2_e through TSR, assuming full mineralisation of the soil carbon. This is unlikely given that it was incorporated into the grazed pasture. In the longer-term, TSR is preferable despite initial CO_2_ losses, as it mitigates ongoing problems of potential high CH_4_ emission (Huth et al. 2022), eutrophication, and high dissolved organic carbon losses (Harpenslager et al. 2015; Zak et al. 2018) from merely rewetting agricultural land, and provides the ideal low-nutrient, acidic surface conditions for *Sphagnum* proliferation to generate the self-managed, eco-hydrological conditions necessary for a functioning bog (Harpenslager et al. 2015; Huth et al. 2022).

A full GHG balance for both treatments, to include aquatic fluxes, emissions from bare-peat bunding, and N_2_O emissions from the fertilized Grazed Pasture, was not possible within the scope of this project. The bunding around and within the Carbon Farm covers approximately 0.16 ha. If emission from bunds is similar to that of industrially extracted peatlands: 6.61 t CO_2_e ha^−1^ yr^−1^ (Evans et al. 2017), then the annual emission from Carbon Farm bunding is in the region of 1.04 t CO_2_e. However, bunding on the Carbon Farm is already becoming colonized by peatland vascular plants, likely to be absorbing CO_2_ through photosynthesis, and will eventually become subsumed by the rising levels of *Sphagnum*-dominated acrotelm within the cells. *Sphagnum* measurements across the Carbon Farm in November 2025 (i.e., 5 years after planting) indicate that overall cover is now 93.8 ± 18.1% to a depth of 12.3 ± 4.2 cm (mean ± SD). Irrigation ditches are already colonized by *S. cuspidatum*, likely to reduce any current methane emission (Parmentier et al. 2011; Kox et al. 2021; Daun et al. 2023), and will also become part of the restored bog over time. N_2_O emissions from the Grazed Pasture (intensive grassland) are likely to account for a further and ongoing 3.08 t CO_2_e ha^−1^ yr^−1^ (Ross 2024). Thus, it is plausible that the Carbon Farm CO_2_e emissions are currently far lower than those on the existing Grazed Pasture, and likely to reduce further over time.

## Conclusions

Restoring peatland carbon storage capacity on these two UK pilots proved challenging and was not achieved in the initial three years. Growing-season drought conditions over the project period hampered progress on both sites, and highlight the urgent need for improving resilience to climate change in peatland restoration projects, particularly on shallow-peat sites. Re-vegetation with *Eriophorum* spp. on a post-extraction site, even on shallow, poor-quality peat, can be achieved quickly, but consistent CGHG benefits are not likely, due to climatic variation and the cyclical nature of *Eriophorum* growth and senescence, until a cover of *Sphagnum* re-establishes, although biodiversity gain will be rapid (Osborne et al. 2021). Conversion of drained, grazed pasture to *Sphagnum*-dominated bog reduced emissions considerably and consistently and appears likely to rapidly deliver good climate-action outcomes.

## Supplementary information

Supplementary information

## Data Availability

The original data presented in the study are openly available in Mendeley Data: Keightley, Anna (2025), “LWM all data”, Mendeley Data, V1, 10.17632/66pmryr74x.1Keightley, Anna (2025), “Winmarleigh All data”, Mendeley Data, V1, 10.17632/fxcfcgcvyn.1Keightley, Anna (2025), “Care-Peat Pilot sites hourly environmental data”, Mendeley Data, V1, 10.17632/t9fcsvwy5r.1.

## Appendix

Figs. 18,19,20,21

Tables 6, 7

**Table 6:** Primary (1), secondary (2), and tertiary (3) drivers of Gross Primary Productivity (GPP) or Net Ecosystem Exchange (NEE) fluxes (as appropriate) on both pilot sites, with *R*^2^ values of linear regression equations used for modelling; PAR = photosynthetically active radiation; TS = peat temperature; WTL = water table level below the surface

LWM		Year 1											
GPP Drivers	1	*R*^*2*^	2	*R*^2^	3	*R*^2^	NEE Drivers	1	*R*^2^	2	*R*^2^	3	*R*^2^
1 V	PAR	0.45	TS	0.39	WTL	0.17	1B	PAR	0.57	TS	0.42	WTL	0.44
2 V	PAR	0.63	TS	0.56	WTL(NM)	0.34	2B	TS*	0.42	PAR	0.52	WTL	0.19
3 V	PAR	0.52	WTL	0.36	TS	0.28	3B	TS	0.58	PAR	0.49	WTL	0.41
4 V	TS	0.35	PAR	0.31	WTL(NM)	0.19	4B	TS	0.60	PAR	0.54	WTL	0.46
5 V	TS	0.39	PAR	0.31	WTL	0.28	5B	TS	0.65	PAR	0.52	WTL	0.29
6 V	TS	0.48	PAR**	0.25	WTL	0.28	6B	TS	0.65	PAR	0.52	WTL	0.47

LWM		Year 2											
1 V	PAR	0.57	TS	0.45	WTL	0.40	1B	WTL	0.81	TS	0.78	PAR	0.28
2 V	PAR*	0.42	TS	0.52	WTL(NM)	0.32	2B	TS	0.46	WTL	0.35	PAR	0.15
3 V	PAR	0.57	TS	0.43	WTL	0.26	3B	TS	0.67	WTL	0.57	PAR	0.20
4 V	PAR	0.74	WTL	0.33	TS	0.24	4B	TS	0.78	WTL	0.67	PAR	0.17
5 V†	WTL	0.42	TS	0.33	PAR	0.01	5B	TS	0.84	WTL	0.74	PAR	0.49
6 V	PAR*	0.46	TS	0.51	WTL	0.40	6B	TS	0.89	WTL	0.75	PAR	0.43

LWM		Year 3											
1 V	PAR	0.73	TS	0.66	WTL	0.20	1B	WTL	0.65	TS	0.47	PAR	0.37
2 V	PAR	0.71	TS	0.63	WTL	0.42	2B	WTL	0.44	TS	0.42	PAR	0.37
3 V	PAR	0.72	WTL	0.62	TS	0.60	3B	TS*	0.17	PAR	0.17	WTL	0.49
4 V	PAR	0.41	TS	0.21	WTL	0.20	4B	WTL	0.75	TS	0.64	PAR(NM)	0.38
5 V	TS	0.41	PAR	0.16	WTL	0.00	5B	TS	0.74	WTL	0.50	PAR	0.12
6 V	WTL	0.61	PAR	0.37	TS	0.18	6B	TS	0.96	WTL	0.57	PAR	0.48

Winmarleigh		Year 1											
GPP Drivers	1	*R*^2^	2	*R*^2^	3	*R*^2^	GPP Drivers	1	*R*^2^	2	*R*^2^	3	*R*^2^
1S	TS	0.60	WTL	0.50	PAR	0.11	1G	TS	0.77	PAR	0.61	WTL	0.60
2S	TS	0.75	WTL	0.56	PAR	0.08	2G	TS	0.88	PAR**	0.56	WTL	0.66
3S	TS	0.89	WTL	0.61	PAR	0.41	3G	TS	0.78	PAR**	0.53	WTL	0.53
4S	TS	0.54	PAR	0.42	WTL	0.22	4G	TS*	0.50	PAR	0.67	WTL	0.33
5S	TS	0.62	PAR**	0.33	WTL	0.34	5G	TS	0.73	PAR	0.63	WTL	0.57
6S	TS	0.77	PAR	0.37	WTL	0.34	6G	TS	0.70	PAR**	0.56	WTL	0.67

Winmarleigh		Year 2											
1S	PAR	0.50	TS**	0.41	WTL	0.43	1G	TS	0.59	PAR**	0.40	WTL(NM)	0.44
2S	PAR*	0.59	WTL	0.64	TS	0.47	2G	PAR	0.73	TS	0.41	WTL	0.35
3S	PAR*	0.61	WTL	0.69	TS	0.22	3G	TS	0.59	WTL	0.48	PAR	0.41
4S	PAR	0.65	WTL	0.53	TS	0.52	4G	PAR*	0.66	TS	0.67	WTL	0.44
5S	PAR	0.60	TS	0.60	WTL	0.46	5G	PAR*	0.59	TS	0.65	WTL	0.49
6S	PAR	0.66	TS	0.41	WTL	0.44	6G	PAR	0.81	TS	0.48	WTL	0.39

Winmarleigh		Year 3											
1S	PAR	0.18	TS	0.09	WTL	0.04	1G	PAR*	0.50	TS	0.62	WTL	0.12
2S	PAR	0.26	TS	0.17	WTL	0.04	2G	PAR	0.74	TS	0.76	WTL	0.26
3S	PAR	0.36	TS	0.22	WTL	0.00	3G	PAR	0.85	TS	0.74	WTL	0.51
4S	TS*	0.03	WTL**	0.09	PAR	0.11	4G	PAR	0.70	TS	0.60	WTL	0.29
5S	PAR*	0.59	WTL	0.62	TS	0.42	5G	PAR*	0.78	TS	0.83	WTL	0.36
6S	PAR	0.59	TS	0.39	WTL	0.31	6G	PAR	0.71	TS	0.64	WTL(NM)	0.36

Treatments: Vegetated (V), Bare (B), Carbon Farm - Sphagnum (S), Grazed Pasture (G). *R*^*2*^= linear regression; *NM* not modelled; * used as primary driver despite lower *R2* value than secondary or tertiary; ** used as secondary driver despite lower *R2* value than tertiary; † NEE drivers (GPP could not be modelled). Outliers removed: 5S, 6S (16/07/2021); 2G, 5G, 6G (19/05/2021)

**Table 7:** CGHG balance on each pilot when calculated using combined median values for measured CO_2_e uptake and emission, 12 hours per day for each flux over each year (t CO_2_e ha^−1^ yr^−1^)

LWM	Restoration (Vegetated)			Control (Bare)		
	Year 1	Year 2	Year 3	Year 1	Year 2	Year 3
Combined data	−35.26	22.85	2.79	5.06	5.56	5.26
Plot 1	−64.66	11.37	−0.39	5.56	9.97	5.98
Plot 2	−11.67	4.34	−11.13	3.73	3.71	0.22
Plot 3	−38.29	8.40	−1.36	4.77	9.45	8.32
Plot 4	−60.73	22.98	−18.38	6.49	4.23	4.00
Plot 5	−65.11	69.57	19.12	8.45	10.82	8.01
Plot 6	−21.08	34.06	33.39	4.81	4.14	4.29
Mean plot data	−43.59	25.12	3.54	5.63	7.05	5.14

Winmarleigh	Restoration (Carbon Farm)			Control (Grazed Pasture)		
	Year 1	Year 2	Year 3	Year 1	Year 2	Year 3
Combined data	3.28	1.52	−1.41	24.30	33.51	7.78
Plot 1	2.34	1.04	−2.35	24.90	36.52	8.64
Plot 2	2.89	0.49	−2.85	35.51	47.24	19.32
Plot 3	1.13	−0.06	−0.26	19.89	34.23	1.41
Plot 4	6.09	4.46	−0.43	37.99	21.94	21.29
Plot 5	4.44	3.89	−1.13	14.62	29.12	-3.87
Plot 6	2.82	−0.02	−2.44	7.73	33.07	-6.61
Mean plot data	3.28	1.63	−1.58	23.44	33.69	6.70

Values shown as estimated from collated plot data and from individual plot data. Negative values are CO_2_e uptake and positive values are CO_2_e emission; *Sphagnum* material added to LWM plots 1, 2 and 4 in year 3
