# Supplementary material for: Carbon Fluxes of Contrasting Degraded Peatland Pilot Sites During Early-stage Restoration: Ex-milled Bare Peat and Grazed Grassland Conversion
Source: Environ Manage. 2026 Apr 11;76(5):147. doi: 10.1007/s00267-026-02445-w (PMC13070053; doi:10.1007/s00267-026-02445-w)
Supplement: Supplementary file 1 — Supplementary information [file 267_2026_2445_MOESM1_ESM.docx]

Supplementary Information

**Table S1** All measured data on both pilot sites over the study period. Plots - Little Woolden Moss Companion Planting: V = Vegetated, B = Bare; Winmarleigh Carbon Farm: S = Carbon Farm, G = Grazed Pasture; RECO. GPP, NEE values: g CO_2_ m-^2^ h-^1^; FCH4-CO_2_e = FCH4 values x GWP_28_; RECO and FCH4 measured with dark cloth over chamber, +Mesh = with mesh cover to simulate lower light levels, otherwise measured in full light

| Date | Plot | RECO | NEE (+Mesh) | GPP (+Mesh) | NEE | GPP | FCH4-CO_2_e | Plot | RECO | NEE (+Mesh) | GPP (+Mesh) | NEE | GPP | FCH4-CO_2_e |
| --- | --- | --- | --- | --- | --- | --- | --- | --- | --- | --- | --- | --- | --- | --- |
| 26/06/2020 | 1V | 1.0112 | -1.5782 | -2.5894 | -1.9128 | -2.9241 | 0.1687 | 1B | 0.1484 | 0.1384 | -0.0100 | 0.2108 | 0.0000 | 0.0000 |
| 26/06/2020 | 2V | 1.0346 | -0.7126 | -1.7472 | -0.6497 | -1.6843 | 0.4958 | 2B | 0.2954 | 0.3322 | 0.0000 | 0.3663 | 0.0000 | 0.0001 |
| 26/06/2020 | 3V | 1.1330 | -1.0703 | -2.2033 | -0.7654 | -1.8983 | 0.0657 | 3B | 0.2632 | 0.2392 | -0.0240 | 0.2495 | -0.0137 | -0.0002 |
| 26/06/2020 | 4V | 1.1037 | -1.2803 | -2.3840 | -1.0383 | -2.1420 | 0.1374 | 4B | 0.2755 | 0.2835 | 0.0000 | 0.3145 | 0.0000 | 0.0020 |
| 26/06/2020 | 5V | 1.1161 | -1.4323 | -2.5484 | -1.2798 | -2.3959 | 0.2080 | 5B | 0.3864 | 0.3689 | -0.0175 | 0.3895 | 0.0000 | -0.0001 |
| 26/06/2020 | 6V | 1.4128 | -1.9478 | -3.3607 | -1.8792 | -3.2921 | 0.2714 | 6B | 0.1844 | 0.1660 | -0.0185 | 0.1850 | 0.0000 | 0.0004 |
| 29/07/2020 | 1V | 1.0243 | -3.3254 | -4.3498 | -4.1456 | -5.1699 | 0.3183 | 1B | 0.0649 | 0.0442 | -0.0207 | 0.0383 | -0.0266 | 0.0000 |
| 29/07/2020 | 2V | 0.6479 | -2.1223 | -2.7702 | -2.6220 | -3.2699 | 0.6667 | 2B | 0.0393 | 0.0443 | 0.0000 | 0.0473 | 0.0000 | 0.0000 |
| 29/07/2020 | 3V | 0.4838 | -1.9523 | -2.4361 | -2.0546 | -2.5383 | 0.1420 | 3B | 0.0742 | 0.0516 | -0.0227 | 0.0511 | -0.0232 | 0.0001 |
| 29/07/2020 | 4V | 0.5671 | -2.8632 | -3.4303 | -3.3915 | -3.9586 | 0.3522 | 4B | 0.0815 | 0.0789 | -0.0025 | 0.0788 | -0.0027 | 0.0001 |
| 29/07/2020 | 5V | 0.6419 | -3.1671 | -3.8090 | -3.6973 | -4.3392 | 0.1978 | 5B | 0.1029 | 0.0903 | -0.0126 | 0.0897 | -0.0131 | 0.0000 |
| 29/07/2020 | 6V | 1.1535 | -4.1894 | -5.3429 | -5.0126 | -6.1661 | 0.4272 | 6B | 0.0634 | 0.0540 | -0.0095 | 0.0527 | -0.0108 | 0.0003 |
| 20/08/2020 | 1V | 1.0097 | -3.4123 | -4.4220 | -3.7823 | -4.7920 | 0.4024 | 1B | 0.1080 | 0.1004 | -0.0077 | 0.1241 | 0.0000 | 0.0000 |
| 20/08/2020 | 2V | 0.4502 | -1.9422 | -2.3924 | -1.8021 | -2.2523 | 0.6490 | 2B | 0.0976 | 0.0903 | -0.0073 | 0.0941 | -0.0036 | -0.0001 |
| 20/08/2020 | 3V | 0.5568 | -2.0136 | -2.5704 | -1.2772 | -1.8340 | 0.2154 | 3B | 0.1341 | 0.1439 | 0.0000 | 0.1518 | 0.0000 | -0.0001 |
| 20/08/2020 | 4V | 0.6816 | -3.2768 | -3.9585 | -3.5384 | -4.2200 | 0.3574 | 4B | 0.1046 | 0.0942 | -0.0103 | 0.1098 | 0.0000 | 0.0001 |
| 20/08/2020 | 5V | 0.8609 | -2.6782 | -3.5391 | -3.4579 | -4.3189 | 0.3089 | 5B | 0.2257 | 0.1835 | -0.0422 | 0.1771 | -0.0486 | 0.0001 |
| 20/08/2020 | 6V | 1.2674 | -2.4783 | -3.7457 | -3.1518 | -4.4192 | 0.3550 | 6B | 0.0921 | 0.0701 | -0.0220 | 0.0814 | -0.0106 | 0.0002 |
| 17/09/2020 | 1V | 1.2638 | -3.4038 | -4.6676 | -3.4489 | -4.7127 | 0.2969 | 1B | 0.0763 | 0.0418 | -0.0344 | 0.0497 | -0.0266 | -0.0001 |
| 17/09/2020 | 2V | 0.5769 | -1.6084 | -2.1853 | -1.7583 | -2.3351 | 0.5022 | 2B | 0.0308 | 0.0419 | 0.0000 | 0.0505 | 0.0000 | 0.0000 |
| 17/09/2020 | 3V | 0.7460 | -2.2141 | -2.9601 | -2.2779 | -3.0239 | 0.2173 | 3B | 0.1341 | 0.1355 | 0.0000 | 0.1656 | 0.0000 | 0.0007 |
| 17/09/2020 | 4V | 1.1052 | -3.5337 | -4.6389 | -3.6706 | -4.7758 | 0.3785 | 4B | 0.0828 | 0.0907 | 0.0000 | 0.0971 | 0.0000 | 0.0014 |
| 17/09/2020 | 5V | 1.0151 | -3.0696 | -4.0847 | -3.3129 | -4.3281 | 0.2729 | 5B | 0.1342 | 0.1310 | -0.0032 | 0.1404 | 0.0000 | 0.0001 |
| 17/09/2020 | 6V | 1.4367 | -3.2704 | -4.7071 | -3.5048 | -4.9414 | 0.4060 | 6B | 0.0769 | 0.0560 | -0.0210 | 0.0648 | -0.0121 | 0.0010 |
| 14/10/2020 | 1V | 1.1453 | -4.4673 | -5.6126 | -4.6848 | -5.8301 | 0.3501 | 1B | 0.0493 | 0.0172 | -0.0320 | 0.0207 | -0.0286 | 0.0000 |
| 14/10/2020 | 2V | 0.3972 | -1.6658 | -2.0630 | -1.3338 | -1.7310 | 0.6393 | 2B | 0.0398 | 0.0279 | -0.0119 | 0.0263 | -0.0136 | 0.0000 |
| 14/10/2020 | 3V | 0.3989 | -2.6379 | -3.0367 | -2.6677 | -3.0666 | 0.2440 | 3B | 0.0575 | 0.0342 | -0.0233 | 0.0341 | -0.0235 | 0.0014 |
| 14/10/2020 | 4V | 0.7776 | -1.0329 | -1.8104 | -3.7320 | -4.5096 | 0.4643 | 4B | 0.0301 | 0.0265 | -0.0036 | 0.0224 | -0.0077 | 0.0001 |
| 14/10/2020 | 5V | 0.7668 | -4.0161 | -4.7830 | -4.1741 | -4.9410 | 0.3001 | 5B | 0.0660 | 0.0367 | -0.0293 | 0.0547 | -0.0113 | 0.0006 |
| 14/10/2020 | 6V | 1.0326 | -3.8989 | -4.9315 | -4.2329 | -5.2655 | 0.4363 | 6B | 0.0341 | 0.0220 | -0.0121 | 0.0146 | -0.0196 | 0.0000 |
| 13/11/2020 | 1V | 0.6079 | -2.4943 | -3.1022 | -2.7193 | -3.3272 | 0.2410 | 1B | 0.0278 | 0.0126 | -0.0152 | 0.0151 | -0.0127 | 0.0000 |
| 13/11/2020 | 2V | 0.2390 | -0.9344 | -1.1735 | -1.0034 | -1.2424 | 0.5582 | 2B | 0.0143 | 0.0003 | -0.0141 | 0.0036 | -0.0107 | 0.0000 |
| 13/11/2020 | 3V | 0.2784 | -1.5660 | -1.8444 | -1.7266 | -2.0050 | 0.1969 | 3B | 0.0165 | -0.0110 | -0.0275 | -0.0098 | -0.0263 | 0.0000 |
| 13/11/2020 | 4V | 0.5137 | -2.8975 | -3.4112 | -3.0489 | -3.5626 | 0.4101 | 4B | 0.0138 | 0.0147 | 0.0000 | 0.0076 | 0.0000 | 0.0001 |
| 13/11/2020 | 5V | 0.3802 | -2.4886 | -2.8687 | -2.8243 | -3.2045 | 0.2673 | 5B | 0.0333 | 0.0205 | -0.0128 | 0.0149 | -0.0183 | 0.0002 |
| 13/11/2020 | 6V | 0.6915 | -2.7537 | -3.4452 | -3.0451 | -3.7366 | 0.3569 | 6B | 0.0067 | 0.0027 | -0.0040 | 0.0051 | -0.0016 | 0.0000 |
| 15/12/2020 | 1V | 0.3276 | -0.7999 | -1.1275 | -1.1128 | -1.4404 | 0.1820 | 1B | 0.0066 | -0.0101 | -0.0167 | -0.0117 | -0.0183 | 0.0000 |
| 15/12/2020 | 2V | 0.1746 | -0.4296 | -0.6042 | -0.4440 | -0.6186 | 0.4379 | 2B | 0.0293 | 0.0152 | -0.0140 | 0.0128 | -0.0165 | 0.0002 |
| 15/12/2020 | 3V | 0.1611 | -0.7594 | -0.9205 | -0.8287 | -0.9898 | 0.1558 | 3B | 0.0001 | 0.0059 | 0.0000 | 0.0102 | 0.0000 | 0.0002 |
| 15/12/2020 | 4V | 0.2989 | -1.4489 | -1.7478 | -1.5876 | -1.8865 | 0.2895 | 4B | 0.0074 | 0.0035 | -0.0039 | 0.0043 | -0.0031 | 0.0000 |
| 15/12/2020 | 5V | 0.3000 | -0.9869 | -1.2868 | -1.0857 | -1.3856 | 0.2413 | 5B | 0.0473 | 0.0273 | -0.0200 | 0.0226 | -0.0247 | 0.0003 |
| 15/12/2020 | 6V | 0.3502 | -1.1684 | -1.5186 | -1.2627 | -1.6129 | 0.2247 | 6B | 0.0025 | 0.0095 | 0.0000 | 0.0066 | 0.0000 | 0.0000 |
| 12/01/2021 | 1V | 0.2300 | -0.2799 | -0.5099 | -0.3324 | -0.5624 | 0.1532 | 1B | 0.0089 | -0.0008 | -0.0097 | -0.0027 | -0.0116 | 0.0000 |
| 12/01/2021 | 2V | 0.1803 | -0.2863 | -0.4665 | -0.3143 | -0.4946 | 0.3646 | 2B | 0.0184 | 0.0090 | -0.0093 | 0.0098 | -0.0086 | 0.0001 |
| 12/01/2021 | 3V | 0.1253 | -0.3365 | -0.4618 | -0.4200 | -0.5454 | 0.1327 | 3B | 0.0188 | 0.0168 | -0.0020 | 0.0177 | -0.0011 | 0.0005 |
| 12/01/2021 | 4V | 0.2136 | -0.4724 | -0.6860 | -0.5282 | -0.7418 | 0.2773 | 4B | 0.0055 | 0.0008 | -0.0047 | 0.0004 | -0.0052 | 0.0000 |
| 12/01/2021 | 5V | 0.2247 | -0.3470 | -0.5717 | -0.3968 | -0.6215 | 0.1984 | 5B | 0.0243 | 0.0041 | -0.0202 | 0.0007 | -0.0236 | 0.0002 |
| 12/01/2021 | 6V | 0.3033 | -0.4852 | -0.7885 | -0.5974 | -0.9007 | 0.2063 | 6B | 0.0035 | -0.0007 | -0.0043 | -0.0008 | -0.0043 | 0.0000 |
| 11/02/2021 | 1V | 0.1058 | -0.2878 | -0.3936 | -0.3538 | -0.4595 | 0.1313 | 1B | 0.0327 | 0.0403 | 0.0000 | 0.0614 | 0.0000 | -0.0001 |
| 11/02/2021 | 2V | 0.0787 | -0.2599 | -0.3386 | -0.1958 | -0.2744 | 0.2740 | 2B | 0.0132 | 0.0095 | -0.0037 | 0.0122 | -0.0010 | 0.0004 |
| 11/02/2021 | 3V | 0.0678 | -0.3474 | -0.4152 | -0.4122 | -0.4800 | 0.1183 | 3B | 0.0030 | 0.0011 | -0.0020 | 0.0019 | -0.0011 | 0.0003 |
| 11/02/2021 | 4V | 0.0978 | -0.3558 | -0.4536 | -0.3966 | -0.4945 | 0.2345 | 4B | 0.0240 | 0.0184 | -0.0056 | 0.0221 | -0.0020 | 0.0024 |
| 11/02/2021 | 5V | 0.1204 | -0.3145 | -0.4349 | -0.3312 | -0.4516 | 0.1427 | 5B | 0.0393 | 0.0319 | -0.0074 | 0.0349 | -0.0044 | 0.0009 |
| 11/02/2021 | 6V | 0.1693 | -0.4633 | -0.6325 | -0.4783 | -0.6476 | 0.1667 | 6B | 0.0085 | 0.0070 | -0.0015 | 0.0094 | 0.0000 | 0.0003 |
| 31/03/2021 | 1V | 1.3903 | -1.0390 | -2.4293 | -1.4914 | -2.8817 | 0.1261 | 1B | 0.1098 | 0.0750 | -0.0348 | 0.0636 | -0.0462 | 0.0000 |
| 31/03/2021 | 2V | 0.7185 | -0.6019 | -1.3204 | -1.0043 | -1.7228 | 0.2796 | 2B | 0.0681 | 0.0421 | -0.0260 | 0.0403 | -0.0278 | 0.0010 |
| 31/03/2021 | 3V | 1.0043 | -1.0790 | -2.0833 | -1.5459 | -2.5502 | 0.1248 | 3B | 0.0612 | 0.0384 | -0.0228 | 0.0347 | -0.0265 | 0.0000 |
| 31/03/2021 | 4V | 1.6117 | -1.0824 | -2.6940 | -1.2537 | -2.8654 | 0.2202 | 4B | 0.0501 | 0.0349 | -0.0152 | 0.0357 | -0.0144 | 0.0032 |
| 31/03/2021 | 5V | 1.1958 | -0.8128 | -2.0086 | -1.2014 | -2.3971 | 0.1440 | 5B | 0.1567 | 0.0848 | -0.0719 | 0.0713 | -0.0854 | 0.0004 |
| 31/03/2021 | 6V | 1.7785 | -0.8293 | -2.6077 | -1.1991 | -2.9775 | 0.1555 | 6B | 0.0782 | 0.0625 | -0.0158 | 0.0448 | -0.0334 | 0.0004 |
| 26/04/2021 | 1V | 1.1943 | -2.3635 | -3.5579 | -3.3471 | -4.5415 | 0.1033 | 1B | 0.1477 | 0.1506 | 0.0000 | 0.1427 | -0.0050 | 0.0000 |
| 26/04/2021 | 2V | 0.6316 | -1.1718 | -1.8034 | -1.4341 | -2.0657 | 0.3134 | 2B | 0.0464 | 0.0530 | 0.0000 | 0.0587 | 0.0000 | 0.0008 |
| 26/04/2021 | 3V | 1.0248 | -1.9548 | -2.9795 | -2.4918 | -3.5166 | 0.1346 | 3B | 0.0960 | 0.1067 | 0.0000 | 0.1028 | 0.0000 | 0.0010 |
| 26/04/2021 | 4V | 1.3490 | -2.6885 | -4.0376 | -2.5669 | -3.9159 | 0.1652 | 4B | 0.0724 | 0.0819 | 0.0000 | 0.0928 | 0.0000 | 0.0011 |
| 26/04/2021 | 5V | 1.1057 | -2.0498 | -3.1555 | -2.5395 | -3.6452 | 0.1438 | 5B | 0.0918 | 0.1098 | 0.0000 | 0.1227 | 0.0000 | 0.0004 |
| 26/04/2021 | 6V | 1.2891 | -2.0023 | -3.2914 | -2.1215 | -3.4106 | 0.1047 | 6B | 0.0554 | 0.0657 | 0.0000 | 0.0751 | 0.0000 | 0.0008 |
| 18/05/2021 | 1V | 2.5846 | -3.8923 | -6.4769 | -5.0482 | -7.6328 | 0.1317 | 1B | 0.1373 | 0.0951 | -0.0422 | 0.1297 | -0.0075 | -0.0001 |
| 18/05/2021 | 2V | 0.9518 | -1.9855 | -2.9374 | -2.0797 | -3.0315 | 0.4323 | 2B | 0.0985 | 0.0860 | -0.0125 | 0.0865 | -0.0120 | 0.0002 |
| 18/05/2021 | 3V | 1.5818 | -3.1605 | -4.7423 | -3.8000 | -5.3818 | 0.1573 | 3B | 0.0711 | 0.0389 | -0.0322 | 0.0556 | -0.0155 | 0.0001 |
| 18/05/2021 | 4V | 2.4874 | -4.3571 | -6.8444 | -4.4324 | -6.9198 | 0.2015 | 4B | 0.0834 | 0.0768 | -0.0067 | 0.0724 | -0.0110 | 0.0001 |
| 18/05/2021 | 5V | 1.4523 | -3.5455 | -4.9979 | -4.0158 | -5.4681 | 0.1085 | 5B | 0.1451 | 0.1224 | -0.0227 | 0.1004 | -0.0447 | 0.0008 |
| 18/05/2021 | 6V | 2.1349 | -0.6878 | -2.8226 | -3.1835 | -5.3184 | 0.1580 | 6B | 0.0786 | 0.0712 | -0.0074 | 0.0659 | -0.0127 | 0.0002 |
| 12/06/2021 | 1V | 2.4648 | -2.9017 | -5.3665 | -2.7359 | -5.2007 | 0.1053 | 1B | 0.1533 | 0.1257 | -0.0276 | 0.1011 | -0.0522 | 0.0045 |
| 12/06/2021 | 2V | 0.9268 | -1.7877 | -2.7145 | -1.7770 | -2.7038 | 0.3034 | 2B | 0.0326 | 0.0427 | 0.0000 | 0.0497 | 0.0000 | 0.0001 |
| 12/06/2021 | 3V | 1.6287 | -3.2558 | -4.8845 | -3.7947 | -5.4234 | 0.1133 | 3B | 0.0612 | 0.0602 | -0.0010 | 0.0776 | 0.0000 | 0.0005 |
| 12/06/2021 | 4V | 2.2147 | -3.6408 | -5.8556 | -4.0174 | -6.2321 | 0.1155 | 4B | 0.0844 | 0.0891 | 0.0000 | 0.1133 | 0.0000 | 0.0005 |
| 12/06/2021 | 5V | 1.3610 | -3.7802 | -5.1412 | -3.6780 | -5.0390 | 0.0506 | 5B | 0.1159 | 0.1185 | 0.0000 | 0.1588 | 0.0000 | 0.0010 |
| 12/06/2021 | 6V | 2.4663 | -3.5626 | -6.0289 | -5.4014 | -7.8676 | 0.0919 | 6B | 0.0557 | 0.0700 | 0.0000 | 0.0664 | 0.0000 | 0.0003 |
| 15/12/2021 | 1V | 0.1796 | 0.2177 | 0.0000 | 0.1873 | 0.0000 | 0.0347 | 1B | 0.0090 | -0.0050 | -0.0140 | 0.0128 | 0.0000 | 0.0000 |
| 15/12/2021 | 2V | 0.1821 | 0.0203 | -0.1618 | -0.0139 | -0.1961 | 0.0332 | 2B | 0.0344 | 0.0067 | -0.0277 | 0.0121 | -0.0223 | 0.0001 |
| 15/12/2021 | 3V | 0.3504 | -0.0273 | -0.3777 | 0.0158 | -0.3346 | 0.0324 | 3B | 0.0101 | 0.0193 | 0.0000 | 0.0294 | 0.0000 | 0.0011 |
| 15/12/2021 | 4V | 0.2079 | 0.0547 | -0.1532 | 0.0186 | -0.1893 | 0.0399 | 4B | 0.0234 | 0.0214 | -0.0020 | 0.0225 | -0.0009 | 0.0000 |
| 15/12/2021 | 5V | 0.4745 | 0.2146 | -0.2599 | 0.2109 | -0.2636 | 0.0200 | 5B | 0.0713 | 0.0697 | -0.0015 | 0.0504 | -0.0209 | 0.0000 |
| 15/12/2021 | 6V | 0.3105 | -0.1630 | -0.4736 | 0.0175 | -0.2930 | 0.0312 | 6B | 0.0066 | -0.0100 | -0.0166 | -0.0090 | -0.0156 | 0.0000 |
| 26/01/2022 | 1V | 0.1091 | 0.0585 | -0.0506 | 0.0176 | -0.0915 | 0.0263 | 1B | 0.0349 | 0.0365 | 0.0000 | 0.0287 | -0.0062 | -0.0001 |
| 26/01/2022 | 2V | 0.0991 | 0.0324 | -0.0667 | -0.0274 | -0.1265 | 0.0243 | 2B | 0.0239 | 0.0128 | -0.0111 | 0.0077 | -0.0163 | 0.0000 |
| 26/01/2022 | 3V | 0.1280 | -0.0076 | -0.1356 | -0.1245 | -0.2525 | 0.0324 | 3B | 0.0237 | -0.0528 | -0.0765 | -0.0735 | -0.0972 | 0.0000 |
| 26/01/2022 | 4V | 0.1047 | -0.0596 | -0.1643 | -0.1879 | -0.2926 | 0.0347 | 4B | 0.0097 | 0.0093 | -0.0004 | 0.0075 | -0.0022 | 0.0000 |
| 26/01/2022 | 5V | 0.9291 | 0.7057 | -0.2234 | 0.8183 | -0.1108 | 0.0212 | 5B | 0.0390 | 0.0352 | -0.0038 | 0.0343 | -0.0047 | -0.0001 |
| 26/01/2022 | 6V | 0.1778 | -0.0727 | -0.2505 | -0.1679 | -0.3456 | 0.0462 | 6B | 0.0169 | 0.0059 | -0.0109 | 0.0013 | -0.0156 | 0.0000 |
| 23/02/2022 | 1V | 0.0969 | 0.0850 | -0.0119 | 0.0558 | -0.0411 | 0.0187 | 1B | 0.0088 | 0.0029 | -0.0059 | 0.0038 | -0.0050 | 0.0000 |
| 23/02/2022 | 2V | 0.0986 | -0.0090 | -0.1076 | -0.0710 | -0.1696 | 0.0161 | 2B | 0.0218 | -0.0027 | -0.0245 | -0.0065 | -0.0283 | 0.0001 |
| 23/02/2022 | 3V | 0.1304 | 0.0392 | -0.0912 | -0.0745 | -0.2049 | 0.0251 | 3B | 0.0003 | 0.0010 | 0.0000 | 0.0017 | 0.0000 | 0.0002 |
| 23/02/2022 | 4V | 0.1344 | -0.0711 | -0.2055 | -0.2589 | -0.3933 | 0.0314 | 4B | 0.0337 | 0.0303 | -0.0034 | 0.0349 | 0.0000 | 0.0001 |
| 23/02/2022 | 5V | 0.3817 | 0.0750 | -0.3067 | 0.1331 | -0.2486 | 0.0146 | 5B | 0.0381 | 0.0251 | -0.0130 | 0.0187 | -0.0195 | -0.0001 |
| 23/02/2022 | 6V | 0.1992 | -0.0535 | -0.2527 | -0.3515 | -0.5506 | 0.0287 | 6B | 0.0036 | -0.0237 | -0.0274 | -0.0161 | -0.0197 | 0.0000 |
| 15/04/2022 | 1V | 0.6039 | -0.1259 | -0.7299 | -0.7571 | -1.3610 | 0.0285 | 1B | 0.0729 | 0.1165 | 0.0000 | 0.1163 | 0.0000 | -0.0001 |
| 15/04/2022 | 2V | 0.3899 | -0.9580 | -1.3480 | -1.3114 | -1.7013 | 0.0194 | 2B | 0.0688 | 0.0220 | -0.0468 | 0.0352 | -0.0336 | 0.0001 |
| 15/04/2022 | 3V | 0.7037 | -0.5002 | -1.2039 | -2.2879 | -2.9916 | 0.0278 | 3B | 0.1596 | 0.0046 | -0.1550 | -0.0444 | -0.2040 | -0.0001 |
| 15/04/2022 | 4V | 0.5977 | -1.4330 | -2.0308 | -1.5121 | -2.1098 | 0.0246 | 4B | 0.0398 | 0.0391 | -0.0007 | 0.0395 | -0.0003 | 0.0001 |
| 15/04/2022 | 5V | 0.6274 | -0.1979 | -0.8253 | -0.8045 | -1.4319 | 0.0093 | 5B | 0.0716 | 0.0674 | -0.0042 | 0.0667 | -0.0050 | 0.0000 |
| 15/04/2022 | 6V | 0.9319 | -1.0036 | -1.9355 | -2.2984 | -3.2303 | 0.0329 | 6B | 0.0464 | 0.0382 | -0.0082 | 0.0344 | -0.0120 | 0.0002 |
| 07/05/2022 | 1V | 0.8314 | -0.1591 | -0.9905 | -1.1816 | -2.0130 | 0.0369 | 1B | 0.2192 | 0.1635 | -0.0557 | 0.1579 | -0.0613 | -0.0002 |
| 07/05/2022 | 2V | 0.6590 | -0.3605 | -1.0195 | -0.9598 | -1.6188 | 0.0291 | 2B | 0.1247 | 0.0731 | -0.0517 | 0.0599 | -0.0648 | 0.0000 |
| 07/05/2022 | 3V | 1.4132 | -2.0446 | -3.4578 | -3.2375 | -4.6507 | 0.0315 | 3B | 0.2240 | 0.1699 | -0.0541 | 0.1343 | -0.0896 | -0.0002 |
| 07/05/2022 | 4V | 1.6458 | -1.9082 | -3.5540 | -2.6370 | -4.2828 | 0.0470 | 4B | 0.0945 | 0.0818 | -0.0127 | 0.0988 | 0.0000 | 0.0000 |
| 07/05/2022 | 5V | 1.5943 | 0.2261 | -1.3682 | -0.5040 | -2.0984 | 0.0130 | 5B | 0.2235 | 0.1954 | -0.0281 | 0.2038 | -0.0197 | 0.0000 |
| 07/05/2022 | 6V | 1.5409 | -2.7134 | -4.2544 | -3.8673 | -5.4083 | 0.0362 | 6B | 0.0871 | 0.0814 | -0.0057 | 0.0803 | -0.0068 | 0.0001 |
| 28/05/2022 | 1V | 0.8389 | -1.5082 | -2.3471 | -2.0417 | -2.8806 | 0.0362 | 1B | 0.1270 | 0.1011 | -0.0260 | 0.1472 | 0.0000 | 0.0005 |
| 28/05/2022 | 2V | 0.7308 | -0.2110 | -0.9418 | -0.5829 | -1.3138 | 0.0283 | 2B | 0.0596 | 0.0664 | 0.0000 | 0.0602 | 0.0000 | 0.0001 |
| 28/05/2022 | 3V | 1.1918 | -0.1394 | -1.3312 | -1.0922 | -2.2840 | 0.0254 | 3B | 0.1181 | 0.1450 | 0.0000 | 0.1692 | 0.0000 | 0.0003 |
| 28/05/2022 | 4V | 1.6988 | 0.5086 | -1.1903 | -1.1993 | -2.8982 | 0.0586 | 4B | 0.0616 | 0.0622 | 0.0000 | 0.0768 | 0.0000 | 0.0000 |
| 28/05/2022 | 5V | 1.3935 | 1.6814 | 0.0000 | 0.9767 | -0.4168 | 0.0168 | 5B | 0.1982 | 0.1360 | -0.0622 | 0.1505 | -0.0477 | -0.0002 |
| 28/05/2022 | 6V | 2.0360 | -0.6304 | -2.6664 | -1.1234 | -3.1594 | 0.0568 | 6B | 0.0557 | 0.0581 | 0.0000 | 0.0610 | 0.0000 | 0.0000 |
| 27/06/2022 | 1V | 0.7880 | -1.1367 | -1.9247 | -1.9793 | -2.7673 | 0.0242 | 1B | 0.1664 | 0.1525 | -0.0138 | 0.2619 | 0.0000 | 0.0001 |
| 27/06/2022 | 2V | 0.6889 | -0.4982 | -1.1871 | -0.4102 | -1.0991 | 0.0217 | 2B | 0.2346 | 0.2063 | -0.0283 | 0.1986 | -0.0360 | 0.0001 |
| 27/06/2022 | 3V | 1.1597 | -0.7625 | -1.9222 | -0.7167 | -1.8764 | 0.0155 | 3B | 0.1615 | 0.1524 | -0.0090 | 0.1869 | 0.0000 | -0.0001 |
| 27/06/2022 | 4V | 1.9751 | 0.8852 | -1.0899 | 0.1889 | -1.7862 | 0.0493 | 4B | 0.1505 | 0.1643 | 0.0000 | 0.1319 | -0.0185 | 0.0068 |
| 27/06/2022 | 5V | 2.5586 | 1.6173 | -0.9413 | 1.5732 | -0.9854 | 0.0172 | 5B | 0.2662 | 0.2667 | 0.0000 | 0.2910 | 0.0000 | -0.0001 |
| 27/06/2022 | 6V | 2.3698 | 0.1418 | -2.2280 | -0.0899 | -2.4597 | 0.0485 | 6B | 0.1160 | 0.1121 | -0.0039 | 0.1273 | 0.0000 | -0.0001 |
| 26/07/2022 | 1V | 1.1670 | 0.0140 | -1.1530 | -0.6477 | -1.8148 | 0.0293 | 1B | 0.1717 | 0.1745 | 0.0000 | 0.1946 | 0.0000 | 0.0001 |
| 26/07/2022 | 2V | 0.8602 | -0.1107 | -0.9710 | -0.6729 | -1.5331 | 0.0230 | 2B | 0.1120 | 0.0476 | -0.0644 | 0.0277 | -0.0842 | 0.0001 |
| 26/07/2022 | 3V | 1.5969 | 0.3251 | -1.2717 | -0.5367 | -2.1336 | 0.0143 | 3B | 0.1607 | 0.1150 | -0.0456 | 0.1320 | -0.0287 | -0.0001 |
| 26/07/2022 | 4V | 1.3182 | 0.2267 | -1.0915 | -0.0837 | -1.4019 | 0.0277 | 4B | 0.0906 | 0.0848 | -0.0058 | 0.0855 | -0.0051 | 0.0014 |
| 26/07/2022 | 5V | 4.2186 | 3.7534 | -0.4652 | 2.9980 | -1.2206 | 0.0188 | 5B | 0.2418 | 0.2028 | -0.0390 | 0.1848 | -0.0570 | 0.0001 |
| 26/07/2022 | 6V | 2.9449 | 0.6096 | -2.3353 | -0.4170 | -3.3619 | 0.0375 | 6B | 0.0955 | 0.0934 | -0.0021 | 0.0931 | -0.0024 | 0.0000 |
| 28/08/2022 | 1V | 1.0791 | 0.1905 | -0.8885 | -0.2257 | -1.3048 | 0.0151 | 1B | 0.1143 | 0.1488 | 0.0000 | 0.1954 | 0.0000 | 0.0000 |
| 28/08/2022 | 2V | 0.8583 | -0.1175 | -0.9758 | -0.2751 | -1.1334 | 0.0105 | 2B | 0.0912 | 0.0925 | 0.0000 | 0.0959 | 0.0000 | 0.0001 |
| 28/08/2022 | 3V | 1.6182 | -0.5012 | -2.1193 | -0.6960 | -2.3141 | 0.0118 | 3B | 0.2688 | 0.2764 | 0.0000 | 0.2743 | 0.0000 | 0.0000 |
| 28/08/2022 | 4V | 1.4111 | 0.3942 | -1.0170 | -0.1811 | -1.5923 | 0.0215 | 4B | 0.0936 | 0.1141 | 0.0000 | 0.1042 | 0.0000 | 0.0001 |
| 28/08/2022 | 5V | 2.0227 | 1.0164 | -1.0064 | 0.8604 | -1.1623 | 0.0092 | 5B | 0.2381 | 0.2217 | -0.0163 | 0.2085 | -0.0296 | 0.0000 |
| 28/08/2022 | 6V | 2.0053 | -0.6902 | -2.6955 | -0.8128 | -2.8181 | 0.0193 | 6B | 0.0986 | 0.0923 | -0.0063 | 0.0980 | -0.0006 | -0.0001 |
| 24/09/2022 | 1V | 0.6714 | -0.7107 | -1.3820 | -0.8836 | -1.5550 | 0.0176 | 1B | 0.1966 | 0.1717 | -0.0249 | 0.1303 | -0.0663 | -0.0001 |
| 24/09/2022 | 2V | 0.5570 | -0.0443 | -0.6012 | -1.0376 | -1.5946 | 0.0203 | 2B | 0.0835 | 0.0080 | -0.0755 | -0.0227 | -0.1063 | 0.0001 |
| 24/09/2022 | 3V | 0.8472 | -0.1876 | -1.0348 | -1.1314 | -1.9787 | 0.0193 | 3B | 0.2954 | 0.2744 | -0.0210 | 0.2570 | -0.0384 | -0.0003 |
| 24/09/2022 | 4V | 0.7240 | -0.3635 | -1.0875 | -0.8978 | -1.6218 | 0.0206 | 4B | 0.0560 | 0.0532 | -0.0029 | 0.0521 | -0.0040 | 0.0000 |
| 24/09/2022 | 5V | 1.9923 | 0.8843 | -1.1080 | 0.2487 | -1.7436 | 0.0100 | 5B | 0.1824 | 0.1545 | -0.0279 | 0.1471 | -0.0353 | 0.0000 |
| 24/09/2022 | 6V | 1.5130 | -0.6315 | -2.1444 | -2.0673 | -3.5803 | 0.0313 | 6B | 0.0640 | 0.0400 | -0.0239 | 0.0458 | -0.0181 | 0.0000 |
| 18/10/2022 | 1V | 0.5573 | -1.2408 | -1.7982 | -1.8193 | -2.3766 | 0.0179 | 1B | 0.1006 | 0.0163 | -0.0843 | 0.0055 | -0.0951 | -0.0001 |
| 18/10/2022 | 2V | 0.3996 | -0.5618 | -0.9614 | -0.6904 | -1.0900 | 0.0100 | 2B | 0.0603 | -0.0204 | -0.0807 | -0.0204 | -0.0807 | 0.0012 |
| 18/10/2022 | 3V | 0.6589 | -1.1965 | -1.8555 | -1.4658 | -2.1247 | 0.0148 | 3B | 0.1108 | -0.0145 | -0.1253 | -0.0048 | -0.1156 | 0.0003 |
| 18/10/2022 | 4V | 0.3333 | -0.8870 | -1.2203 | -1.1831 | -1.5164 | 0.0123 | 4B | 0.0451 | 0.0308 | -0.0143 | 0.0263 | -0.0188 | 0.0000 |
| 18/10/2022 | 5V | 0.3399 | -0.3418 | -0.6817 | -0.4833 | -0.8231 | 0.0016 | 5B | 0.1013 | 0.0576 | -0.0437 | 0.0564 | -0.0449 | 0.0000 |
| 18/10/2022 | 6V | 0.9275 | -0.4892 | -1.4167 | -1.6595 | -2.5870 | 0.0210 | 6B | 0.0524 | 0.0389 | -0.0135 | 0.0371 | -0.0153 | 0.0001 |
| 22/11/2022 | 1V | 0.2296 | -0.2354 | -0.4650 | -0.4864 | -0.7159 | 0.0161 | 1B | 0.0483 | 0.0116 | -0.0367 | -0.0062 | -0.0545 | 0.0001 |
| 22/11/2022 | 2V | 0.2143 | 0.1186 | -0.0956 | -0.0007 | -0.2150 | 0.0105 | 2B | 0.0262 | -0.0153 | -0.0416 | -0.0315 | -0.0578 | 0.0002 |
| 22/11/2022 | 3V | 0.1977 | -0.0410 | -0.2387 | -0.2064 | -0.4040 | 0.0116 | 3B | 0.0300 |  | -0.0300 | 0.0300 | 0.0000 | 0.0003 |
| 22/11/2022 | 4V | 0.1380 | -0.0618 | -0.1998 | -0.2430 | -0.3810 | 0.0174 | 4B | 0.0385 | 0.0411 | 0.0000 | 0.0359 | -0.0026 | 0.0001 |
| 22/11/2022 | 5V | 0.4137 | 0.2856 | -0.1281 | 0.2132 | -0.2005 | 0.0047 | 5B | 0.0659 | 0.0402 | -0.0257 | 0.0303 | -0.0356 | 0.0000 |
| 22/11/2022 | 6V | 0.3345 | 0.0687 | -0.2657 | -0.1737 | -0.5082 | 0.0242 | 6B | 0.0013 | -0.0023 | -0.0036 | -0.0043 | -0.0056 | 0.0000 |
| 19/02/2023 | 1V | 0.2303 | 0.0313 | -0.1991 | -0.2468 | -0.4771 | 0.0091 | 1B | 0.0585 | 0.0466 | -0.0120 | 0.0334 | -0.0252 | -0.0001 |
| 19/02/2023 | 2V | 0.2078 | -0.1426 | -0.3504 | -0.2263 | -0.4341 | 0.0042 | 2B | 0.0407 | -0.0391 | -0.0798 | -0.0546 | -0.0953 | 0.0000 |
| 19/02/2023 | 3V | 0.2249 | 0.0465 | -0.1784 | -0.2704 | -0.4952 | 0.0088 | 3B | 0.0841 | -0.0332 | -0.1173 | -0.0557 | -0.1398 | 0.0001 |
| 19/02/2023 | 4V | 0.2126 | -0.1662 | -0.3788 | -0.4399 | -0.6525 | 0.0135 | 4B | 0.0242 | 0.0164 | -0.0077 | 0.0115 | -0.0127 | 0.0005 |
| 19/02/2023 | 5V | 0.4248 | 0.1672 | -0.2577 | 0.2535 | -0.1713 | 0.0029 | 5B | 0.0390 | 0.0211 | -0.0178 | 0.0185 | -0.0205 | 0.0000 |
| 19/02/2023 | 6V | 0.4299 | -0.1317 | -0.5616 | -0.3761 | -0.8060 | 0.0217 | 6B | 0.0227 | 0.0090 | -0.0137 | 0.0063 | -0.0164 | 0.0000 |
| 26/03/2023 | 1V | 0.3187 | -0.2206 | -0.5393 | -0.7697 | -1.0885 | 0.0102 | 1B | 0.0889 | 0.0507 | -0.0382 | 0.0416 | -0.0474 | 0.0000 |
| 26/03/2023 | 2V | 0.2482 | -0.3822 | -0.6304 | -0.6684 | -0.9166 | 0.0044 | 2B | 0.0520 | -0.0257 | -0.0777 | -0.0467 | -0.0987 | 0.0001 |
| 26/03/2023 | 3V | 0.3226 | -0.4900 | -0.8125 | -0.7816 | -1.1042 | 0.0080 | 3B | 0.0316 | -0.0491 | -0.0807 | -0.0866 | -0.1183 | 0.0000 |
| 26/03/2023 | 4V | 0.2672 | -0.6035 | -0.8707 | -0.9440 | -1.2111 | 0.0149 | 4B | 0.0367 | 0.0149 | -0.0217 | 0.0095 | -0.0272 | 0.0000 |
| 26/03/2023 | 5V | 0.4139 | 0.0775 | -0.3364 | -0.2641 | -0.6780 | 0.0030 | 5B | 0.0450 | 0.0243 | -0.0207 | 0.0160 | -0.0290 | -0.0001 |
| 26/03/2023 | 6V | 0.4869 | -0.5587 | -1.0457 | -1.1489 | -1.6358 | 0.0203 | 6B | 0.0410 | 0.0208 | -0.0201 | 0.0198 | -0.0212 | 0.0001 |
| 20/04/2023 | 1V | 0.5205 | -1.2733 | -1.7938 | -1.8403 | -2.3608 | 0.0113 | 1B | 0.0979 | 0.0609 | -0.0370 | 0.0378 | -0.0601 | -0.0001 |
| 20/04/2023 | 2V | 0.4224 | -0.9540 | -1.3764 | -1.0279 | -1.4503 | 0.0053 | 2B | 0.0599 | 0.0350 | -0.0248 | 0.0430 | -0.0169 | -0.0001 |
| 20/04/2023 | 3V | 0.6248 | -1.5510 | -2.1757 | -1.7007 | -2.3255 | 0.0099 | 3B | 0.2224 | 0.1219 | -0.1005 | 0.0763 | -0.1461 | 0.0004 |
| 20/04/2023 | 4V | 0.4795 | -1.7009 | -2.1803 | -1.8982 | -2.3777 | 0.0175 | 4B | 0.0687 | 0.0356 | -0.0330 | 0.0357 | -0.0329 | 0.0000 |
| 20/04/2023 | 5V | 0.6044 | -0.3034 | -0.9078 | -0.4433 | -1.0477 | 0.0026 | 5B | 0.0741 | 0.0488 | -0.0253 | 0.0538 | -0.0203 | 0.0000 |
| 20/04/2023 | 6V | 1.3659 | -1.8218 | -3.1877 | -1.9661 | -3.3320 | 0.0236 | 6B | 0.0724 | 0.0416 | -0.0308 | 0.0392 | -0.0332 | 0.0004 |
| 21/05/2023 | 1V | 1.5451 | -0.8350 | -2.3801 | -1.0571 | -2.6022 | 0.0138 | 1B | 0.1608 | 0.1825 | 0.0000 | 0.1536 | -0.0072 | -0.0001 |
| 21/05/2023 | 2V | 1.0455 | -0.7058 | -1.7513 | -0.6799 | -1.7254 | 0.0058 | 2B | 0.0801 | 0.0901 | 0.0000 | 0.1102 | 0.0000 | 0.0001 |
| 21/05/2023 | 3V | 1.7564 | -1.1013 | -2.8578 | -1.3531 | -3.1096 | 0.0282 | 3B | 0.8370 | 0.7450 | -0.0920 | 0.6904 | -0.1466 | 0.0029 |
| 21/05/2023 | 4V | 1.3777 | -1.8799 | -3.2576 | -1.9292 | -3.3069 | 0.0277 | 4B | 0.1058 | 0.1075 | 0.0000 | 0.1109 | 0.0000 | 0.0001 |
| 21/05/2023 | 5V | 1.1125 | 0.0572 | -1.0553 | -0.0645 | -1.1770 | 0.0024 | 5B | 0.2418 | 0.2199 | -0.0219 | 0.1889 | -0.0529 | -0.0003 |
| 21/05/2023 | 6V | 2.4938 | -0.9787 | -3.4725 | -1.3248 | -3.8185 | 0.0380 | 6B | 0.0852 | 0.0922 | 0.0000 | 0.1046 | 0.0000 | 0.0001 |
| 14/06/2023 | 1V | 1.4133 | -0.8943 | -2.3077 | -0.9779 | -2.3912 | 0.0130 | 1B | 0.1376 | 0.1246 | -0.0130 | 0.1308 | -0.0067 | -0.0003 |
| 14/06/2023 | 2V | 1.0143 | -0.2526 | -1.2670 | -0.1549 | -1.1692 | 0.0057 | 2B | 0.1737 | 0.1483 | -0.0254 | 0.1664 | -0.0073 | 0.0001 |
| 14/06/2023 | 3V | 1.5344 | -1.0782 | -2.6126 | -0.8994 | -2.4338 | 0.0336 | 3B | 0.4214 | 0.3549 | -0.0665 | 0.3639 | -0.0575 | 0.0019 |
| 14/06/2023 | 4V | 0.9317 | -0.0945 | -1.0262 | -0.1050 | -1.0367 | 0.0176 | 4B | 0.1653 | 0.1711 | 0.0000 | 0.1993 | 0.0000 | -0.0002 |
| 14/06/2023 | 5V | 0.7539 | -0.0640 | -0.8179 | -0.0730 | -0.8269 | 0.0006 | 5B | 0.2895 | 0.2872 | -0.0023 | 0.2968 | 0.0000 | -0.0008 |
| 14/06/2023 | 6V | 2.1388 | 0.1682 | -1.9707 | 0.3095 | -1.8293 | 0.0228 | 6B | 0.1745 | 0.1770 | 0.0000 | 0.1890 | 0.0000 | 0.0018 |
| 20/07/2023 | 1V | 0.9164 | 0.1220 | -0.7944 | -0.9788 | -1.8952 | 0.0111 | 1B | 0.1070 | 0.0665 | -0.0405 | 0.0808 | -0.0262 | 0.0001 |
| 20/07/2023 | 2V | 0.5732 | -0.6773 | -1.2505 | -1.4182 | -1.9914 | 0.0046 | 2B | 0.0869 | 0.0123 | -0.0746 | -0.0246 | -0.1114 | -0.0001 |
| 20/07/2023 | 3V | 0.9903 | -1.5888 | -2.5792 | -1.9851 | -2.9754 | 0.0285 | 3B | 0.1051 | 0.1190 | 0.0000 | 0.1149 | 0.0000 | 0.0003 |
| 20/07/2023 | 4V | 0.8101 | -0.9917 | -1.8018 | -1.3014 | -2.1115 | 0.0214 | 4B | 0.0808 | 0.0702 | -0.0107 | 0.0785 | -0.0023 | 0.0002 |
| 20/07/2023 | 5V | 1.4281 | 0.9427 | -0.4854 | 0.2240 | -1.2041 | 0.0043 | 5B | 0.3509 | 0.2449 | -0.1060 | 0.2302 | -0.1207 | 0.0001 |
| 20/07/2023 | 6V | 1.0718 | -1.6331 | -2.7049 | -1.6930 | -2.7648 | 0.0203 | 6B | 0.0961 | 0.1142 | 0.0000 | 0.1158 | 0.0000 | 0.0000 |
| 16/08/2023 | 1V | 1.0416 | -1.9906 | -3.0322 | -2.5940 | -3.6356 | 0.0162 | 1B | 0.1489 | 0.0598 | -0.0891 | 0.0524 | -0.0965 | -0.0001 |
| 16/08/2023 | 2V | 0.8673 | -0.9855 | -1.8528 | -1.1758 | -2.0431 | 0.0078 | 2B | 0.1816 | -0.0673 | -0.2489 | -0.0603 | -0.2419 | 0.0000 |
| 16/08/2023 | 3V | 1.1902 | -0.3247 | -1.5149 | -1.3133 | -2.5034 | 0.0377 | 3B | 0.0071 | 0.0056 | -0.0015 | 0.0122 | 0.0000 | 0.0002 |
| 16/08/2023 | 4V | 1.1109 | -0.3500 | -1.4610 | -0.5551 | -1.6660 | 0.0249 | 4B | 0.0873 | 0.0505 | -0.0368 | 0.0330 | -0.0543 | 0.0001 |
| 16/08/2023 | 5V | 0.7674 | -0.8028 | -1.5703 | -1.1526 | -1.9200 | 0.0050 | 5B | 0.2152 | 0.1395 | -0.0757 | 0.1063 | -0.1089 | 0.0000 |
| 16/08/2023 | 6V | 1.9536 | 0.8748 | -1.0788 | 0.5702 | -1.3834 | 0.0203 | 6B | 0.1729 | 0.1185 | -0.0544 | 0.1079 | -0.0650 | 0.0001 |
| 25/09/2023 | 1V | 0.7557 | -0.8648 | -1.6204 | -1.4863 | -2.2420 | 0.0093 | 1B | 0.1057 | -0.0691 | -0.1748 | -0.0042 | -0.1099 | 0.0000 |
| 25/09/2023 | 2V | 0.4980 | -0.7222 | -1.2201 | -1.3842 | -1.8822 | 0.0108 | 2B | 0.1303 | -0.0685 | -0.1988 | -0.0700 | -0.2003 | -0.0001 |
| 25/09/2023 | 3V | 0.8129 | -0.1331 | -0.9460 | -0.2828 | -1.0957 | 0.0411 | 3B | 0.0261 | -0.0075 | -0.0336 | -0.0124 | -0.0384 | 0.0071 |
| 25/09/2023 | 4V | 0.4809 | -1.3992 | -1.8800 | -1.7781 | -2.2589 | 0.0150 | 4B | 0.0997 | 0.0766 | -0.0230 | 0.0227 | -0.0770 | 0.0085 |
| 25/09/2023 | 5V | 0.6577 | -1.4166 | -2.0743 | -1.7402 | -2.3979 | 0.0058 | 5B | 0.3450 | 0.3618 | 0.0000 | 0.2278 | -0.1172 | 0.0000 |
| 25/09/2023 | 6V | 0.6658 | 0.5981 | -0.0677 | 0.5443 | -0.1215 | 0.0130 | 6B | 0.1631 | 0.0771 | -0.0860 | 0.0750 | -0.0880 | 0.0001 |
| 15/10/2023 | 1V | 0.3570 | -0.1271 | -0.4841 | -0.4335 | -0.7905 | 0.0170 | 1B | 0.0536 | -0.0279 | -0.0814 | -0.0344 | -0.0880 | 0.0002 |
| 15/10/2023 | 2V | 0.2421 | -0.6113 | -0.8534 | -0.7818 | -1.0239 | 0.0094 | 2B | 0.0610 | -0.1250 | -0.1860 | -0.1222 | -0.1832 | 0.0001 |
| 15/10/2023 | 3V | 0.3531 | -0.3843 | -0.7375 | -0.4929 | -0.8460 | 0.0244 | 3B | 0.1084 | 0.1355 | 0.0000 | 0.1091 | 0.0000 | 0.0012 |
| 15/10/2023 | 4V | 0.3685 | -1.3823 | -1.7509 | -1.5812 | -1.9497 | 0.0211 | 4B | 0.0538 | 0.0233 | -0.0305 | 0.0255 | -0.0283 | -0.0001 |
| 15/10/2023 | 5V | 0.3350 | -0.9879 | -1.3229 | -1.3547 | -1.6897 | 0.0064 | 5B | 0.1574 | 0.0705 | -0.0869 | 0.0250 | -0.1323 | 0.0001 |
| 15/10/2023 | 6V | 0.1874 | 0.1957 | 0.0000 | 0.2422 | 0.0000 | 0.0047 | 6B | 0.0609 | 0.0211 | -0.0398 | 0.0223 | -0.0385 | 0.0001 |
| 11/11/2023 | 1V | 0.2934 | 0.0732 | -0.2202 | 0.0426 | -0.2507 | 0.0138 | 1B | 0.0272 | -0.0749 | -0.1021 | -0.0752 | -0.1024 | 0.0000 |
| 11/11/2023 | 2V | 0.2713 | -0.2976 | -0.5689 | -0.4861 | -0.7574 | 0.0110 | 2B | 0.0464 | -0.1588 | -0.2051 | -0.1657 | -0.2121 | 0.0000 |
| 11/11/2023 | 3V | 0.2166 | -0.1253 | -0.3419 | -0.1955 | -0.4121 | 0.0125 | 3B | 0.0850 | 0.0859 | 0.0000 | 0.0940 | 0.0000 | 0.0004 |
| 11/11/2023 | 4V | 0.2493 | -0.8713 | -1.1206 | -1.0311 | -1.2804 | 0.0183 | 4B | 0.0284 | 0.0059 | -0.0225 | 0.0031 | -0.0253 | -0.0004 |
| 11/11/2023 | 5V | 0.1789 | -0.2587 | -0.4376 | -0.4759 | -0.6548 | 0.0053 | 5B | 0.0999 | 0.0648 | -0.0351 | 0.0499 | -0.0500 | -0.0003 |
| 11/11/2023 | 6V | 0.1794 | 0.1809 | 0.0000 | 0.1620 | -0.0174 | 0.0035 | 6B | 0.0177 | -0.0090 | -0.0267 | -0.0115 | -0.0293 | 0.0002 |
| 15/12/2023 | 1V | 0.1849 | 0.1813 | -0.0036 | 0.1728 | -0.0121 | 0.0115 | 1B | 0.0283 | -0.0138 | -0.0421 | -0.0374 | -0.0657 | 0.0000 |
| 15/12/2023 | 2V | 0.1426 | 0.1202 | -0.0224 | 0.0534 | -0.0892 | 0.0095 | 2B | 0.0304 | -0.0515 | -0.0819 | -0.1068 | -0.1372 | 0.0003 |
| 15/12/2023 | 3V | 0.1431 | 0.1283 | -0.0148 | 0.1014 | -0.0418 | 0.0091 | 3B | 0.1146 | 0.1015 | -0.0131 | 0.1175 | 0.0000 | 0.0041 |
| 15/12/2023 | 4V | 0.1178 | 0.0175 | -0.1003 | -0.0945 | -0.2122 | 0.0116 | 4B | 0.0264 | 0.0116 | -0.0148 | 0.0041 | -0.0223 | 0.0000 |
| 15/12/2023 | 5V | 0.1915 | 0.0553 | -0.1362 | -0.0711 | -0.2626 | 0.0071 | 5B | 0.0342 | 0.0118 | -0.0225 | -0.0025 | -0.0368 | 0.0000 |
| 15/12/2023 | 6V | 0.1986 | 0.1966 | -0.0021 | 0.1861 | -0.0125 | 0.0019 | 6B | 0.0341 | -0.0087 | -0.0427 | -0.0180 | -0.0520 | 0.0000 |
| 04/01/2024 | 1V | 0.1958 | 0.1661 | -0.0297 | 0.1399 | -0.0559 | 0.0128 | 1B | 0.0094 | -0.0618 | -0.0712 | -0.0725 | -0.0819 | 0.0000 |
| 04/01/2024 | 2V | 0.1356 | 0.0344 | -0.1012 | 0.0083 | -0.1273 | 0.0103 | 2B | 0.0223 | -0.1644 | -0.1867 | -0.1577 | -0.1800 | 0.0000 |
| 04/01/2024 | 3V | 0.1260 | 0.0801 | -0.0459 | 0.0691 | -0.0569 | 0.0072 | 3B | 0.0505 | 0.0605 | 0.0000 | 0.0607 | 0.0000 | -0.0003 |
| 04/01/2024 | 4V | 0.0917 | 0.0105 | -0.0812 | -0.0319 | -0.1236 | 0.0109 | 4B | 0.0238 | 0.0126 | -0.0113 | 0.0009 | -0.0230 | 0.0002 |
| 04/01/2024 | 5V | 0.1809 | 0.0995 | -0.0813 | 0.0859 | -0.0950 | 0.0079 | 5B | 0.0470 | -0.0091 | -0.0561 | -0.0273 | -0.0743 | 0.0022 |
| 04/01/2024 | 6V | 0.1562 | 0.1849 | 0.0000 | 0.1490 | -0.0073 | 0.0051 | 6B | 0.0231 | -0.0031 | -0.0261 | -0.0112 | -0.0343 | 0.0000 |
| 17/12/2020 | 1S | 0.0057 | -0.0277 | -0.0334 | -0.0287 | -0.0344 | 0.0000 | 1G | 0.1718 | 0.0687 | -0.1031 | -0.0162 | -0.1881 | 0.0012 |
| 17/12/2020 | 2S | 0.0120 | -0.0160 | -0.0280 | -0.0227 | -0.0347 | 0.0000 | 2G | 0.1284 | -0.0510 | -0.1794 | -0.1312 | -0.2596 | 0.0013 |
| 17/12/2020 | 3S | 0.0150 | -0.0188 | -0.0338 | -0.0403 | -0.0552 | 0.0000 | 3G | 0.1502 | -0.0490 | -0.1992 | -0.1268 | -0.2771 | 0.0019 |
| 17/12/2020 | 4S | 0.0295 | 0.0058 | -0.0237 | -0.0033 | -0.0328 | 0.0000 | 4G | 0.1497 | 0.0381 | -0.1116 | -0.0657 | -0.2155 | 0.0003 |
| 17/12/2020 | 5S | 0.0244 | 0.0048 | -0.0196 | -0.0091 | -0.0335 | 0.0000 | 5G | 0.2197 | 0.0947 | -0.1250 | 0.0280 | -0.1917 | 0.0006 |
| 17/12/2020 | 6S | 0.0107 | -0.0207 | -0.0314 | -0.0324 | -0.0431 | 0.0000 | 6G | 0.2074 | 0.0566 | -0.1509 | -0.2672 | -0.4746 | 0.0002 |
| 01/02/2021 | 1S | 0.0106 | -0.0131 | -0.0238 | -0.0201 | -0.0307 | 0.0000 | 1G | 0.2710 | -0.0738 | -0.3448 | -0.1641 | -0.4350 | 0.0002 |
| 01/02/2021 | 2S | 0.0103 | -0.0044 | -0.0146 | -0.0091 | -0.0193 | 0.0000 | 2G | 0.1516 | -0.0704 | -0.2220 | -0.1223 | -0.2739 | 0.0008 |
| 01/02/2021 | 3S | 0.0178 | -0.0011 | -0.0189 | -0.0113 | -0.0291 | 0.0010 | 3G | 0.1668 | -0.1098 | -0.2766 | -0.2631 | -0.4298 | 0.0004 |
| 01/02/2021 | 4S | 0.0164 | 0.0057 | -0.0107 | -0.0064 | -0.0228 | 0.0000 | 4G | 0.1477 | -0.1234 | -0.2711 | -0.1543 | -0.3020 | 0.0002 |
| 01/02/2021 | 5S | 0.0090 | -0.0001 | -0.0091 | -0.0031 | -0.0121 | -0.0015 | 5G | 0.1464 | -0.2776 | -0.4241 | -0.3162 | -0.4627 | 0.0001 |
| 01/02/2021 | 6S | 0.0121 | 0.0062 | -0.0060 | -0.0014 | -0.0135 | -0.0006 | 6G | 0.1167 | -0.1637 | -0.2803 | -0.2460 | -0.3627 | 0.0000 |
| 22/02/2021 | 1S | 0.0411 | 0.0061 | -0.0350 | -0.0042 | -0.0453 | 0.0000 | 1G | 0.0000 | -0.0197 | -0.0197 | -0.1374 | -0.1375 | 0.0004 |
| 22/02/2021 | 2S | 0.0528 | 0.0098 | -0.0430 | 0.0064 | -0.0464 | 0.0000 | 2G | 0.1409 | -0.1965 | -0.3374 | -0.2656 | -0.4065 | 0.0005 |
| 22/02/2021 | 3S | 0.0390 | 0.0096 | -0.0294 | -0.0035 | -0.0425 | 0.0000 | 3G | 0.2479 | -0.2221 | -0.4700 | -0.4131 | -0.6610 | 0.0010 |
| 22/02/2021 | 4S | 0.0657 | -0.0241 | -0.0898 | -0.0259 | -0.0916 | 0.0000 | 4G | 0.2379 | 0.0244 | -0.2135 | -0.1602 | -0.3981 | 0.0002 |
| 22/02/2021 | 5S | 0.0474 | -0.0156 | -0.0630 | -0.0178 | -0.0651 | 0.0000 | 5G | 0.3206 | -0.1886 | -0.5092 | -0.6926 | -1.0132 | 0.0000 |
| 22/02/2021 | 6S | 0.0621 | 0.0204 | -0.0417 | 0.0155 | -0.0466 | 0.0001 | 6G | 0.3066 | -0.5560 | -0.8625 | -0.4820 | -0.7886 | 0.0000 |
| 30/03/2021 | 1S | 0.0465 | -0.0301 | -0.0766 | -0.0347 | -0.0812 | 0.0000 | 1G | 0.8712 | -0.3323 | -1.2035 | -0.4928 | -1.3640 | 0.0012 |
| 30/03/2021 | 2S | 0.0594 | -0.0137 | -0.0731 | -0.0270 | -0.0864 | 0.0000 | 2G | 0.6443 | -0.2738 | -0.9181 | -0.3598 | -1.0041 | 0.0042 |
| 30/03/2021 | 3S | 0.0791 | -0.0383 | -0.1174 | -0.0537 | -0.1328 | 0.0000 | 3G | 0.7466 | -0.4861 | -1.2327 | -0.7128 | -1.4594 | 0.0037 |
| 30/03/2021 | 4S | 0.1194 | -0.0477 | -0.1671 | -0.0774 | -0.1968 | 0.0000 | 4G | 0.6818 | -0.3984 | -1.0802 | -0.6448 | -1.3266 | 0.0015 |
| 30/03/2021 | 5S | 0.0961 | -0.0154 | -0.1115 | -0.0311 | -0.1272 | 0.0000 | 5G | 0.8284 | -0.5515 | -1.3800 | -0.7759 | -1.6043 | 0.0001 |
| 30/03/2021 | 6S | 0.0988 | -0.0318 | -0.1306 | -0.0473 | -0.1461 | 0.0001 | 6G | 0.8628 | -0.3810 | -1.2438 | -0.5259 | -1.3888 | 0.0000 |
| 24/04/2021 | 1S | 0.1058 | 0.0087 | -0.0971 | 0.0076 | -0.0982 | -0.0002 | 1G | 1.4495 | -0.1328 | -1.5822 | -0.4171 | -1.8665 | -0.0009 |
| 24/04/2021 | 2S | 0.0987 | 0.0154 | -0.0832 | 0.0269 | -0.0718 | 0.0000 | 2G | 1.3100 | 0.0862 | -1.2238 | -0.2227 | -1.5327 | -0.0014 |
| 24/04/2021 | 3S | 0.1243 | -0.0272 | -0.1516 | -0.0461 | -0.1705 | -0.0001 | 3G | 1.3298 | -0.0987 | -1.4285 | -0.4150 | -1.7449 | -0.0011 |
| 24/04/2021 | 4S | 0.1580 | 0.0250 | -0.1330 | 0.0287 | -0.1293 | -0.0001 | 4G | 1.3095 | -0.1716 | -1.4811 | -0.3309 | -1.6403 | -0.0006 |
| 24/04/2021 | 5S | 0.1298 | -0.0313 | -0.1611 | -0.0349 | -0.1647 | 0.0000 | 5G | 1.2955 | -0.2749 | -1.5703 | -0.4007 | -1.6961 | -0.0014 |
| 24/04/2021 | 6S | 0.1118 | -0.0361 | -0.1479 | -0.0450 | -0.1568 | 0.0000 | 6G | 1.2115 | -0.1939 | -1.4054 | -0.3991 | -1.6106 | -0.0012 |
| 19/05/2021 | 1S | 0.1499 | -0.0172 | -0.1670 | -0.0256 | -0.1754 | 0.0000 | 1G | 1.4261 | -0.4597 | -1.8857 | -0.9229 | -2.3490 | -0.0005 |
| 19/05/2021 | 2S | 0.1116 | -0.0247 | -0.1363 | -0.0250 | -0.1367 | 0.0000 | 2G | 3.0128 | 1.5005 | -1.5123 | -0.8924 | -3.9052 | -0.0008 |
| 19/05/2021 | 3S | 0.1250 | -0.0856 | -0.2107 | -0.1047 | -0.2297 | 0.0001 | 3G | 1.2232 | -0.5366 | -1.7598 | -1.1207 | -2.3438 | -0.0001 |
| 19/05/2021 | 4S | 0.1878 | -0.0269 | -0.2147 | -0.0528 | -0.2406 | 0.0000 | 4G | 1.1289 | -1.1152 | -2.2440 | -1.3717 | -2.5006 | -0.0003 |
| 19/05/2021 | 5S | 0.1265 | -0.0539 | -0.1804 | -0.0677 | -0.1942 | 0.0001 | 5G | 2.9449 | -0.5035 | -3.4484 | -1.5264 | -4.4713 | -0.0014 |
| 19/05/2021 | 6S | 0.1521 | -0.0476 | -0.1997 | -0.0723 | -0.2244 | 0.0000 | 6G |  |  |  |  |  |  |
| 13/06/2021 | 1S | 0.1385 | 0.0651 | -0.0734 | 0.0242 | -0.1142 | 0.0000 | 1G | 2.0067 | -0.2262 | -2.2328 | -1.1095 | -3.1162 | -0.0025 |
| 13/06/2021 | 2S | 0.1990 | 0.0335 | -0.1655 | 0.0356 | -0.1635 | 0.0000 | 2G | 1.9689 | -0.0561 | -2.0249 | -0.1358 | -2.1046 | -0.0029 |
| 13/06/2021 | 3S | 0.1840 | -0.0697 | -0.2537 | -0.1003 | -0.2843 | 0.0000 | 3G | 2.0966 | -0.2106 | -2.3072 | -1.2285 | -3.3251 | -0.0017 |
| 13/06/2021 | 4S | 0.2777 | 0.1075 | -0.1702 | 0.0825 | -0.1952 | 0.0000 | 4G | 2.0253 | -0.3643 | -2.3896 | -1.7176 | -3.7429 | -0.0015 |
| 13/06/2021 | 5S | 0.1897 | 0.0358 | -0.1539 | -0.0427 | -0.2324 | 0.0000 | 5G | 1.6291 | -0.3696 | -1.9987 | -1.2297 | -2.8588 | -0.0014 |
| 13/06/2021 | 6S | 0.2073 | -0.0041 | -0.2114 | -0.0620 | -0.2693 | -0.0002 | 6G | 0.6097 | -0.2363 | -0.8460 | -0.6296 | -1.2393 | -0.0008 |
| 16/07/2021 | 1S | 0.2725 | 0.0018 | -0.2708 | -0.0307 | -0.3032 | -0.0001 | 1G | 2.3664 | 0.4867 | -1.8798 | -0.6624 | -3.0289 | -0.0010 |
| 16/07/2021 | 2S | 0.2288 | 0.0495 | -0.1794 | -0.0158 | -0.2447 | 0.0000 | 1G |  | -0.2324 | -2.5989 |  |  |  |
| 16/07/2021 | 2S |  | 0.0086 | -0.2202 |  |  |  | 1G |  | -0.6997 | -3.0661 |  |  |  |
| 16/07/2021 | 2S |  | -0.0220 | -0.2508 |  |  |  | 2G | 1.8479 | -0.4876 | -2.3355 | -0.6623 | -2.5102 | -0.0012 |
| 16/07/2021 | 3S | 0.2598 | -0.0304 | -0.2902 | -0.1395 | -0.3993 | 0.0000 | 3G | 1.8437 | -0.4279 | -2.2716 | -0.4547 | -2.2984 | -0.0011 |
| 16/07/2021 | 3S |  | -0.1006 | -0.3605 |  |  |  | 4G | 1.5077 | 0.0945 | -1.4132 | -0.4835 | -1.9912 | -0.0005 |
| 16/07/2021 | 3S |  | -0.1237 | -0.3835 |  |  |  | 4G |  | -0.0269 | -1.5346 |  |  |  |
| 16/07/2021 | 4S | 0.3328 | 0.1913 | -0.1415 | 0.1227 | -0.2101 | 0.0001 | 4G |  | -0.1046 | -1.6123 |  |  |  |
| 16/07/2021 | 4S |  | 0.1326 | -0.2002 |  |  |  | 5G | 1.5095 | -0.2843 | -1.7939 | -0.7652 | -2.2747 | -0.0012 |
| 16/07/2021 | 4S |  | 0.1159 | -0.2169 |  |  |  | 5G |  | -0.5983 | -2.1078 |  |  |  |
| 16/07/2021 | 5S | 0.8926 | 0.5326 | -0.3600 | 0.4973 | -0.3953 | 0.0001 | 5G |  | -0.7860 | -2.2955 |  |  |  |
| 16/07/2021 | 6S | 0.3021 | -0.0189 | -0.3210 | -0.0642 | -0.3663 | -0.0003 | 6G | 2.0239 | -0.5373 | -2.5612 | -0.8593 | -2.8833 | -0.0016 |
| 26/08/2021 | 1S | 0.2558 | -0.0885 | -0.3443 | -0.1150 | -0.3708 | 0.0000 | 1G | 2.1191 | 0.1454 | -1.9737 | -0.6689 | -2.7880 | -0.0014 |
| 26/08/2021 | 2S | 0.2333 | 0.0056 | -0.2277 | -0.0577 | -0.2909 | 0.0002 | 1G |  | -0.2962 | -2.4153 |  |  |  |
| 26/08/2021 | 2S |  | -0.0348 | -0.2681 |  |  |  | 1G |  | -0.6141 | -2.7332 |  |  |  |
| 26/08/2021 | 2S |  | -0.0541 | -0.2873 |  |  |  | 2G | 2.0831 | -0.2065 | -2.2895 | -0.5706 | -2.6537 | -0.0017 |
| 26/08/2021 | 3S | 0.2211 | -0.0652 | -0.2863 | -0.1512 | -0.3723 | 0.0001 | 3G | 1.8770 | -0.3889 | -2.2659 | -0.8738 | -2.7508 | -0.0013 |
| 26/08/2021 | 3S |  | -0.1103 | -0.3314 |  |  |  | 4G | 2.2148 | 0.6047 | -1.6101 | -0.3760 | -2.5908 | -0.0011 |
| 26/08/2021 | 3S |  | -0.1562 | -0.3773 |  |  |  | 4G |  | 0.1550 | -2.0598 |  |  |  |
| 26/08/2021 | 4S | 0.2109 | 0.0492 | -0.1617 | 0.0252 | -0.1857 | 0.0001 | 4G |  | -0.3086 | -2.5235 |  |  |  |
| 26/08/2021 | 4S |  | 0.0110 | -0.1999 |  |  |  | 5G | 1.4958 | -0.2819 | -1.7777 | -1.2148 | -2.7106 | -0.0014 |
| 26/08/2021 | 4S |  | -0.0111 | -0.2219 |  |  |  | 5G |  | -0.8672 | -2.3630 |  |  |  |
| 26/08/2021 | 5S | 0.1835 | 0.0249 | -0.1585 | 0.0251 | -0.1583 | 0.0001 | 5G |  | -1.1973 | -2.6932 |  |  |  |
| 26/08/2021 | 6S | 0.2485 | -0.0871 | -0.3355 | -0.1241 | -0.3725 | 0.0003 | 6G | 1.6628 | -0.5419 | -2.2047 | -0.9221 | -2.5849 | -0.0016 |
| 20/09/2021 | 1S | 0.1995 | -0.0105 | -0.2099 | -0.0800 | -0.2795 | -0.0001 | 1G | 1.1260 | -0.7289 | -1.8549 | -0.8040 | -1.9300 | -0.0015 |
| 20/09/2021 | 1S |  | -0.0631 | -0.2625 |  |  |  | 2G | 1.4223 | 0.1980 | -1.2243 | -0.7134 | -2.1357 | -0.0024 |
| 20/09/2021 | 1S |  | -0.1000 | -0.2994 |  |  |  | 2G |  | -0.3347 | -1.7570 |  |  |  |
| 20/09/2021 | 2S | 0.1904 | -0.0005 | -0.1909 | -0.0131 | -0.2035 | 0.0006 | 2G |  | -0.5222 | -1.9445 |  |  |  |
| 20/09/2021 | 3S | 0.1975 | -0.0536 | -0.2511 | -0.0795 | -0.2769 | 0.0001 | 3G | 1.2000 | -0.9137 | -2.1137 | -0.6921 | -1.8921 | -0.0014 |
| 20/09/2021 | 4S | 0.2355 | 0.0763 | -0.1592 | 0.0586 | -0.1769 | -0.0002 | 4G | 1.4304 | 0.9541 | -0.4762 | -0.1879 | -1.6182 | -0.0020 |
| 20/09/2021 | 5S | 0.2028 | 0.0598 | -0.1430 | 0.0223 | -0.1805 | -0.0003 | 4G |  | 0.3376 | -1.0928 |  |  |  |
| 20/09/2021 | 5S |  | 0.0094 | -0.1934 |  |  |  | 4G |  | -0.1252 | -1.5556 |  |  |  |
| 20/09/2021 | 5S |  | -0.0636 | -0.2664 |  |  |  | 5G | 0.9446 | -0.2993 | -1.2439 | -0.8421 | -1.7868 | -0.0012 |
| 20/09/2021 | 6S | 0.1784 | 0.0992 | -0.0792 | -0.0474 | -0.2258 | 0.0000 | 5G |  | -0.8274 | -1.7721 |  |  |  |
| 20/09/2021 | 6S |  | -0.0435 | -0.2219 |  |  |  | 5G |  | -0.8242 | -1.7688 |  |  |  |
| 20/09/2021 | 6S |  | -0.0523 | -0.2307 |  |  |  | 6G | 1.0641 | -0.3784 | -1.4425 | -0.4278 | -1.4919 | -0.0017 |
| 16/10/2021 | 1S | 0.0511 | -0.0890 | -0.1401 | -0.1379 | -0.1890 | -0.0001 | 1G | 0.7155 | 0.1758 | -0.5397 | -0.2530 | -0.9685 | -0.0003 |
| 16/10/2021 | 2S | 0.0561 | -0.0849 | -0.1411 | -0.1049 | -0.1610 | 0.0003 | 2G | 0.6144 | -0.1227 | -0.7372 | -0.4273 | -1.0417 | -0.0004 |
| 16/10/2021 | 3S | 0.0588 | -0.0853 | -0.1440 | -0.1248 | -0.1836 | 0.0002 | 3G | 0.5395 | -0.1238 | -0.6633 | -0.4594 | -0.9989 | -0.0004 |
| 16/10/2021 | 4S | 0.0772 | -0.1147 | -0.1919 | -0.1142 | -0.1915 | -0.0005 | 4G | 0.4369 | -0.0586 | -0.4955 | -0.3300 | -0.7669 | -0.0005 |
| 16/10/2021 | 5S | 0.0753 | -0.0808 | -0.1561 | -0.1215 | -0.1968 | -0.0002 | 5G | 0.5796 | -0.1075 | -0.6871 | -0.2126 | -0.7922 | -0.0006 |
| 16/10/2021 | 6S | 0.0730 | -0.1167 | -0.1897 | -0.1721 | -0.2451 | 0.0000 | 6G | 0.5185 | -0.2101 | -0.7286 | -0.6549 | -1.1733 | -0.0004 |
| 14/11/2021 | 1S | 0.0360 | -0.1359 | -0.1719 | -0.1459 | -0.1818 | 0.0001 | 1G | 0.6246 | -0.4326 | -1.0572 | -0.5324 | -1.1570 | 0.0005 |
| 14/11/2021 | 2S | 0.0464 | -0.1368 | -0.1832 | -0.1424 | -0.1888 | 0.0003 | 2G | 0.7476 | -0.1701 | -0.9177 | -0.2362 | -0.9838 | -0.0004 |
| 14/11/2021 | 3S | 0.0544 | -0.1062 | -0.1606 | -0.1451 | -0.1995 | 0.0000 | 3G | 0.4090 | -0.6059 | -1.0149 | -0.7229 | -1.1319 | 0.0000 |
| 14/11/2021 | 4S | 0.0655 | -0.1036 | -0.1691 | -0.1101 | -0.1756 | 0.0000 | 4G | 0.9395 | 0.6050 | -0.3345 | 0.2418 | -0.6977 | -0.0003 |
| 14/11/2021 | 5S | 0.0489 | -0.0777 | -0.1266 | -0.0863 | -0.1352 | 0.0000 | 5G | 0.7068 | 0.1927 | -0.5141 | -0.0259 | -0.7327 | -0.0003 |
| 14/11/2021 | 6S | 0.0472 | -0.1257 | -0.1729 | -0.1412 | -0.1884 | 0.0001 | 6G | 0.5982 | 0.2803 | -0.3179 | 0.0871 | -0.5111 | -0.0004 |
| 14/12/2021 | 1S | 0.0216 | -0.0289 | -0.0505 | -0.0622 | -0.0838 | 0.0000 | 1G | 0.2576 | -0.0701 | -0.3277 | -0.2138 | -0.4714 | 0.0001 |
| 14/12/2021 | 2S | 0.0268 | -0.0490 | -0.0758 | -0.0873 | -0.1140 | 0.0000 | 2G | 0.2892 | -0.1254 | -0.4146 | -0.1761 | -0.4654 | 0.0002 |
| 14/12/2021 | 3S | 0.0280 | -0.0396 | -0.0676 | -0.0590 | -0.0870 | 0.0000 | 3G | 0.1974 | 0.0033 | -0.1941 | -0.1105 | -0.3079 | 0.0001 |
| 14/12/2021 | 4S | 0.0416 | -0.0137 | -0.0553 | -0.0504 | -0.0920 | 0.0000 | 4G | 0.3151 | -0.0837 | -0.3988 | -0.1433 | -0.4584 | -0.0001 |
| 14/12/2021 | 5S | 0.0370 | 0.0103 | -0.0267 | -0.0403 | -0.0773 | 0.0000 | 5G | 0.4089 | -0.0405 | -0.4493 | -0.1782 | -0.5871 | -0.0001 |
| 14/12/2021 | 6S | 0.0282 | -0.0470 | -0.0752 | -0.0599 | -0.0881 | 0.0000 | 6G | 0.2796 | -0.1036 | -0.3832 | -0.2281 | -0.5077 | -0.0002 |
| 05/01/2022 | 1S | 0.0161 | -0.0702 | -0.0863 | -0.0857 | -0.1019 | 0.0000 | 1G | 0.2017 | -0.3016 | -0.5033 | -0.4036 | -0.6053 | 0.0010 |
| 05/01/2022 | 2S | 0.0129 | -0.0668 | -0.0797 | -0.0793 | -0.0923 | 0.0000 | 2G | 0.1496 | -0.1557 | -0.3053 | -0.2370 | -0.3866 | -0.0001 |
| 05/01/2022 | 3S | 0.0190 | -0.0684 | -0.0875 | -0.0847 | -0.1038 | 0.0001 | 3G | 0.1895 | -0.1900 | -0.3795 | -0.2958 | -0.4853 | 0.0017 |
| 05/01/2022 | 4S | 0.0278 | -0.0664 | -0.0942 | -0.0734 | -0.1012 | 0.0000 | 4G | 0.1556 | -0.1306 | -0.2862 | -0.2177 | -0.3733 | 0.0000 |
| 05/01/2022 | 5S | 0.0159 | -0.0543 | -0.0702 | -0.0497 | -0.0656 | 0.0000 | 5G | 0.1799 | -0.3526 | -0.5325 | -0.3885 | -0.5684 | -0.0001 |
| 05/01/2022 | 6S | 0.0208 | -0.0683 | -0.0890 | -0.0844 | -0.1051 | 0.0000 | 6G | 0.1888 | -0.2961 | -0.4849 | -0.3667 | -0.5555 | -0.0001 |
| 25/02/2022 | 1S | 0.0267 | -0.0959 | -0.1226 | -0.1162 | -0.1428 | 0.0001 | 1G | 0.2758 | -0.6484 | -0.9241 | -0.7148 | -0.9906 | 0.0003 |
| 25/02/2022 | 2S | 0.0429 | -0.0771 | -0.1200 | -0.1000 | -0.1429 | 0.0000 | 2G | 0.2070 | -0.5262 | -0.7332 | -0.5721 | -0.7791 | -0.0002 |
| 25/02/2022 | 3S | 0.0365 | -0.0957 | -0.1322 | -0.1173 | -0.1538 | -0.0001 | 3G | 0.2172 | -0.5127 | -0.7298 | -0.6548 | -0.8720 | 0.0009 |
| 25/02/2022 | 4S | 0.0572 | -0.0823 | -0.1395 | -0.0833 | -0.1404 | 0.0000 | 4G | 0.2548 | -0.7190 | -0.9737 | -0.8161 | -1.0708 | 0.0000 |
| 25/02/2022 | 5S | 0.0528 | -0.0438 | -0.0966 | -0.0714 | -0.1242 | 0.0001 | 5G | 0.2790 | -0.3093 | -0.5883 | -0.7632 | -1.0422 | -0.0001 |
| 25/02/2022 | 6S | 0.0405 | -0.0314 | -0.0719 | -0.0742 | -0.1147 | 0.0000 | 6G | 0.3404 | -0.5499 | -0.8903 | -1.0964 | -1.4368 | -0.0001 |
| 20/04/2022 | 1S | 0.1489 | -0.1731 | -0.3219 | -0.1926 | -0.3415 | -0.0001 | 1G | 1.6120 | -0.2914 | -1.9034 | -0.3355 | -1.9475 | -0.0005 |
| 20/04/2022 | 2S | 0.1535 | -0.1177 | -0.2712 | -0.1372 | -0.2907 | -0.0001 | 2G | 1.5861 | -0.4739 | -2.0600 | -0.7628 | -2.3489 | -0.0006 |
| 20/04/2022 | 3S | 0.1534 | -0.1541 | -0.3075 | -0.1967 | -0.3501 | -0.0001 | 3G | 1.4770 | -0.2430 | -1.7200 | -0.6164 | -2.0935 | -0.0002 |
| 20/04/2022 | 4S | 0.1820 | -0.0695 | -0.2515 | -0.0928 | -0.2748 | 0.0002 | 4G | 1.6379 | -0.8146 | -2.4525 | -1.2413 | -2.8792 | -0.0002 |
| 20/04/2022 | 5S | 0.1603 | -0.0892 | -0.2495 | -0.1266 | -0.2870 | 0.0000 | 5G | 1.4924 | -0.4991 | -1.9915 | -0.9828 | -2.4752 | -0.0005 |
| 20/04/2022 | 6S | 0.1804 | -0.1843 | -0.3647 | -0.2424 | -0.4228 | 0.0001 | 6G | 1.5703 | -0.6998 | -2.2702 | -1.4811 | -3.0514 | -0.0005 |
| 08/05/2022 | 1S | 0.1686 | -0.1233 | -0.2919 | -0.1729 | -0.3415 | 0.0000 | 1G | 1.0190 | -0.7542 | -1.7733 | -1.6433 | -2.6624 | -0.0004 |
| 08/05/2022 | 2S | 0.1687 | -0.0900 | -0.2587 | -0.1183 | -0.2870 | 0.0000 | 2G | 1.5324 | -1.1495 | -2.6819 | -1.0067 | -2.5391 | -0.0005 |
| 08/05/2022 | 3S | 0.1545 | -0.1671 | -0.3216 | -0.2143 | -0.3688 | -0.0001 | 3G | 1.0123 | -0.6995 | -1.7118 | -1.3662 | -2.3786 | -0.0002 |
| 08/05/2022 | 4S | 0.1926 | -0.0952 | -0.2879 | -0.1217 | -0.3144 | 0.0001 | 4G | 1.4649 | -0.5083 | -1.9732 | -1.8495 | -3.3143 | -0.0005 |
| 08/05/2022 | 5S | 0.1823 | -0.1014 | -0.2837 | -0.1427 | -0.3250 | 0.0000 | 5G | 1.3662 | -1.0853 | -2.4515 | -1.7076 | -3.0738 | -0.0006 |
| 08/05/2022 | 6S | 0.1941 | -0.1562 | -0.3502 | -0.2122 | -0.4063 | 0.0000 | 6G | 1.5749 | -1.1581 | -2.7330 | -1.7157 | -3.2906 | -0.0006 |
| 27/05/2022 | 1S | 0.1348 | -0.0123 | -0.1471 | -0.0989 | -0.2337 | 0.0000 | 1G | 1.6014 | -0.7658 | -2.3673 | -1.1685 | -2.7699 | -0.0006 |
| 27/05/2022 | 2S | 0.1126 | -0.0969 | -0.2095 | -0.1208 | -0.2334 | 0.0001 | 2G | 2.7508 | 0.2035 | -2.5473 | -1.1144 | -3.8651 | -0.0012 |
| 27/05/2022 | 3S | 0.1050 | -0.1909 | -0.2959 | -0.2218 | -0.3268 | -0.0001 | 3G | 1.7007 | 0.1122 | -1.5885 | -0.1002 | -1.8009 | -0.0009 |
| 27/05/2022 | 4S | 0.2105 | -0.0642 | -0.2748 | -0.1052 | -0.3157 | -0.0001 | 4G | 1.5841 | -0.9053 | -2.4894 | -1.7774 | -3.3615 | -0.0007 |
| 27/05/2022 | 5S | 0.1650 | -0.0797 | -0.2448 | -0.1847 | -0.3497 | 0.0001 | 5G | 1.3313 | -1.3234 | -2.6547 | -0.7378 | -2.0691 | -0.0010 |
| 27/05/2022 | 6S | 0.1834 | -0.2313 | -0.4147 | -0.3233 | -0.5068 | -0.0001 | 6G | 2.0326 | -1.3917 | -3.4244 | -1.9640 | -3.9966 | -0.0012 |
| 02/07/2022 | 1S | 0.1036 | 0.0016 | -0.1020 | -0.0149 | -0.1186 | 0.0000 | 1G | 2.5091 | -0.5556 | -3.0647 | -0.3002 | -2.8092 | -0.0007 |
| 02/07/2022 | 2S | 0.1075 | -0.0027 | -0.1102 | -0.0271 | -0.1346 | 0.0001 | 2G | 2.5505 | 0.8520 | -1.6984 | -1.7613 | -4.3118 | -0.0008 |
| 02/07/2022 | 3S | 0.0531 | -0.0149 | -0.0680 | -0.0198 | -0.0729 | 0.0000 | 3G | 2.5362 | 0.8098 | -1.7264 | -0.2675 | -2.8037 | -0.0007 |
| 02/07/2022 | 4S | 0.1725 | 0.0409 | -0.1316 | -0.0305 | -0.2030 | 0.0000 | 4G | 1.8511 | -0.6010 | -2.4521 | -1.7271 | -3.5782 | -0.0004 |
| 02/07/2022 | 5S | 0.1455 | -0.0345 | -0.1800 | -0.0564 | -0.2020 | 0.0000 | 5G | 2.0564 | -0.9310 | -2.9874 | -1.9781 | -4.0345 | -0.0011 |
| 02/07/2022 | 6S | 0.1009 | -0.0607 | -0.1616 | -0.0784 | -0.1792 | 0.0000 | 6G | 2.1678 | -0.0800 | -2.2478 | -2.1537 | -4.3215 | -0.0007 |
| 27/07/2022 | 1S | 0.2650 | -0.0588 | -0.3238 | -0.1113 | -0.3763 | -0.0001 | 1G | 2.3174 | 0.8326 | -1.4848 | 0.1832 | -2.1341 | -0.0015 |
| 27/07/2022 | 2S | 0.2488 | -0.0165 | -0.2653 | -0.0944 | -0.3432 | 0.0006 | 2G | 2.1790 | 1.1267 | -1.0523 | 0.3974 | -1.7816 | -0.0019 |
| 27/07/2022 | 3S | 0.2616 | -0.0146 | -0.2761 | -0.1142 | -0.3758 | -0.0001 | 3G | 2.0063 | 0.6181 | -1.3882 | -0.5156 | -2.5220 | -0.0017 |
| 27/07/2022 | 4S | 0.3266 | 0.0643 | -0.2624 | 0.0225 | -0.3041 | 0.0000 | 4G | 2.2262 | -1.0571 | -3.2832 | -1.9644 | -4.1906 | -0.0013 |
| 27/07/2022 | 5S | 0.2865 | 0.0300 | -0.2565 | -0.0360 | -0.3225 | 0.0000 | 5G | 1.9333 | -1.0599 | -2.9931 | -1.5369 | -3.4702 | -0.0014 |
| 27/07/2022 | 6S | 0.2126 | -0.0430 | -0.2556 | -0.1401 | -0.3527 | 0.0001 | 6G | 1.8777 | -0.1612 | -2.0390 | -1.3561 | -3.2338 | -0.0020 |
| 26/08/2022 | 1S | 0.2742 | -0.0889 | -0.3631 | -0.1600 | -0.4342 | 0.0000 | 1G | 2.5014 | 0.7290 | -1.7724 | -0.2899 | -2.7913 | -0.0008 |
| 26/08/2022 | 2S | 0.2430 | -0.0630 | -0.3060 | -0.1303 | -0.3733 | 0.0002 | 2G | 2.4607 | 0.9732 | -1.4875 | 0.4319 | -2.0288 | -0.0020 |
| 26/08/2022 | 3S | 0.2231 | 0.0017 | -0.2214 | -0.0674 | -0.2905 | 0.0000 | 3G | 1.9418 | 0.1918 | -1.7500 | -0.8730 | -2.8148 | -0.0020 |
| 26/08/2022 | 4S | 0.3055 | 0.0548 | -0.2508 | -0.0194 | -0.3249 | 0.0001 | 4G | 2.0120 | 0.3916 | -1.6204 | -1.0990 | -3.1109 | -0.0016 |
| 26/08/2022 | 5S | 0.2787 | -0.0003 | -0.2790 | -0.0645 | -0.3432 | 0.0000 | 5G | 1.6001 | -0.2726 | -1.8726 | -0.5035 | -2.1035 | -0.0016 |
| 26/08/2022 | 6S | 0.2485 | -0.1085 | -0.3570 | -0.2159 | -0.4644 | 0.0000 | 6G | 2.0782 | 0.6322 | -1.4459 | -0.3621 | -2.4402 | -0.0020 |
| 23/09/2022 | 1S | 0.1884 | -0.1350 | -0.3234 | -0.1734 | -0.3618 | 0.0000 | 1G | 1.8131 | -0.8171 | -2.6302 | -1.5412 | -3.3543 | -0.0014 |
| 23/09/2022 | 2S | 0.1798 | -0.0637 | -0.2434 | -0.1912 | -0.3710 | 0.0002 | 2G | 1.5431 | -0.5114 | -2.0545 | -0.5970 | -2.1401 | -0.0008 |
| 23/09/2022 | 3S | 0.1504 | -0.0297 | -0.1801 | -0.1387 | -0.2891 | -0.0001 | 3G | 1.2982 | -1.1274 | -2.4256 | -1.6725 | -2.9707 | -0.0014 |
| 23/09/2022 | 4S | 0.1894 | -0.0226 | -0.2120 | -0.1058 | -0.2952 | 0.0000 | 4G | 1.6334 | -0.6738 | -2.3072 | -1.5096 | -3.1430 | -0.0006 |
| 23/09/2022 | 5S | 0.1672 | -0.0290 | -0.1962 | -0.1310 | -0.2983 | 0.0000 | 5G | 1.0317 | -0.4633 | -1.4951 | -0.6894 | -1.7211 | -0.0009 |
| 23/09/2022 | 6S | 0.1259 | -0.0703 | -0.1961 | -0.2003 | -0.3262 | 0.0000 | 6G | 1.5556 | -0.4436 | -1.9993 | -1.4302 | -2.9858 | -0.0011 |
| 22/10/2022 | 1S | 0.0618 | -0.1190 | -0.1808 | -0.1573 | -0.2191 | 0.0000 | 1G | 1.1761 | -1.2204 | -2.3965 | -1.5561 | -2.7323 | -0.0001 |
| 22/10/2022 | 2S | 0.0763 | -0.1308 | -0.2071 | -0.1515 | -0.2278 | 0.0001 | 2G | 1.0167 | -0.7883 | -1.8050 | -1.0305 | -2.0472 | -0.0001 |
| 22/10/2022 | 3S | 0.0514 | -0.0959 | -0.1473 | -0.1137 | -0.1652 | 0.0001 | 3G | 1.0370 | -0.9992 | -2.0362 | -1.6202 | -2.6572 | -0.0004 |
| 22/10/2022 | 4S | 0.0898 | -0.1285 | -0.2182 | -0.1363 | -0.2261 | 0.0000 | 4G | 1.2501 | -0.8524 | -2.1024 | -1.1843 | -2.4344 | -0.0005 |
| 22/10/2022 | 5S | 0.0847 | -0.0766 | -0.1613 | -0.1048 | -0.1895 | 0.0000 | 5G | 1.3179 | 0.1785 | -1.1393 | -1.1039 | -2.4218 | -0.0010 |
| 22/10/2022 | 6S | 0.0735 | -0.0956 | -0.1690 | -0.1836 | -0.2570 | 0.0000 | 6G | 1.3173 | -0.6041 | -1.9214 | -0.8597 | -2.1770 | -0.0009 |
| 19/11/2022 | 1S | 0.0127 | -0.0767 | -0.0894 | -0.0805 | -0.0931 | 0.0000 | 1G | 0.3519 | -1.1518 | -1.5037 | -1.1750 | -1.5269 | 0.0010 |
| 19/11/2022 | 2S | 0.0334 | -0.0904 | -0.1238 | -0.1120 | -0.1454 | -0.0001 | 2G | 0.3636 | -0.4306 | -0.7943 | -0.4927 | -0.8563 | 0.0001 |
| 19/11/2022 | 3S | 0.0094 | -0.0616 | -0.0710 | -0.0631 | -0.0725 | 0.0001 | 3G | 0.3641 | -0.8787 | -1.2427 | -1.0577 | -1.4218 | 0.0011 |
| 19/11/2022 | 4S | 0.0377 | -0.1034 | -0.1411 | -0.1212 | -0.1590 | 0.0000 | 4G | 0.5238 | -0.7173 | -1.2410 | -0.8706 | -1.3944 | 0.0005 |
| 19/11/2022 | 5S | 0.0361 | -0.0700 | -0.1061 | -0.0713 | -0.1074 | 0.0000 | 5G | 0.5332 | -0.8785 | -1.4117 | -0.9449 | -1.4781 | -0.0003 |
| 19/11/2022 | 6S | 0.0234 | -0.0827 | -0.1061 | -0.1017 | -0.1251 | 0.0001 | 6G | 0.7645 | -0.6153 | -1.3798 | -0.9671 | -1.7315 | -0.0001 |
| 14/02/2023 | 1S | 0.0312 | -0.1095 | -0.1407 | -0.1494 | -0.1807 | 0.0000 | 1G | 0.3886 | -0.2596 | -0.6482 | -0.6516 | -1.0402 | -0.0008 |
| 14/02/2023 | 2S | 0.0397 | -0.0441 | -0.0838 | -0.1278 | -0.1675 | 0.0000 | 2G | 0.3539 | -0.0900 | -0.4439 | -0.1887 | -0.5426 | -0.0005 |
| 14/02/2023 | 3S | 0.0363 | -0.0427 | -0.0790 | -0.0785 | -0.1148 | 0.0000 | 3G | 0.3480 | -0.0266 | -0.3745 | -0.4753 | -0.8233 | -0.0003 |
| 14/02/2023 | 4S | 0.0391 | -0.0373 | -0.0764 | -0.0661 | -0.1052 | 0.0000 | 4G | 0.3682 | -0.2231 | -0.5913 | -0.3906 | -0.7588 | -0.0006 |
| 14/02/2023 | 5S | 0.0396 | -0.0437 | -0.0834 | -0.1080 | -0.1476 | 0.0000 | 5G | 0.4794 | 0.0847 | -0.3947 | -0.0843 | -0.5637 | -0.0008 |
| 14/02/2023 | 6S | 0.0306 | -0.0163 | -0.0469 | -0.0388 | -0.0694 | 0.0001 | 6G | 0.3592 | -0.3602 | -0.7195 | -0.7222 | -1.0814 | -0.0005 |
| 27/03/2023 | 1S | 0.0743 | -0.1175 | -0.1918 | -0.1564 | -0.2307 | 0.0001 | 1G | 0.7232 | -0.2518 | -0.9750 | -0.4468 | -1.1700 | -0.0001 |
| 27/03/2023 | 2S | 0.0608 | -0.1172 | -0.1780 | -0.1444 | -0.2053 | 0.0000 | 2G | 0.5115 | -0.4978 | -1.0093 | -0.9179 | -1.4293 | -0.0001 |
| 27/03/2023 | 3S | 0.0441 | -0.0918 | -0.1359 | -0.1248 | -0.1689 | 0.0000 | 3G | 0.6649 | -0.4186 | -1.0835 | -0.9347 | -1.5996 | 0.0009 |
| 27/03/2023 | 4S | 0.0968 | -0.0812 | -0.1780 | -0.1113 | -0.2081 | 0.0000 | 4G | 0.3303 | -0.1469 | -0.4772 | -0.4843 | -0.8146 | 0.0000 |
| 27/03/2023 | 5S | 0.0791 | -0.1201 | -0.1992 | -0.1689 | -0.2480 | 0.0000 | 5G | 0.4570 | -0.3097 | -0.7667 | -0.3915 | -0.8485 | -0.0002 |
| 27/03/2023 | 6S | 0.0811 | -0.0993 | -0.1804 | -0.1790 | -0.2602 | 0.0000 | 6G | 0.2522 | -0.4287 | -0.6809 | -0.3659 | -0.6181 | -0.0005 |
| 18/04/2023 | 1S | 0.1748 | -0.1385 | -0.3133 | -0.2634 | -0.4382 | 0.0000 | 1G | 0.8079 | -0.5293 | -1.3371 | -0.6661 | -1.4740 | 0.0000 |
| 18/04/2023 | 2S | 0.1563 | -0.1718 | -0.3281 | -0.2347 | -0.3910 | 0.0000 | 2G | 1.0177 | -1.0530 | -2.0707 | -1.3132 | -2.3310 | -0.0002 |
| 18/04/2023 | 3S | 0.1569 | -0.1331 | -0.2901 | -0.1924 | -0.3493 | 0.0000 | 3G | 1.2567 | -1.4837 | -2.7404 | -1.8335 | -3.0902 | 0.0012 |
| 18/04/2023 | 4S | 0.1678 | -0.0055 | -0.1733 | -0.1785 | -0.3463 | 0.0000 | 4G | 1.3802 | -0.3263 | -1.7065 | -0.7066 | -2.0867 | 0.0002 |
| 18/04/2023 | 5S | 0.1430 | -0.0700 | -0.2130 | -0.1713 | -0.3143 | 0.0000 | 5G |  |  |  |  |  |  |
| 18/04/2023 | 6S | 0.0918 | -0.1748 | -0.2666 | -0.2285 | -0.3203 | 0.0000 | 6G | 1.2677 | -1.2336 | -2.5014 | -1.6113 | -2.8791 | -0.0004 |
| 20/05/2023 | 1S | 0.2744 | -0.1049 | -0.3794 | -0.2067 | -0.4811 | 0.0001 | 1G | 2.1552 | -0.1205 | -2.2757 | -0.3715 | -2.5266 | -0.0008 |
| 20/05/2023 | 2S | 0.3131 | -0.0960 | -0.4091 | -0.1713 | -0.4844 | -0.0001 | 2G | 2.8930 | -0.1654 | -3.0584 | -0.6231 | -3.5161 | -0.0011 |
| 20/05/2023 | 3S | 0.2731 | -0.0325 | -0.3056 | -0.1298 | -0.4029 | 0.0000 | 3G | 2.6710 | -0.4768 | -3.1477 | -1.1447 | -3.8157 | -0.0004 |
| 20/05/2023 | 4S | 0.3222 | 0.0296 | -0.2925 | -0.0335 | -0.3557 | -0.0001 | 4G | 2.5001 | 0.1782 | -2.3219 | -0.8760 | -3.3761 | -0.0006 |
| 20/05/2023 | 5S | 0.3357 | -0.0248 | -0.3605 | -0.0819 | -0.4176 | 0.0000 | 5G |  |  |  |  |  |  |
| 20/05/2023 | 6S | 0.2774 | -0.1238 | -0.4013 | -0.2487 | -0.5261 | -0.0001 | 6G | 2.6481 | -1.3037 | -3.9518 | -1.9378 | -4.5859 | -0.0006 |
| 15/06/2023 | 1S | 0.1689 | 0.1435 | -0.0254 | 0.1450 | -0.0239 | -0.0002 | 1G | 1.4484 | -0.1476 | -1.5959 | -0.7322 | -2.1805 | -0.0039 |
| 15/06/2023 | 2S | 0.1725 | 0.1392 | -0.0333 | 0.1444 | -0.0281 | 0.0003 | 2G | 1.7672 | -0.8646 | -2.6319 | -1.4450 | -3.2123 | -0.0036 |
| 15/06/2023 | 3S | 0.1581 | 0.0813 | -0.0768 | 0.0715 | -0.0866 | -0.0003 | 3G | 2.2143 | -0.9803 | -3.1946 | -1.0768 | -3.2911 | -0.0029 |
| 15/06/2023 | 4S | 0.1844 | 0.1892 | 0.0048 | 0.1875 | 0.0031 | -0.0003 | 4G | 1.6465 | -0.9992 | -2.6458 | -1.4817 | -3.1283 | -0.0033 |
| 15/06/2023 | 5S | 0.3301 | 0.0711 | -0.2591 | 0.0141 | -0.3160 | 0.0011 | 5G | 1.6061 | -0.6122 | -2.2183 | -0.9577 | -2.5638 | -0.0039 |
| 15/06/2023 | 6S | 0.2682 | -0.0080 | -0.2762 | -0.0572 | -0.3254 | 0.0000 | 6G | 1.4080 | -0.7642 | -2.1721 | -1.3573 | -2.7652 | -0.0026 |
| 19/07/2023 | 1S | 0.1879 | -0.0350 | -0.2229 | -0.0876 | -0.2755 | -0.0001 | 1G | 1.7634 | 0.1603 | -1.6031 | -0.7613 | -2.5247 | -0.0010 |
| 19/07/2023 | 2S | 0.3093 | 0.0123 | -0.2970 | -0.0335 | -0.3428 | -0.0001 | 2G | 2.3222 | 0.5091 | -1.8130 | -0.1908 | -2.5130 | -0.0019 |
| 19/07/2023 | 3S | 0.1924 | -0.0250 | -0.2174 | -0.0597 | -0.2521 | 0.0000 | 3G | 1.6585 | -1.3601 | -3.0185 | -1.6286 | -3.2871 | -0.0011 |
| 19/07/2023 | 4S | 0.1798 | -0.0178 | -0.1977 | -0.0351 | -0.2150 | 0.0000 | 4G | 2.8770 | 0.6867 | -2.1903 | -0.9353 | -3.8123 | -0.0014 |
| 19/07/2023 | 5S | 0.1567 | -0.0282 | -0.1848 | -0.0619 | -0.2186 | 0.0000 | 5G | 1.6863 | -0.9246 | -2.6109 | -1.0444 | -2.7307 | -0.0005 |
| 19/07/2023 | 6S | 0.1202 | -0.0853 | -0.2055 | -0.1052 | -0.2253 | 0.0000 | 6G | 1.8024 | -1.2001 | -3.0025 | -1.6223 | -3.4247 | -0.0019 |
| 17/08/2023 | 1S | 0.1349 | -0.2003 | -0.3352 | -0.2370 | -0.3720 | 0.0000 | 1G | 1.8999 | -0.5201 | -2.4200 | -1.1238 | -3.0237 | -0.0012 |
| 17/08/2023 | 2S | 0.1718 | -0.1624 | -0.3342 | -0.2084 | -0.3802 | 0.0000 | 2G | 2.0385 | -1.7573 | -3.7958 | -1.9494 | -3.9879 | -0.0008 |
| 17/08/2023 | 3S | 0.1577 | -0.0839 | -0.2416 | -0.1541 | -0.3118 | 0.0001 | 3G | 1.7771 | -0.6389 | -2.4160 | -1.5324 | -3.3095 | -0.0009 |
| 17/08/2023 | 4S | 0.1430 | -0.1239 | -0.2669 | -0.1570 | -0.3000 | 0.0000 | 4G | 0.7498 | -0.6823 | -1.4322 | -1.1916 | -1.9414 | -0.0003 |
| 17/08/2023 | 5S | 0.1605 | -0.1171 | -0.2775 | -0.1257 | -0.2862 | 0.0000 | 5G | 1.3312 | -0.7641 | -2.0953 | -1.1765 | -2.5077 | -0.0008 |
| 17/08/2023 | 6S | 0.1257 | -0.1142 | -0.2399 | -0.1870 | -0.3127 | 0.0000 | 6G | 1.0685 | -1.6524 | -2.7209 | -2.4801 | -3.5485 | -0.0039 |
| 29/09/2023 | 1S | 0.0849 | -0.1820 | -0.2669 | -0.3227 | -0.4076 | -0.0001 | 1G | 0.8582 | -0.8792 | -1.7373 | -2.0734 | -2.9316 | 0.0000 |
| 29/09/2023 | 2S | 0.0734 | -0.2415 | -0.3149 | -0.2791 | -0.3525 | 0.0000 | 2G | 1.2674 | -1.7030 | -2.9704 | -2.1220 | -3.3895 | -0.0003 |
| 29/09/2023 | 3S | 0.0857 | -0.1461 | -0.2318 | -0.1453 | -0.2310 | 0.0001 | 3G | 1.0387 | -1.3850 | -2.4237 | -2.0907 | -3.1294 | -0.0003 |
| 29/09/2023 | 4S | 0.0719 | -0.1444 | -0.2164 | -0.1756 | -0.2475 | -0.0003 | 4G | 1.1123 | -1.3386 | -2.4509 | -1.5528 | -2.6651 | -0.0003 |
| 29/09/2023 | 5S | 0.0674 | -0.1400 | -0.2075 | -0.1608 | -0.2282 | -0.0001 | 5G | 0.8453 | -1.1036 | -1.9489 | -2.2886 | -3.1338 | 0.0002 |
| 29/09/2023 | 6S | 0.0512 | -0.1741 | -0.2253 | -0.2295 | -0.2807 | 0.0002 | 6G | 1.3608 | -0.9409 | -2.3016 | -2.2320 | -3.5927 | -0.0002 |
| 22/10/2023 | 1S | 0.0732 | -0.1938 | -0.2670 | -0.3180 | -0.3912 | 0.0001 | 1G | 0.6699 | -0.7604 | -1.4303 | -1.2508 | -1.9207 | -0.0003 |
| 22/10/2023 | 2S | 0.0454 | -0.1609 | -0.2063 | -0.2027 | -0.2481 | -0.0010 | 2G | 0.8653 | -0.2321 | -1.0974 | -0.5496 | -1.4149 | -0.0005 |
| 22/10/2023 | 3S | 0.0434 | -0.1808 | -0.2243 | -0.2055 | -0.2489 | 0.0002 | 3G | 0.6770 | -0.2779 | -0.9549 | -0.5781 | -1.2551 | 0.0000 |
| 22/10/2023 | 4S | 0.0654 | -0.2545 | -0.3200 | -0.2864 | -0.3519 | 0.0002 | 4G | 0.9890 | 0.1407 | -0.8483 | -0.3570 | -1.3460 | -0.0003 |
| 22/10/2023 | 5S | 0.0457 | -0.1399 | -0.1857 | -0.1357 | -0.1814 | 0.0002 | 5G | 0.6331 | -0.1806 | -0.8136 | -0.4962 | -1.1292 | 0.0036 |
| 22/10/2023 | 6S | 0.0514 | -0.0748 | -0.1262 | -0.1347 | -0.1861 | 0.0000 | 6G | 0.8299 | -0.6805 | -1.5103 | -1.5902 | -2.4200 | -0.0002 |
| 17/11/2023 | 1S | 0.0008 | -0.1030 | -0.1038 | -0.1103 | -0.1111 | 0.0003 | 1G | 0.2912 | -1.1400 | -1.4312 | -1.8508 | -2.1420 | 0.0005 |
| 17/11/2023 | 2S | 0.0119 | -0.1320 | -0.1439 | -0.1474 | -0.1593 | 0.0000 | 2G | 0.4110 | -0.3828 | -0.7938 | -0.8287 | -1.2398 | 0.0001 |
| 17/11/2023 | 3S | 0.0031 | -0.0331 | -0.0362 | -0.0331 | -0.0362 | 0.0000 | 3G | 0.2881 | -0.7895 | -1.0777 | -0.8912 | -1.1793 | 0.0007 |
| 17/11/2023 | 4S | 0.0136 | -0.1487 | -0.1622 | -0.1514 | -0.1649 | 0.0001 | 4G | 0.4657 | -1.1487 | -1.6143 | -1.6111 | -2.0768 | 0.0005 |
| 17/11/2023 | 5S | 0.0044 | -0.0998 | -0.1041 | -0.1151 | -0.1195 | 0.0000 | 5G | 0.3363 | -0.6537 | -0.9900 | -0.8695 | -1.2058 | 0.0008 |
| 17/11/2023 | 6S | 0.0121 | -0.1643 | -0.1764 | -0.1856 | -0.1977 | 0.0002 | 6G | 0.4943 | -0.4969 | -0.9913 | -0.8667 | -1.3610 | 0.0000 |
| 13/12/2023 | 1S | 0.0026 | -0.0469 | -0.0495 | -0.0781 | -0.0807 | 0.0000 | 1G | 0.2769 | -0.4677 | -0.7445 | -0.6185 | -0.8954 | 0.0003 |
| 13/12/2023 | 2S | 0.0214 | -0.0529 | -0.0743 | -0.1057 | -0.1271 | 0.0002 | 2G | 0.2105 | -0.1543 | -0.3648 | -0.1275 | -0.3381 | -0.0001 |
| 13/12/2023 | 3S | 0.0039 | -0.0429 | -0.0467 | -0.0483 | -0.0522 | -0.0001 | 3G | 0.1743 | -0.4226 | -0.5969 | -0.4963 | -0.6706 | 0.0014 |
| 13/12/2023 | 4S | 0.0164 | -0.0856 | -0.1020 | -0.1251 | -0.1415 | 0.0000 | 4G | 0.2388 | -0.2037 | -0.4426 | -0.2012 | -0.4400 | 0.0002 |
| 13/12/2023 | 5S | 0.0050 | -0.0777 | -0.0827 | -0.1035 | -0.1084 | 0.0003 | 5G | 0.2210 | -0.5027 | -0.7237 | -0.5584 | -0.7795 | 0.0012 |
| 13/12/2023 | 6S | 0.0116 | -0.0632 | -0.0748 | -0.0930 | -0.1045 | 0.0000 | 6G | 0.2801 | -0.3941 | -0.6741 | -0.5428 | -0.8228 | 0.0002 |
| 06/01/2024 | 1S | 0.0089 | -0.0173 | -0.0262 | -0.0236 | -0.0325 | -0.0001 | 1G | 0.1774 | -0.5363 | -0.7138 | -0.6501 | -0.8275 | 0.0005 |
| 06/01/2024 | 2S | 0.0221 | -0.0328 | -0.0549 | -0.0437 | -0.0658 | 0.0008 | 2G | 0.1834 | -0.2191 | -0.4026 | -0.2818 | -0.4652 | 0.0010 |
| 06/01/2024 | 3S | 0.0090 | -0.0030 | -0.0121 | -0.0132 | -0.0222 | 0.0002 | 3G | 0.1621 | -0.4406 | -0.6027 | -0.5718 | -0.7339 | 0.0016 |
| 06/01/2024 | 4S | 0.0051 | -0.0468 | -0.0519 | -0.0638 | -0.0689 | 0.0000 | 4G | 0.1756 | -0.3399 | -0.5154 | -0.4944 | -0.6700 | 0.0009 |
| 06/01/2024 | 5S | 0.0093 | -0.0238 | -0.0331 | -0.0314 | -0.0407 | 0.0011 | 5G | 0.1529 | -0.2239 | -0.3768 | -0.4015 | -0.5544 | 0.0011 |
| 06/01/2024 | 6S | 0.0091 | -0.0352 | -0.0442 | -0.0388 | -0.0478 | 0.0000 | 6G | 0.2242 | -0.3764 | -0.6006 | -0.4080 | -0.6322 | -0.0004 |

**Companion Planting: Little Woolden Moss. Restoration plots (Vegetated)**

| 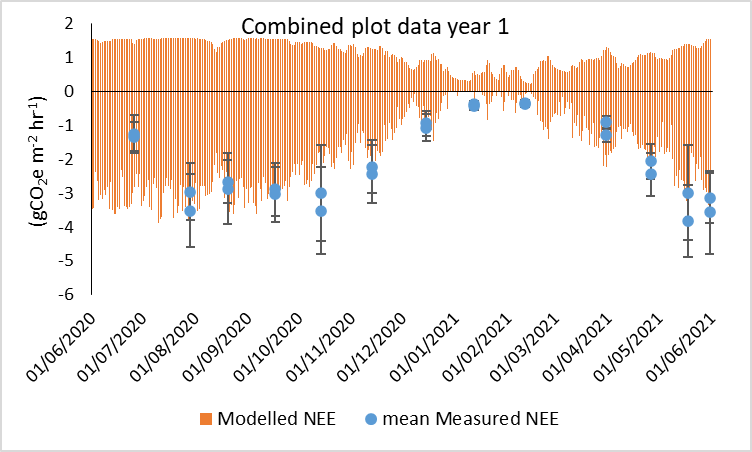 | 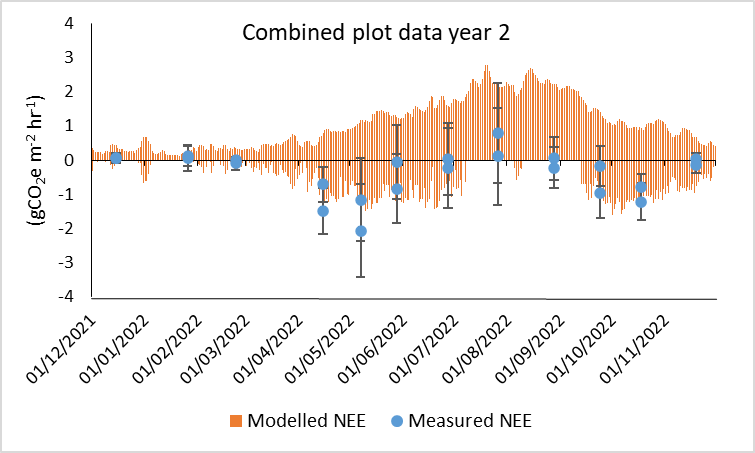 | 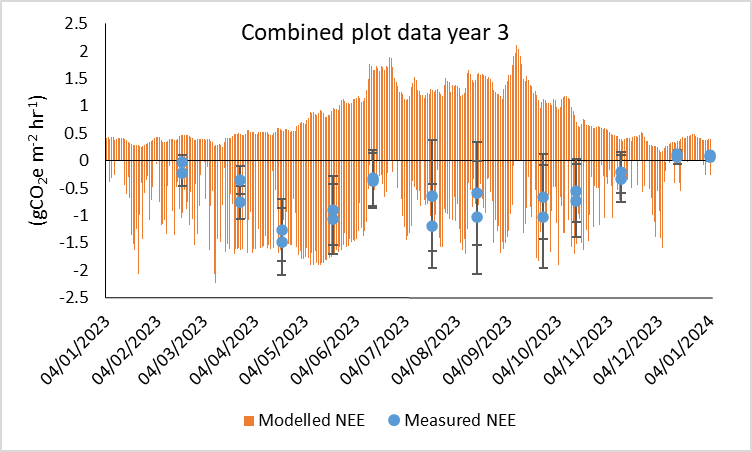 |
| --- | --- | --- |
| 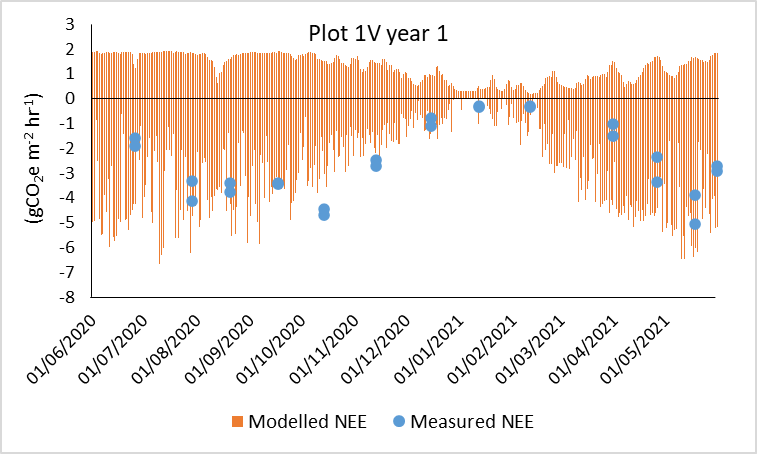 | 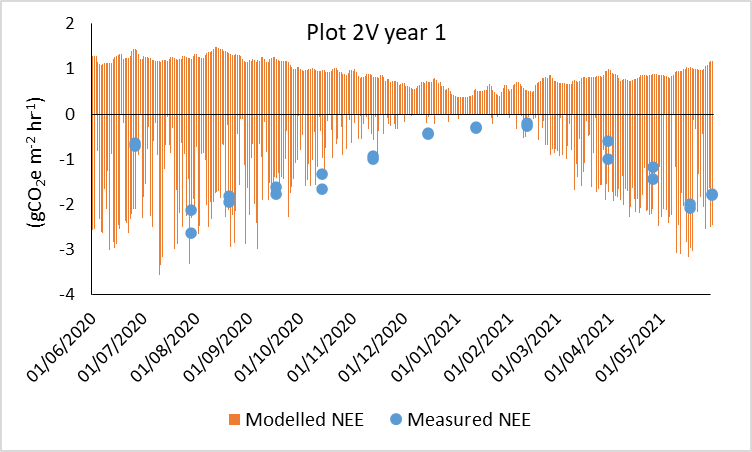 | 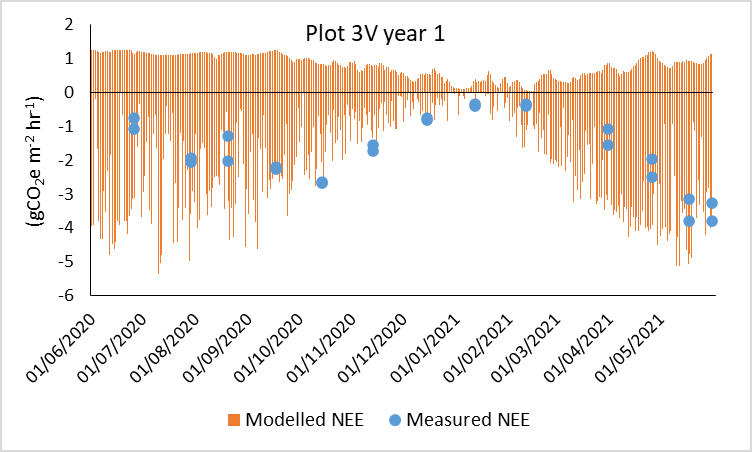 |
| 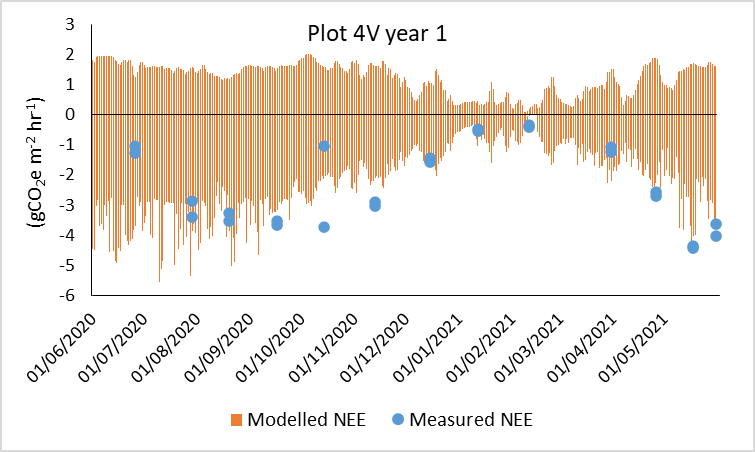 | 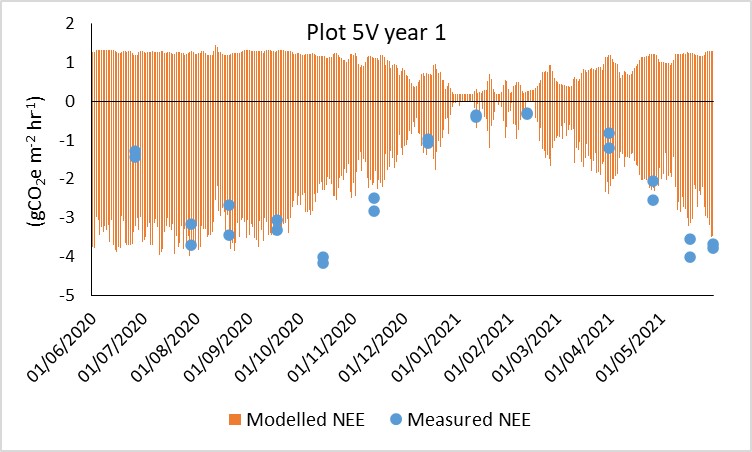 | 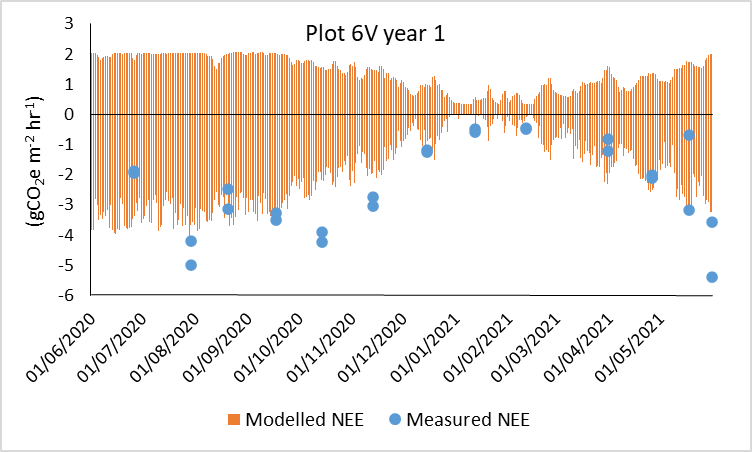 |
|  |  |  |
| 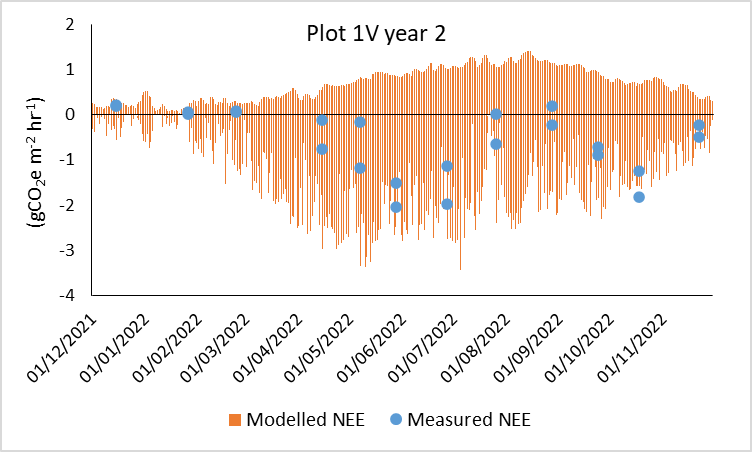 | 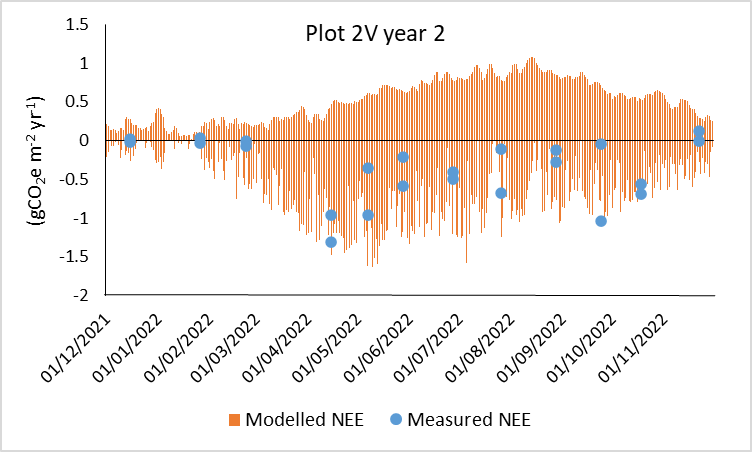 | 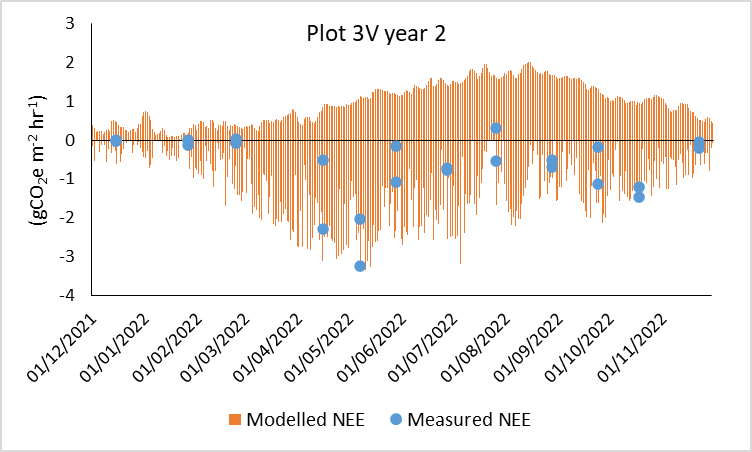 |
| 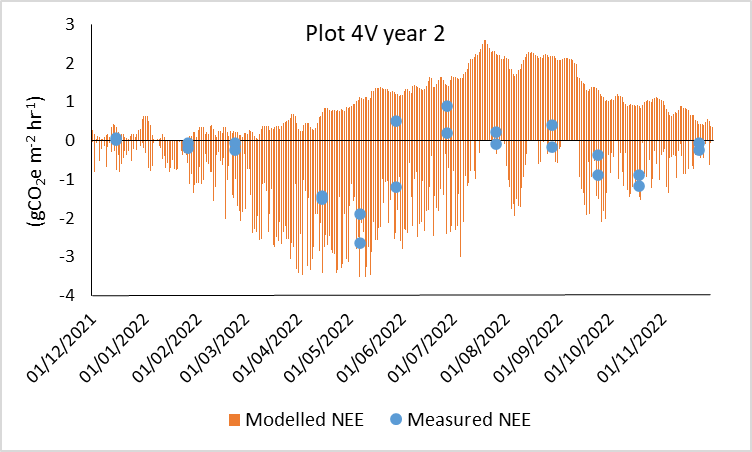 | 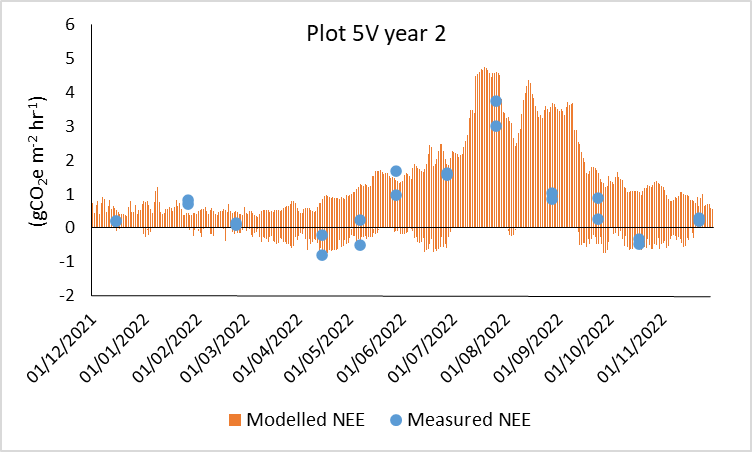 | 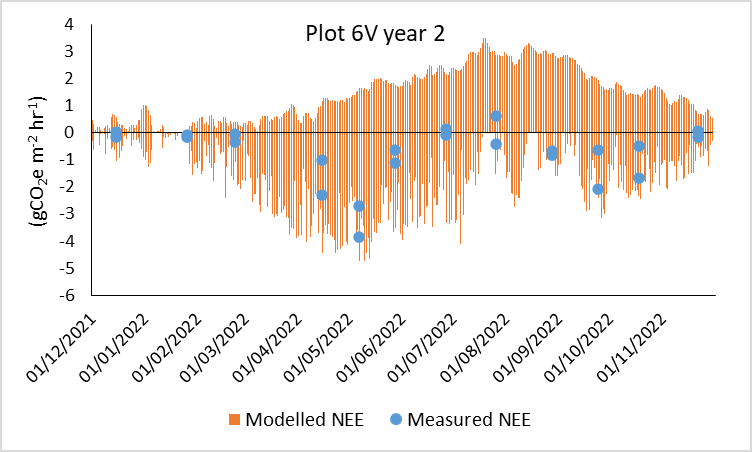 |
|  |  |  |

| 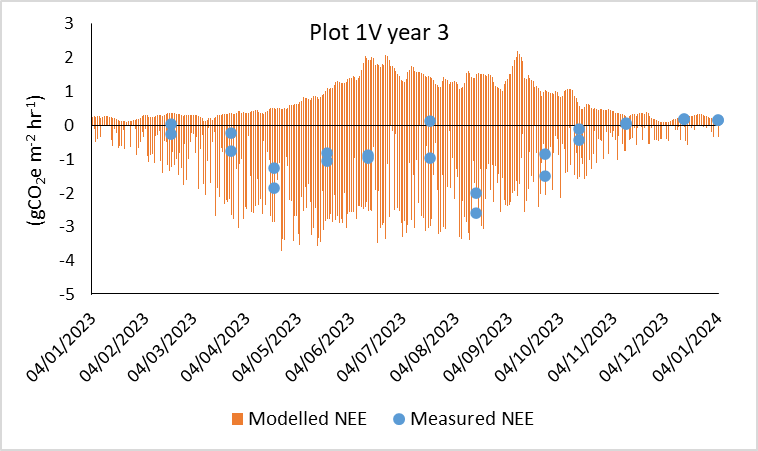 | 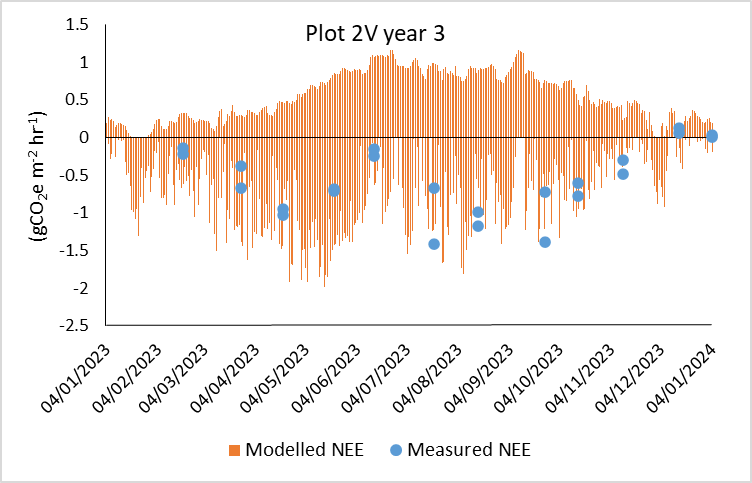 | 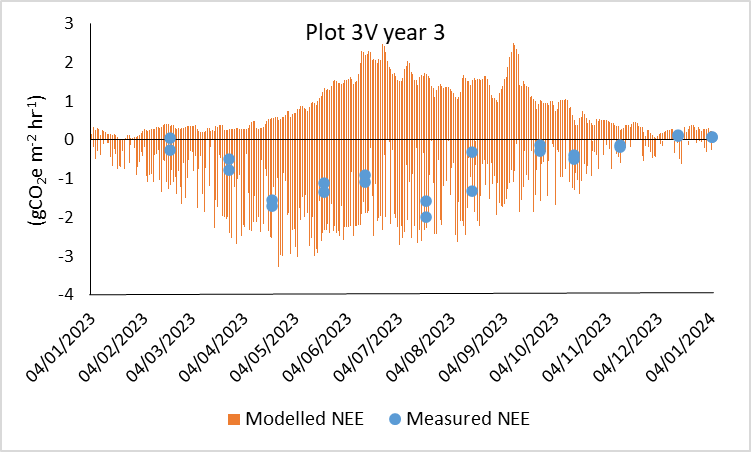 |
| --- | --- | --- |
| 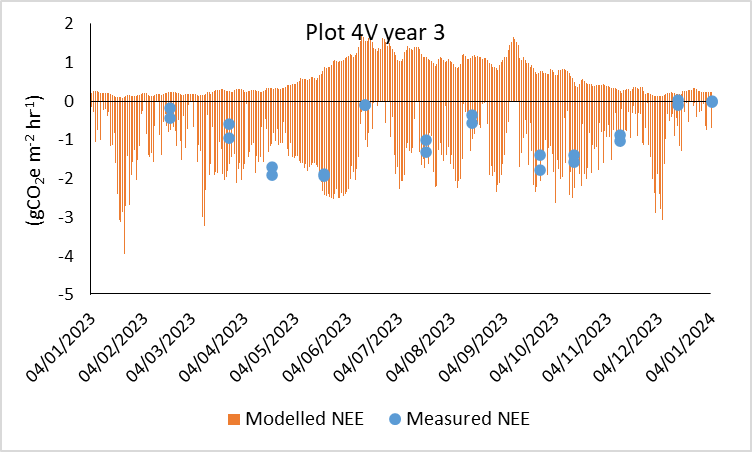 | 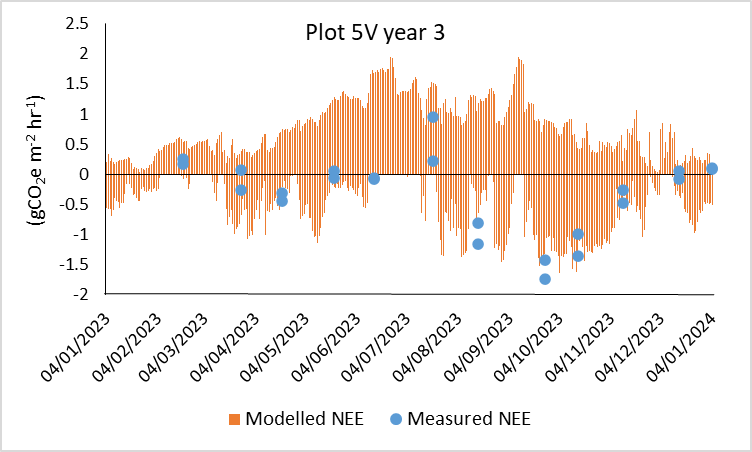 | 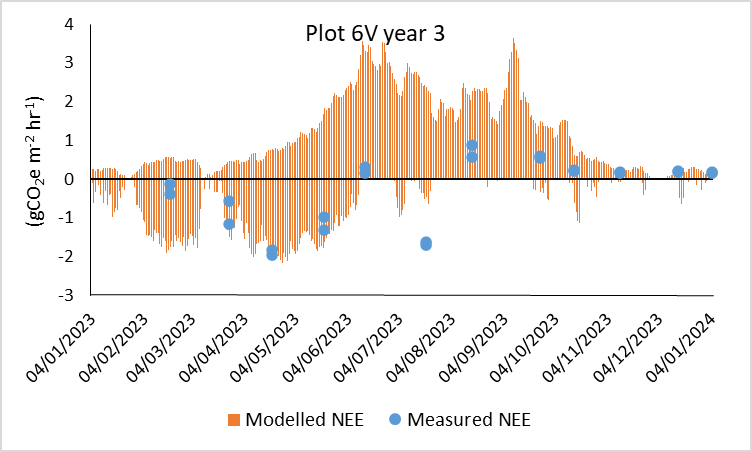 |

**Companion Planting: Little Woolden Moss. Control plots (Bare)**

| 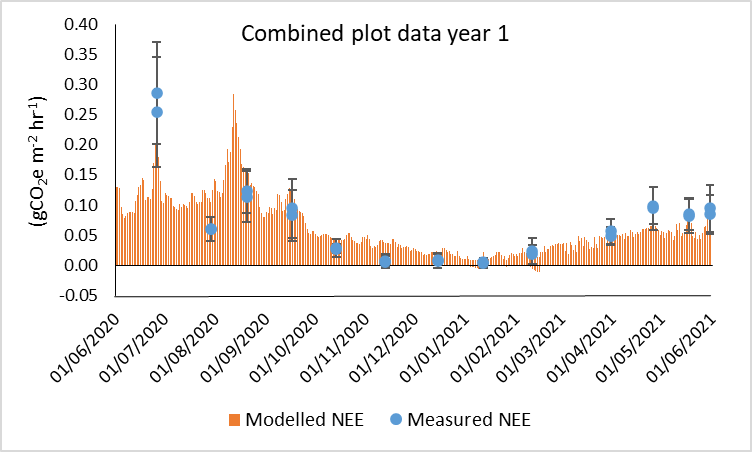 | 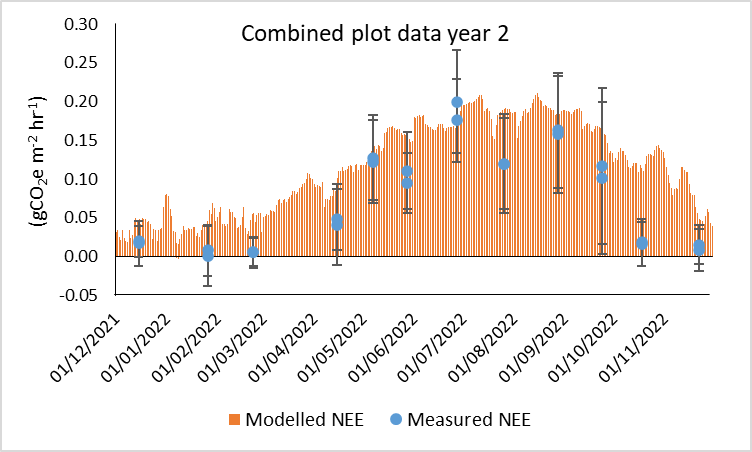 | 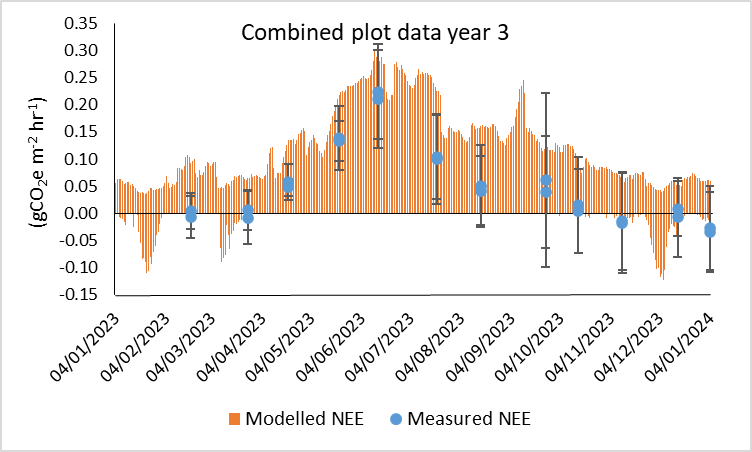 |
| --- | --- | --- |
| 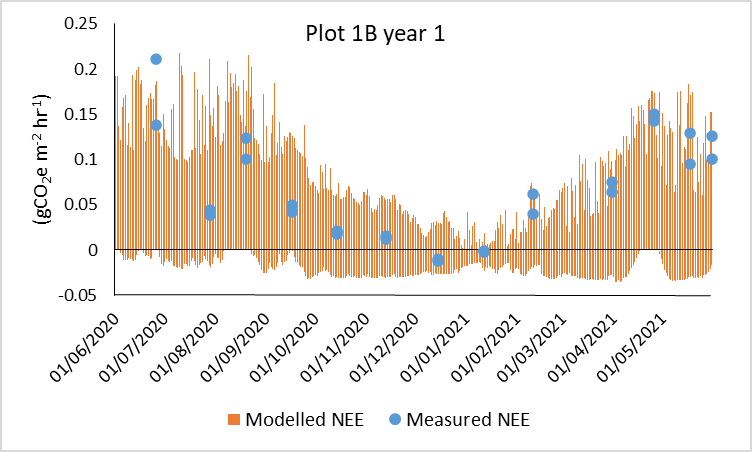 | 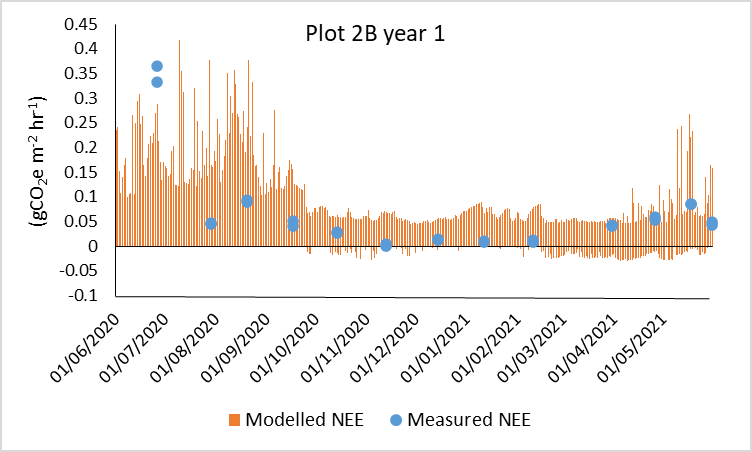 | 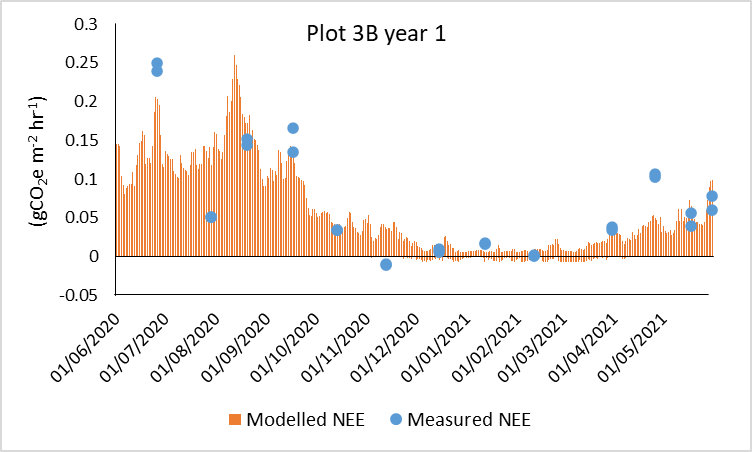 |
| 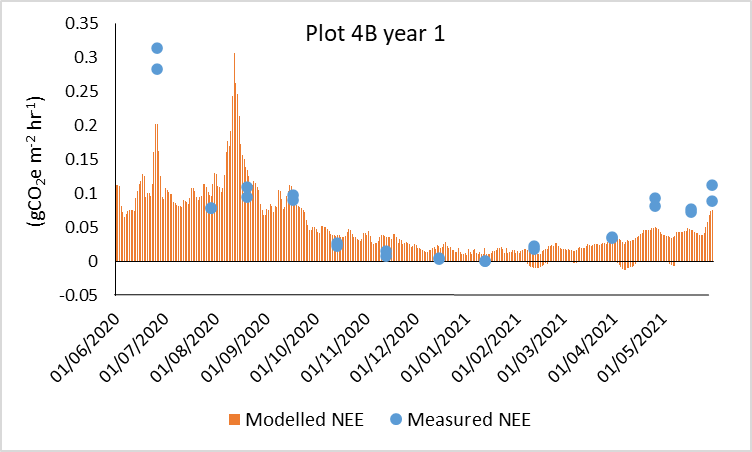 | 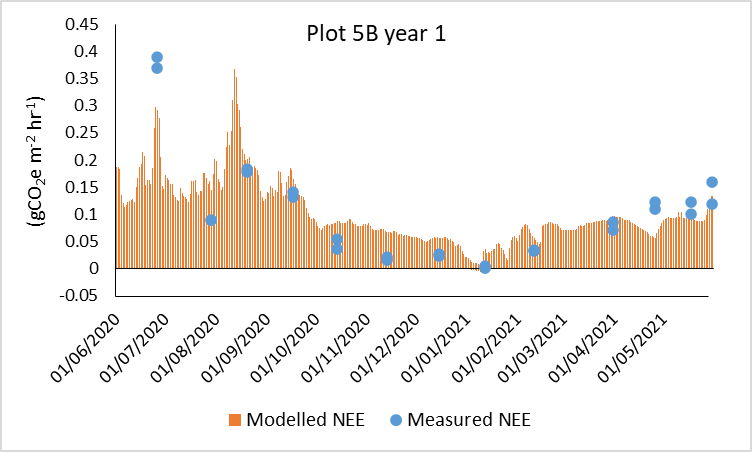 | 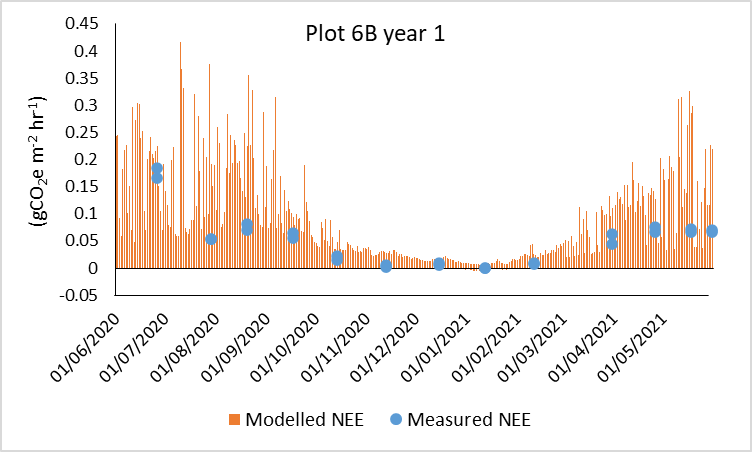 |

| 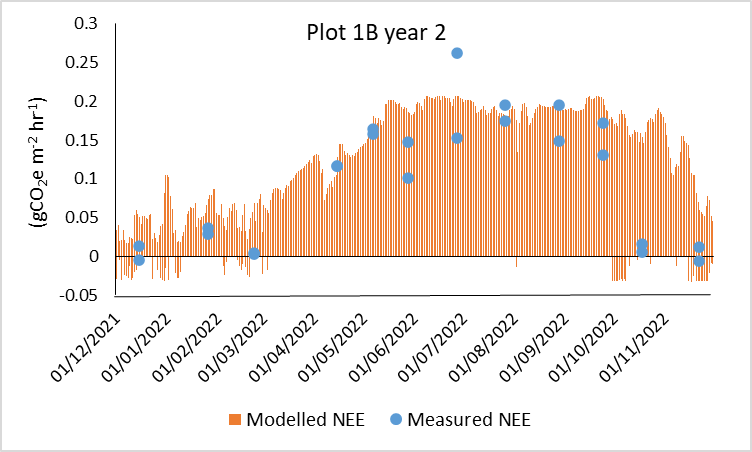 | 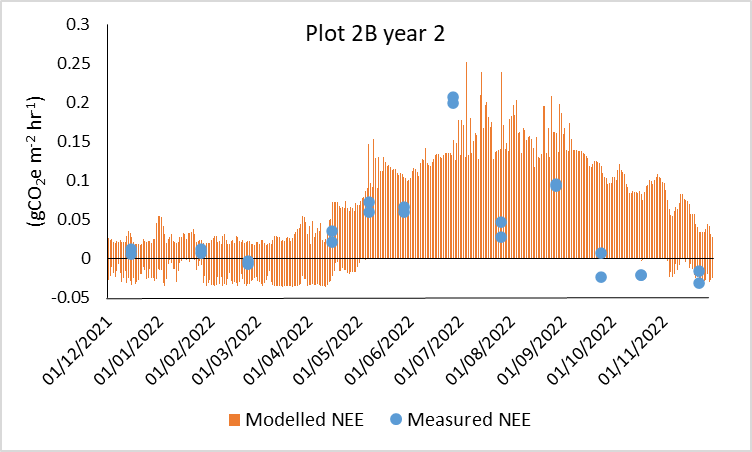 | 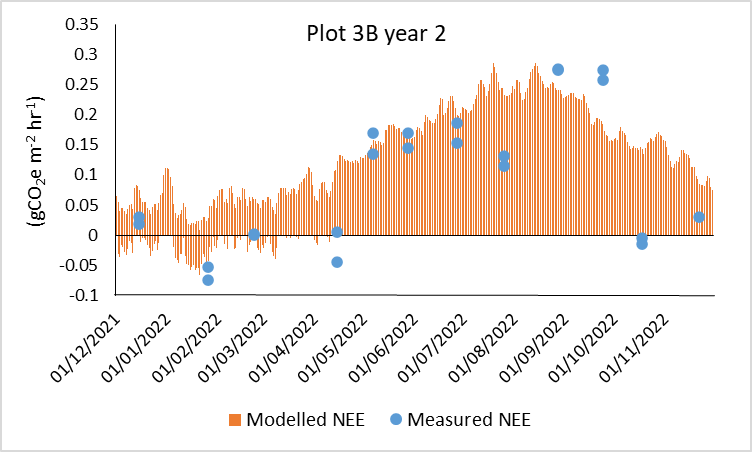 |
| --- | --- | --- |
| 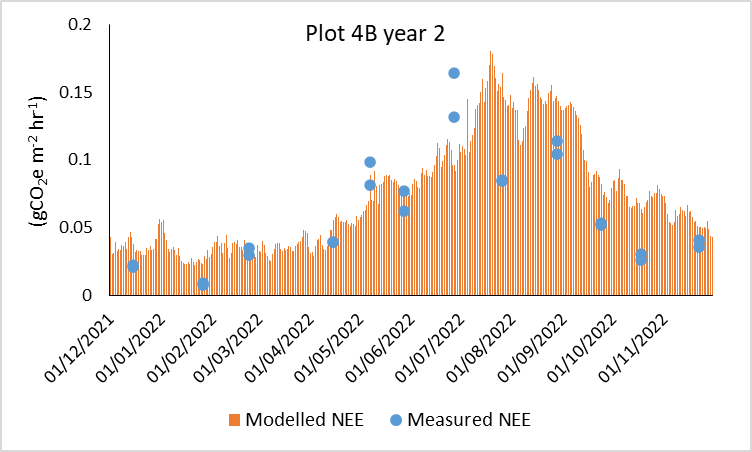 | 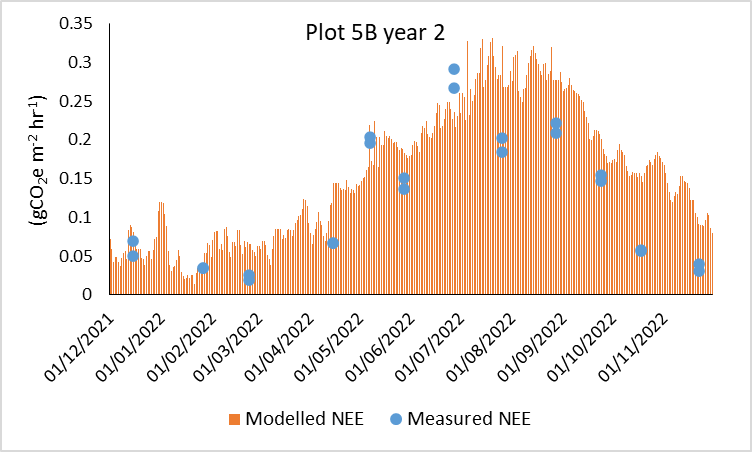 | 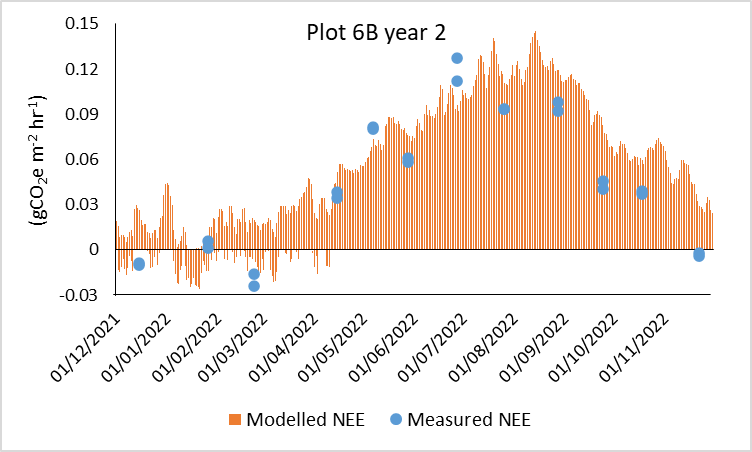 |

| 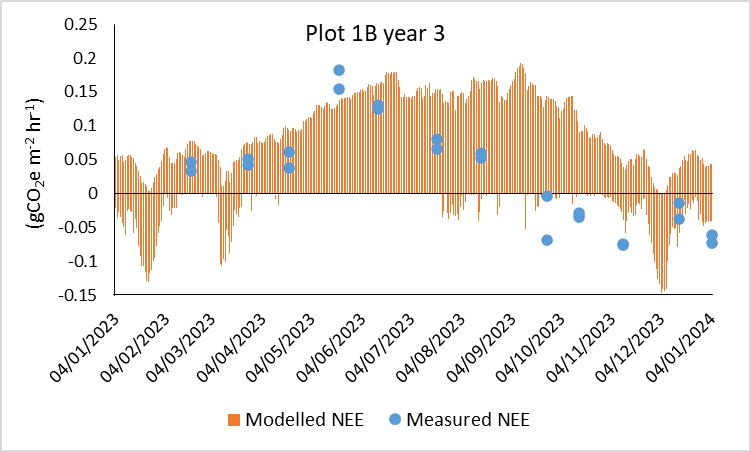 | 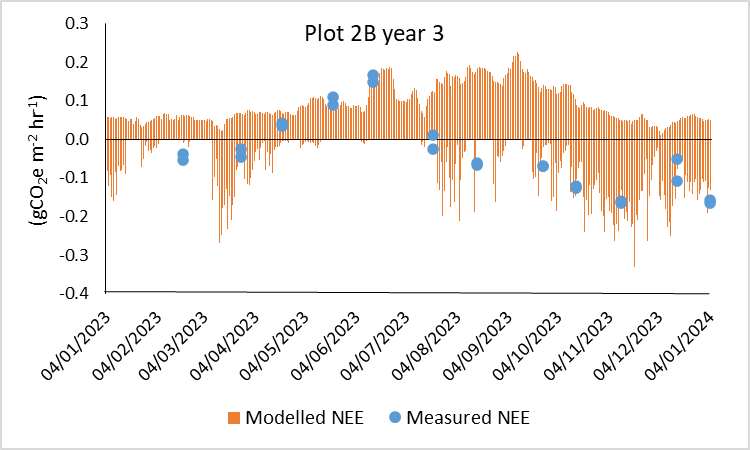 | 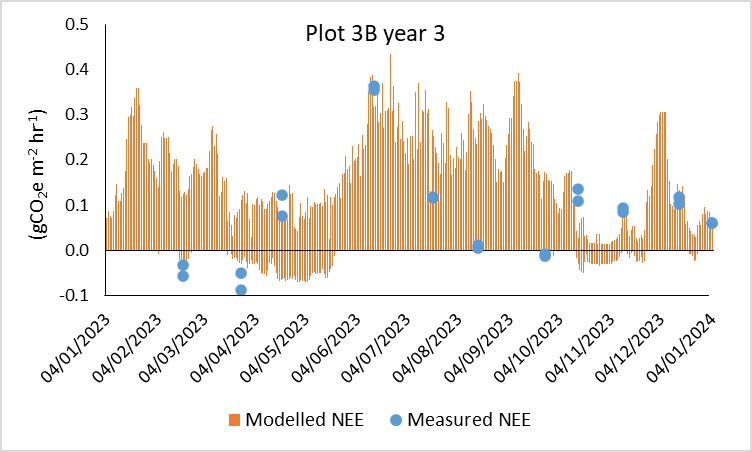 |
| --- | --- | --- |
| 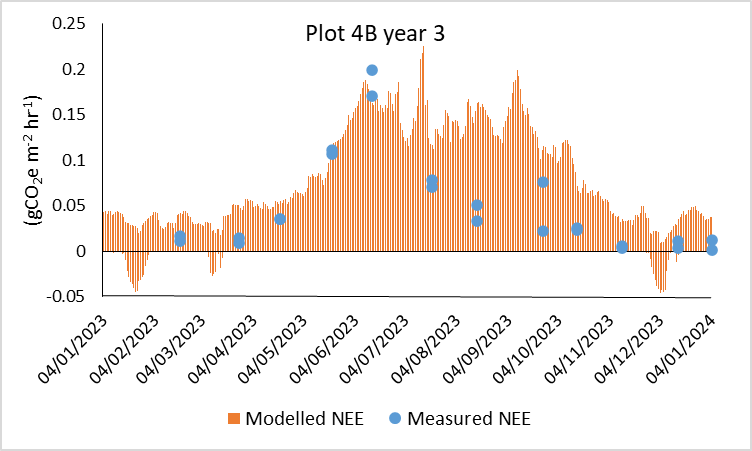 | 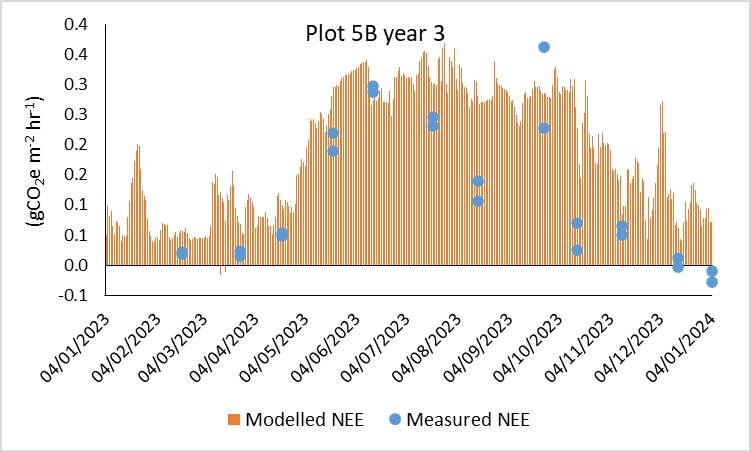 | 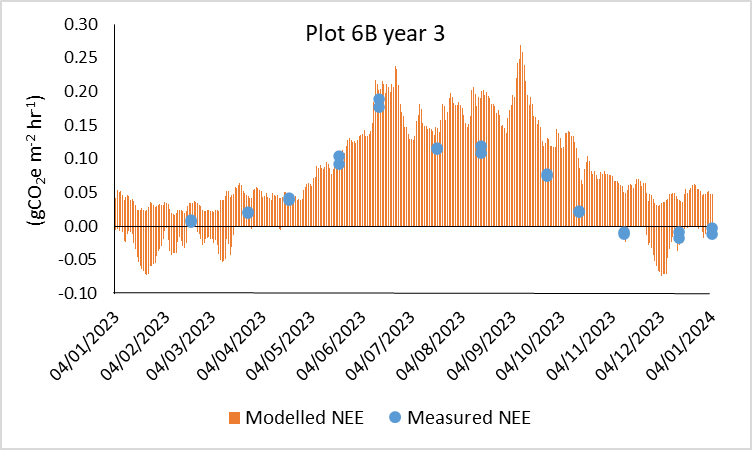 |

**Carbon Farm, Winmarleigh Moss. Restoration plots (Sphagnum-planted)**

| 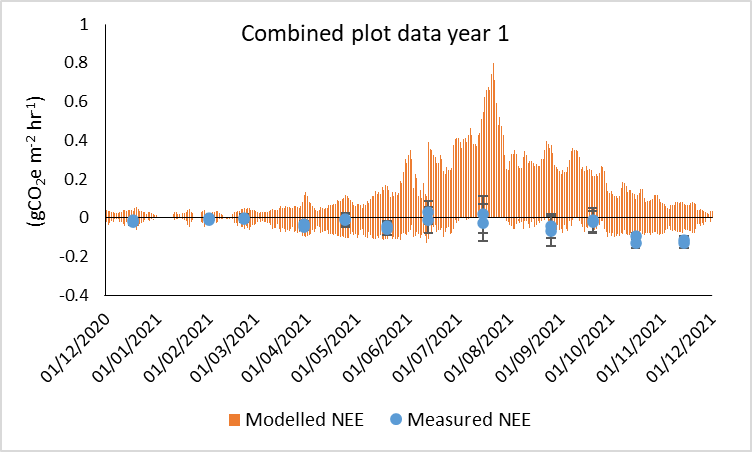 | 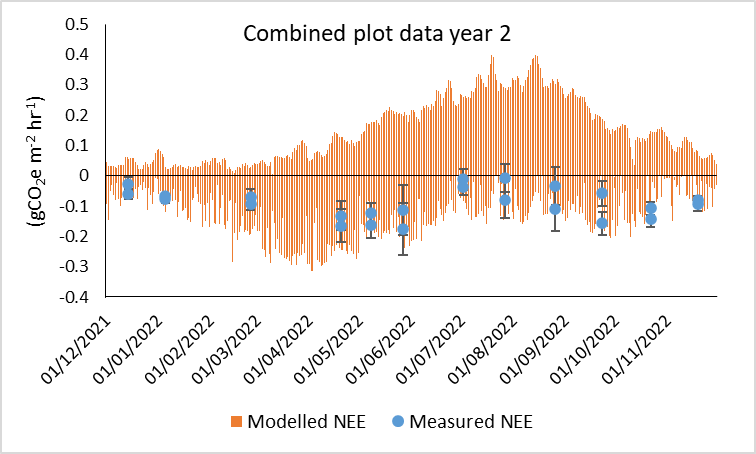 | 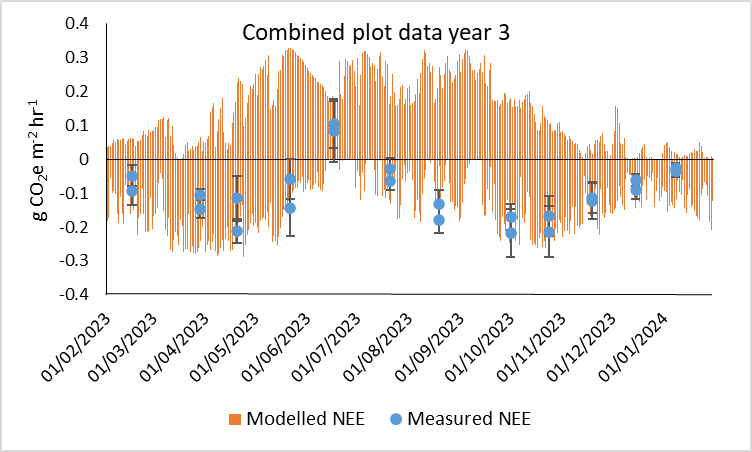 |
| --- | --- | --- |
| 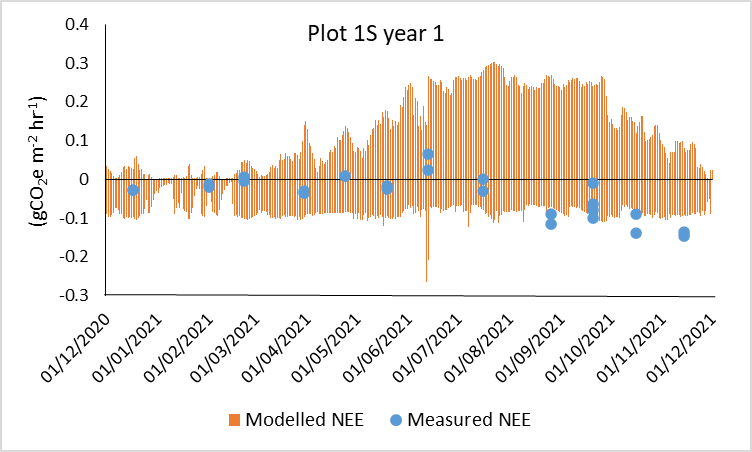 | 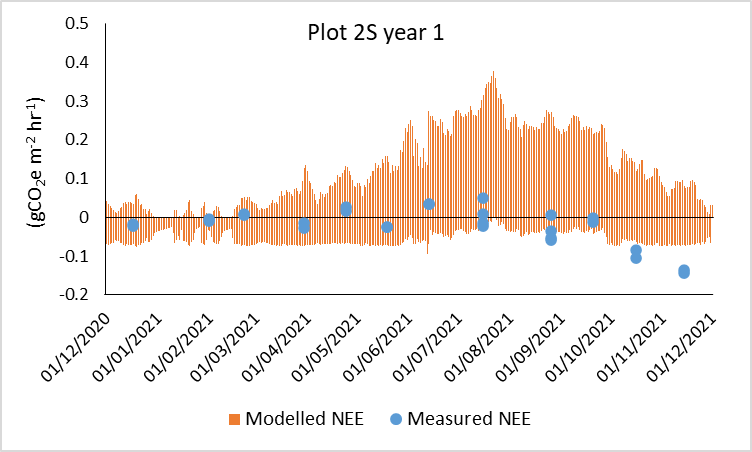 | 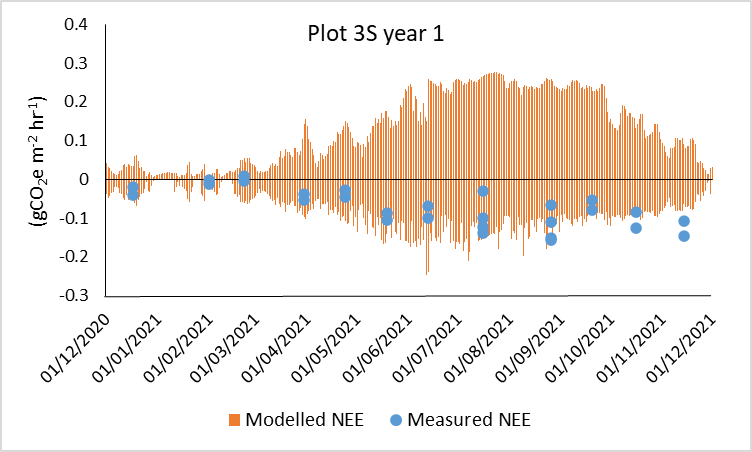 |
| 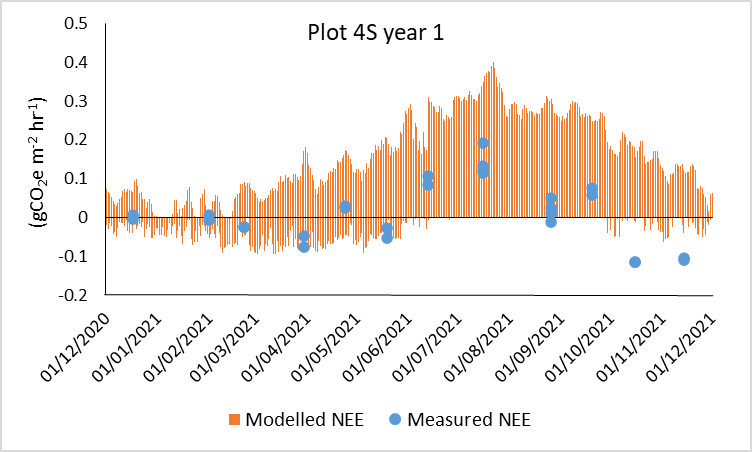 | 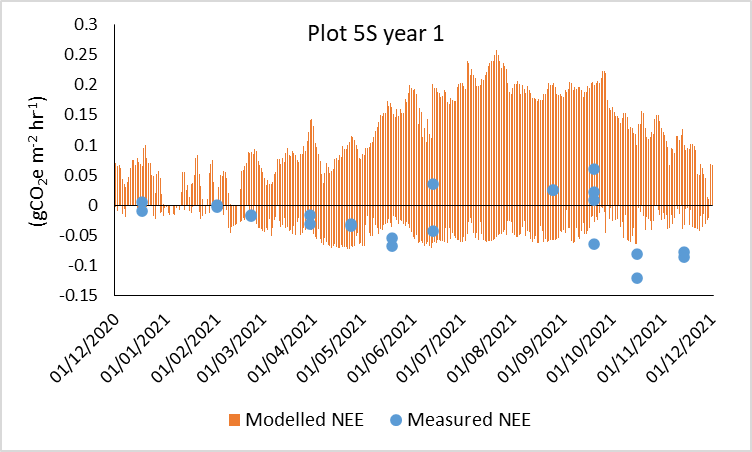 | 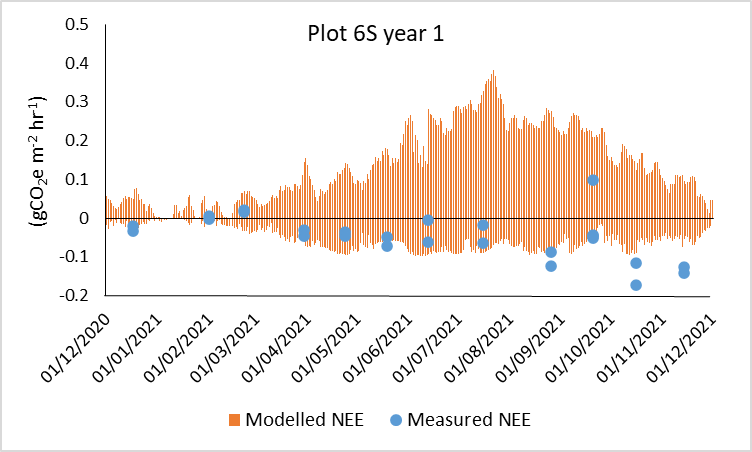 |

| 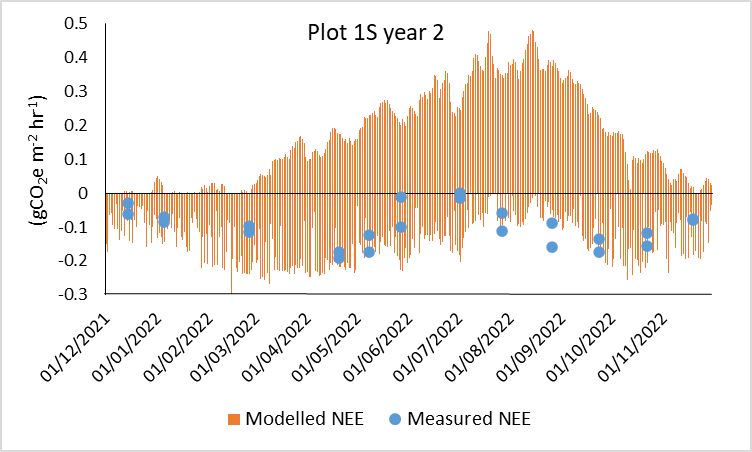 | 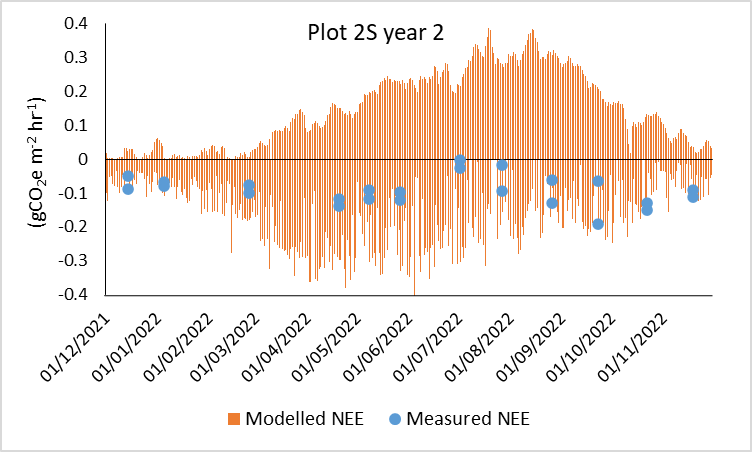 | 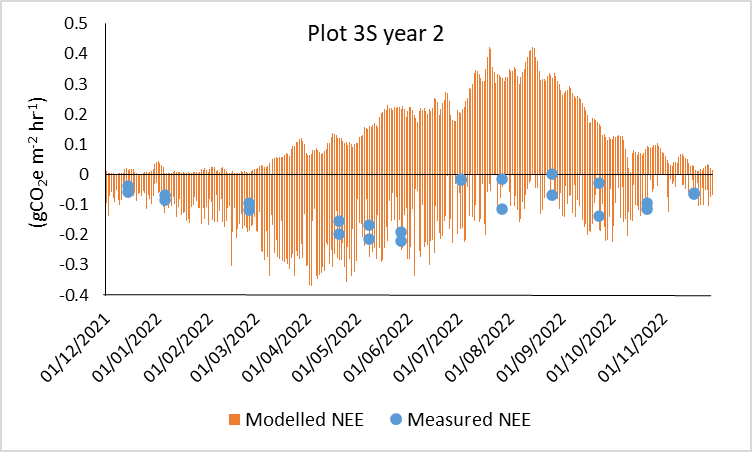 |
| --- | --- | --- |
| 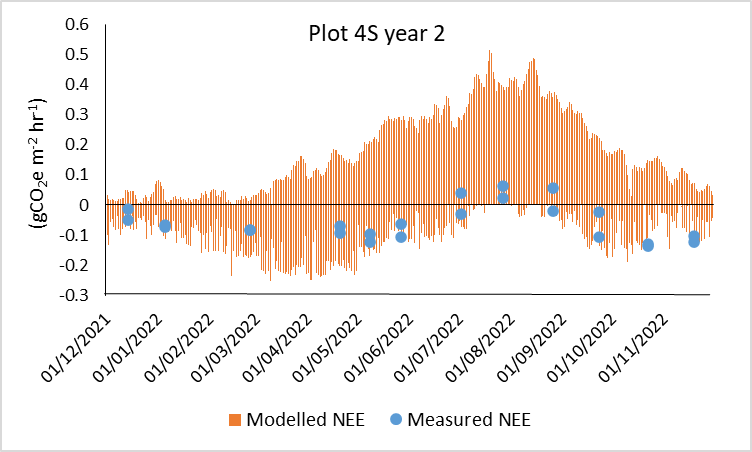 | 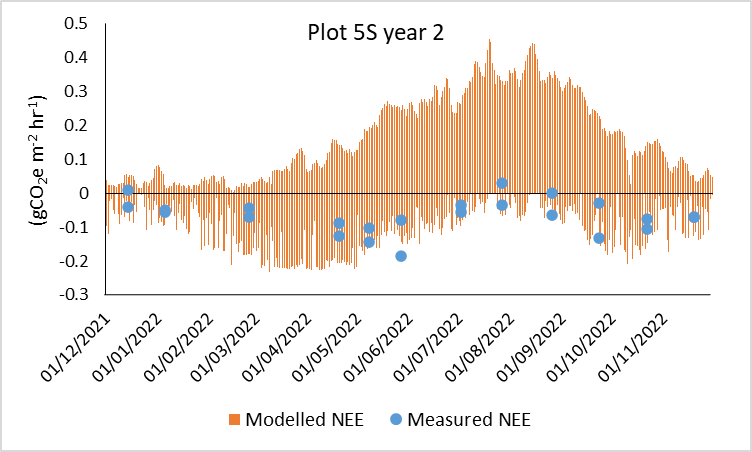 | 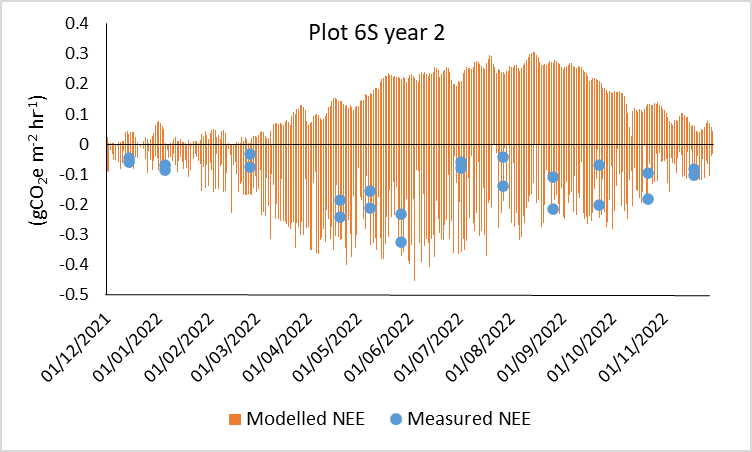 |

| 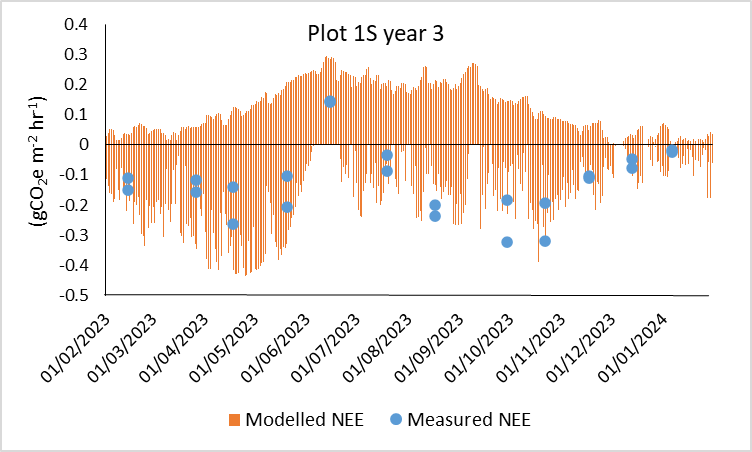 | 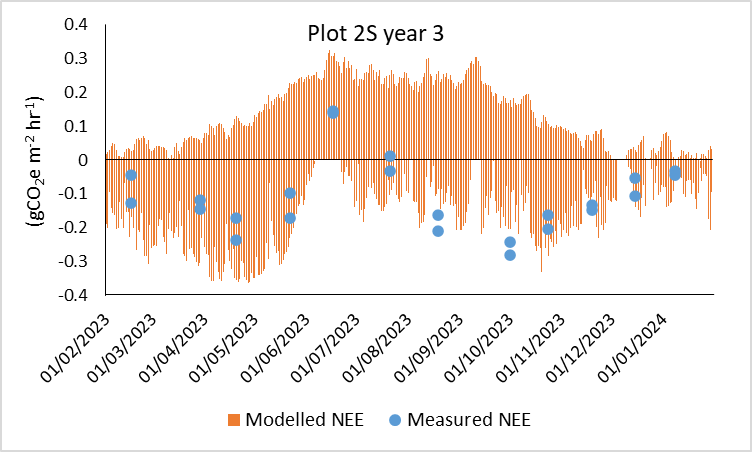 | 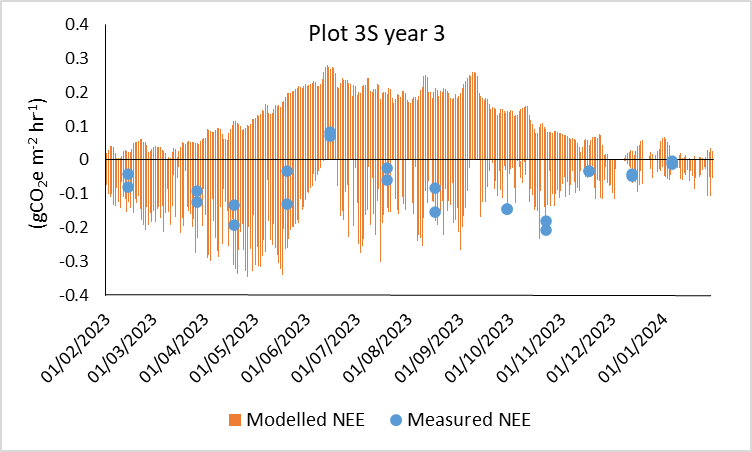 |
| --- | --- | --- |
| 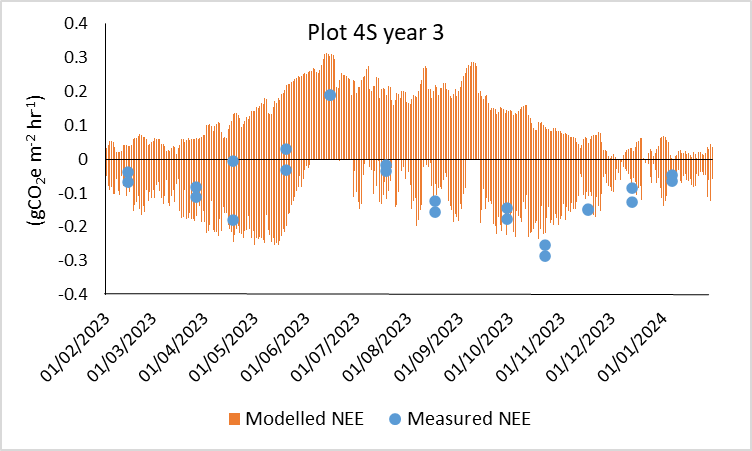 | 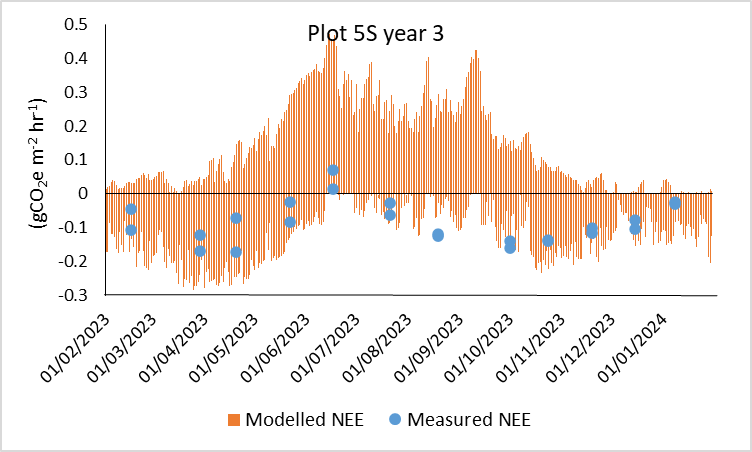 | 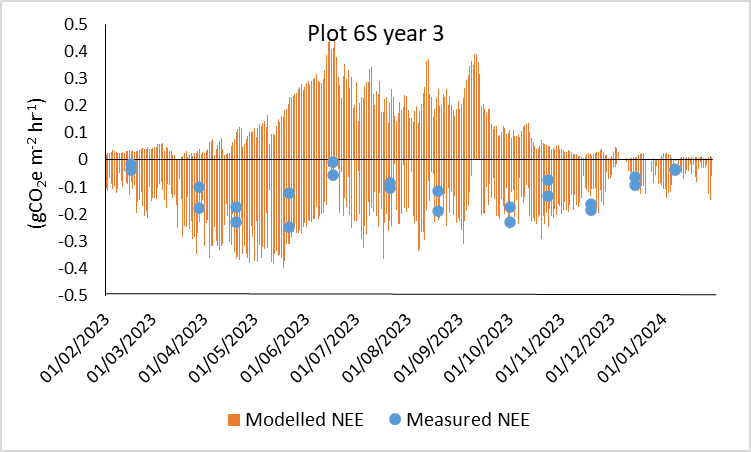 |

**Carbon Farm, Winmarleigh Moss. Control plots (Grazed Pasture)**

| 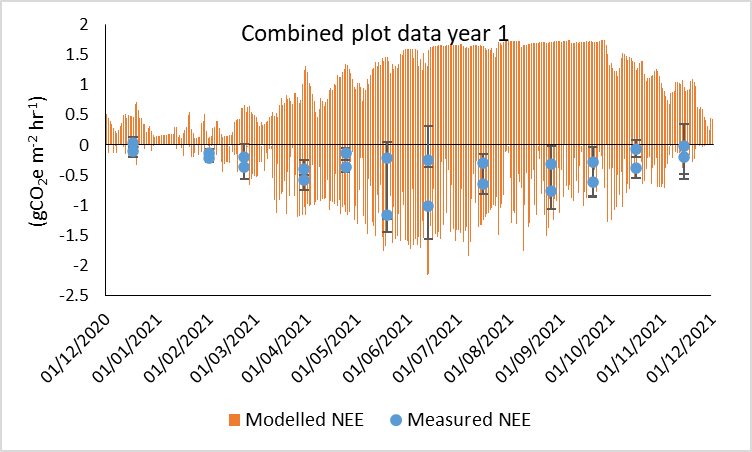 | 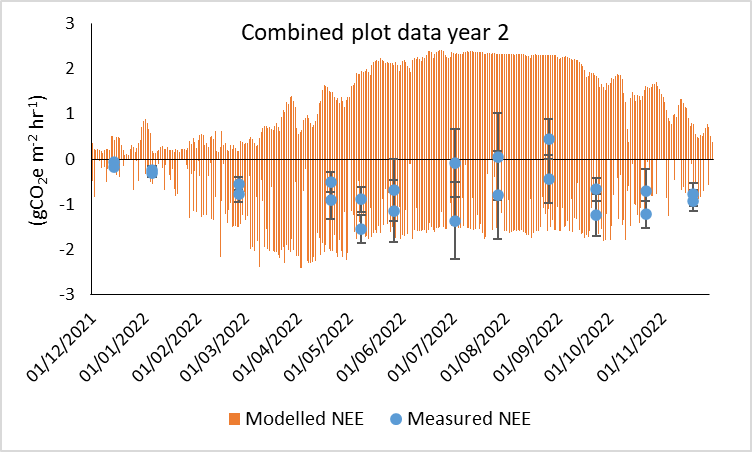 | 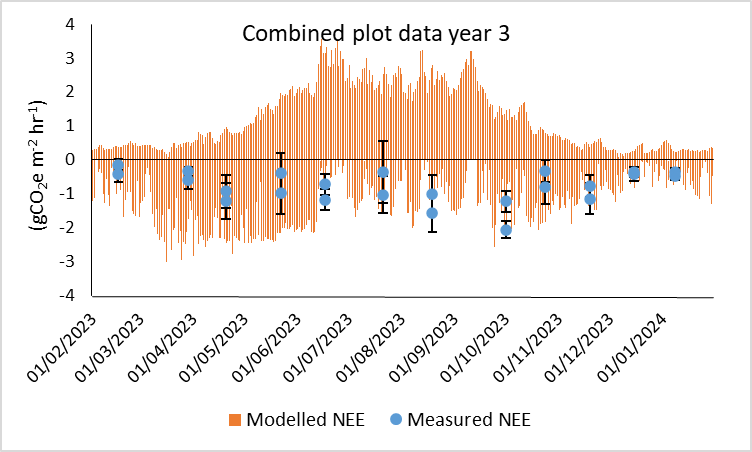 |
| --- | --- | --- |
| 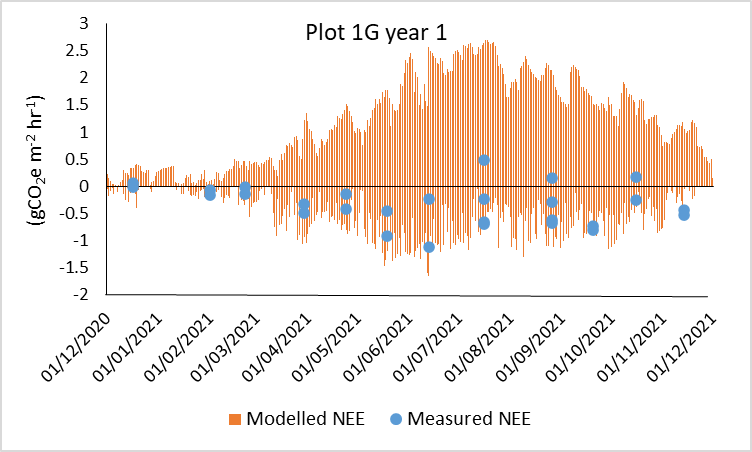 | 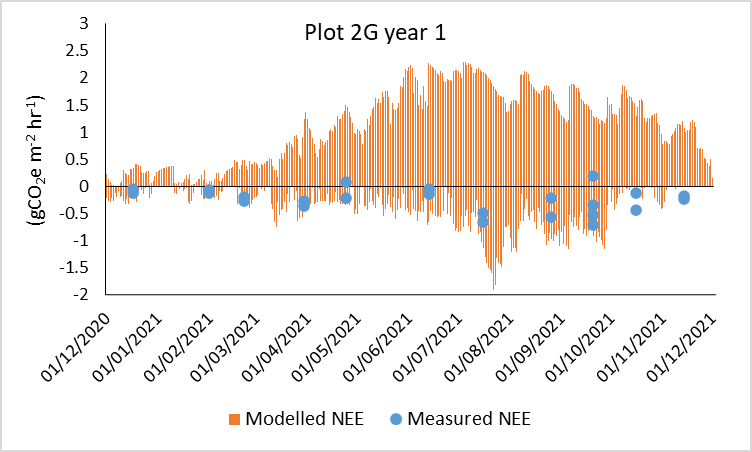 | 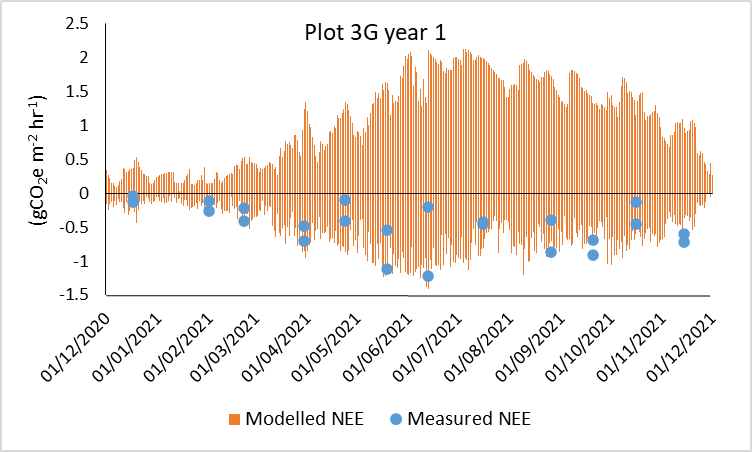 |
| 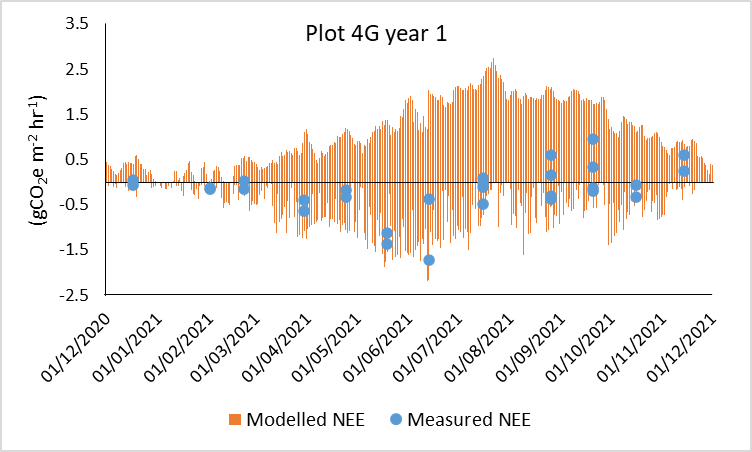 | 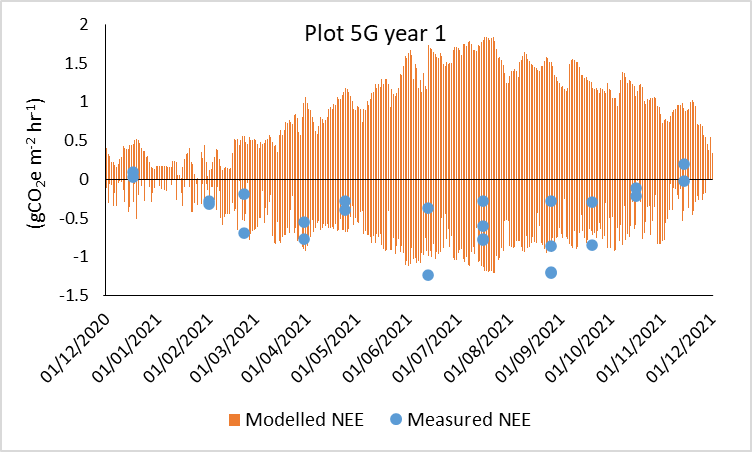 | 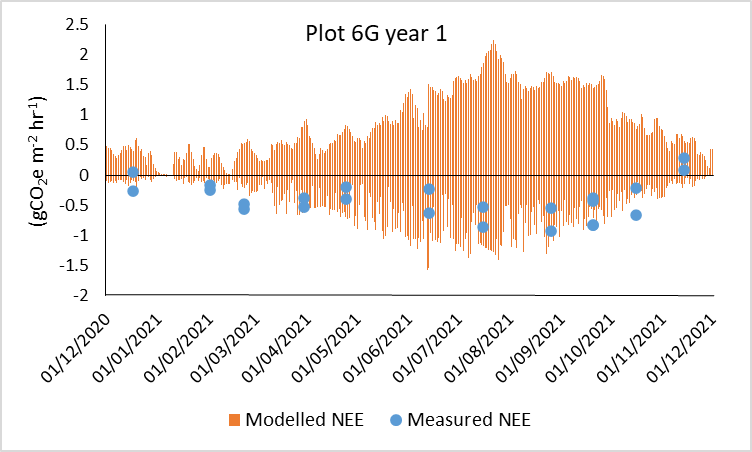 |

| 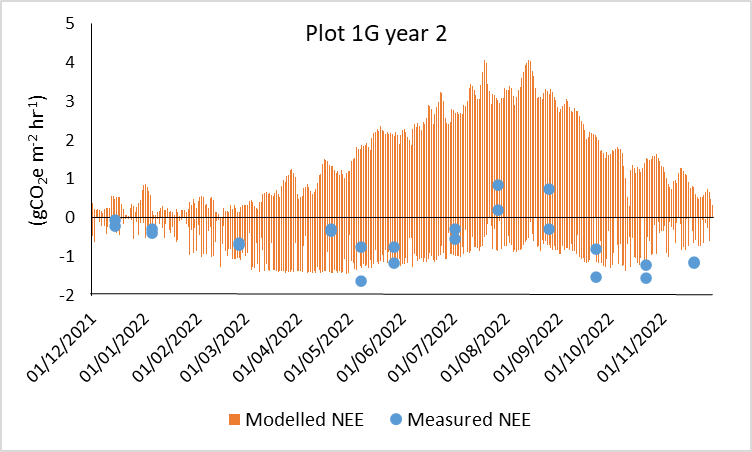 | 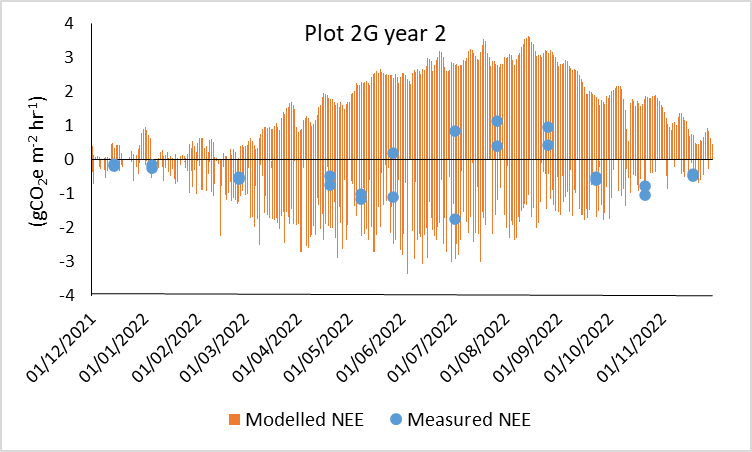 | 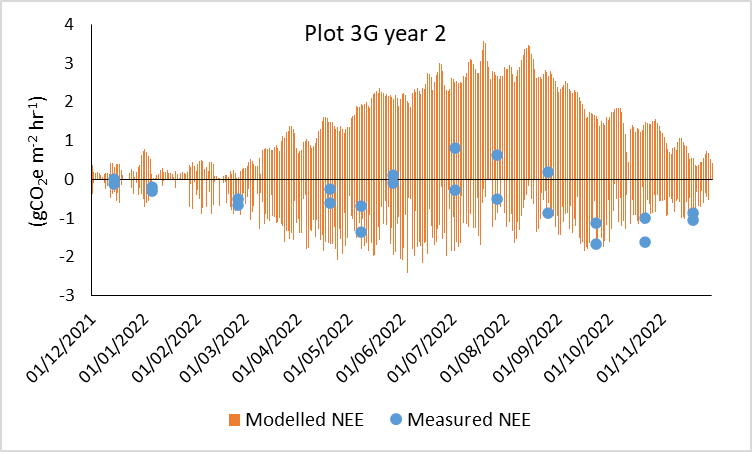 |
| --- | --- | --- |
| 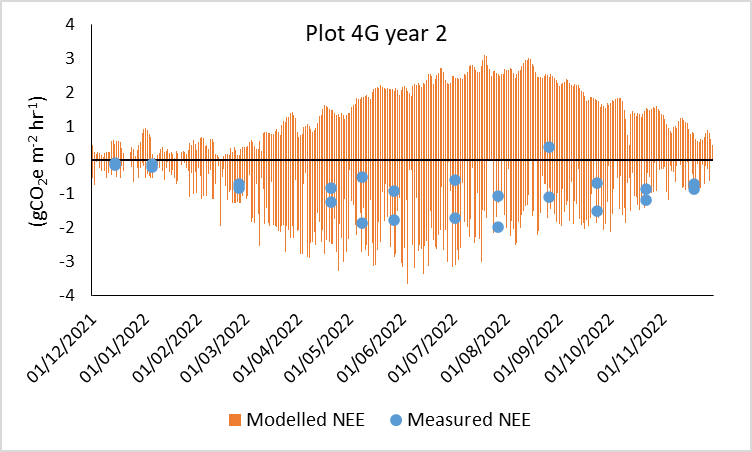 | 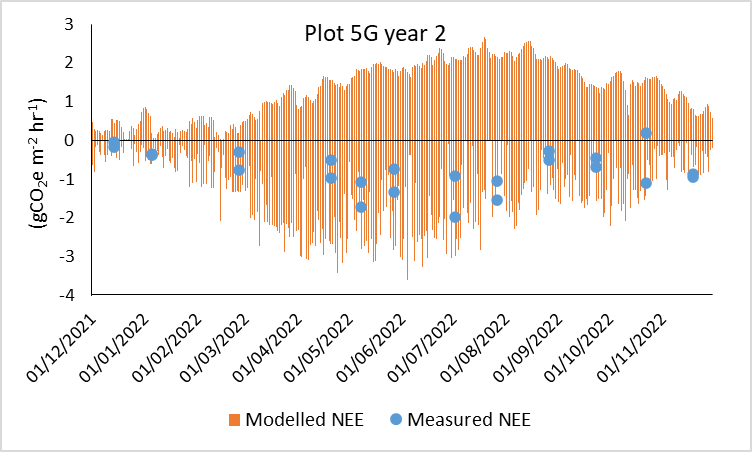 | 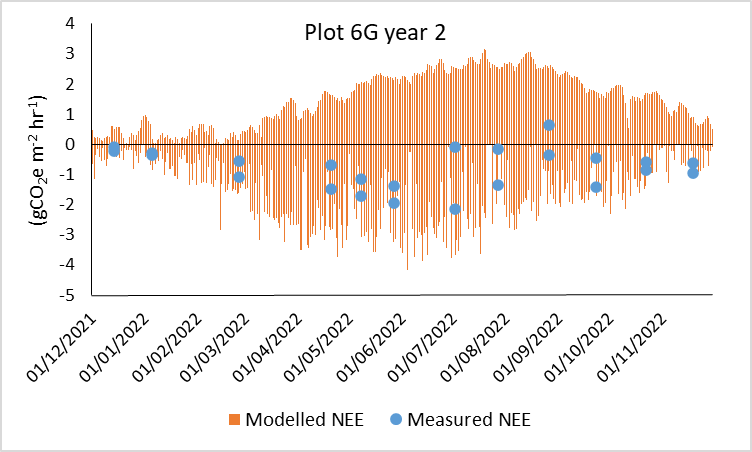 |

| 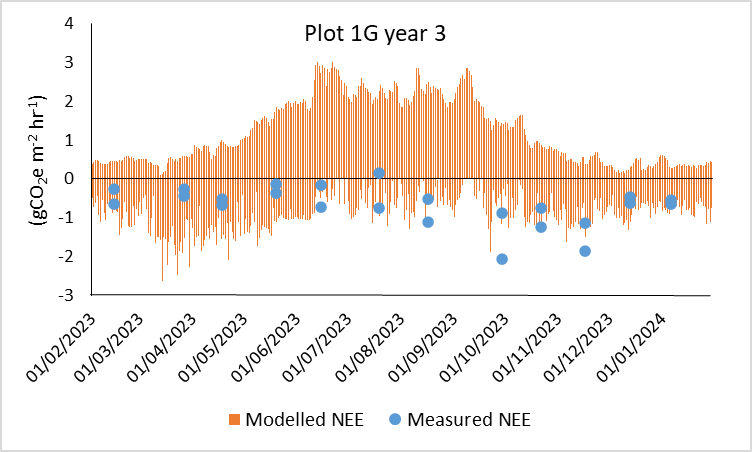 | 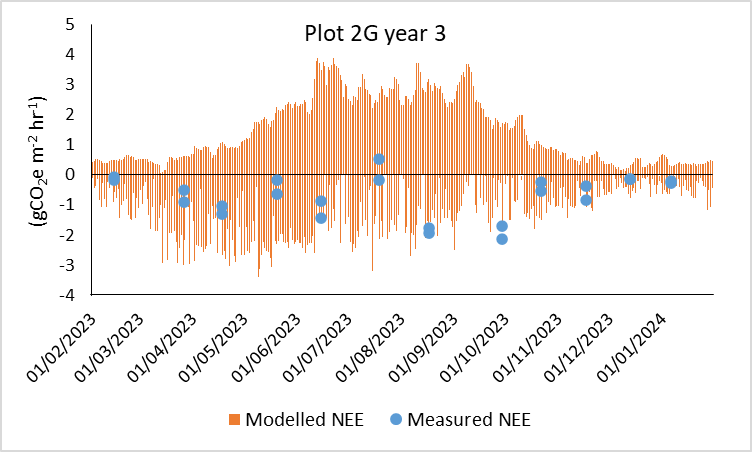 | 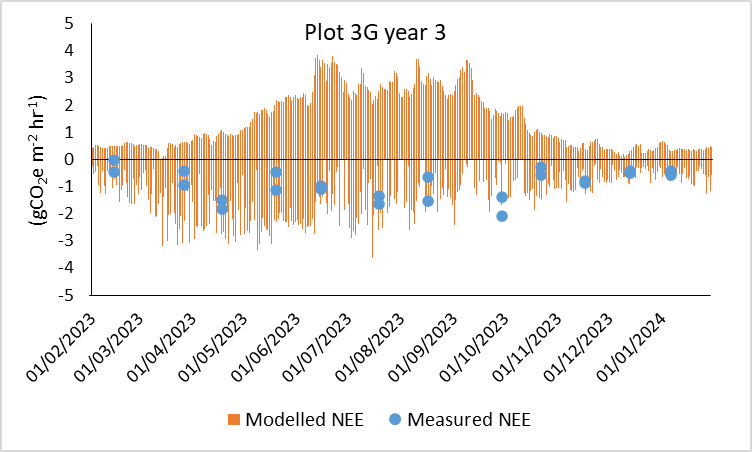 |
| --- | --- | --- |
| 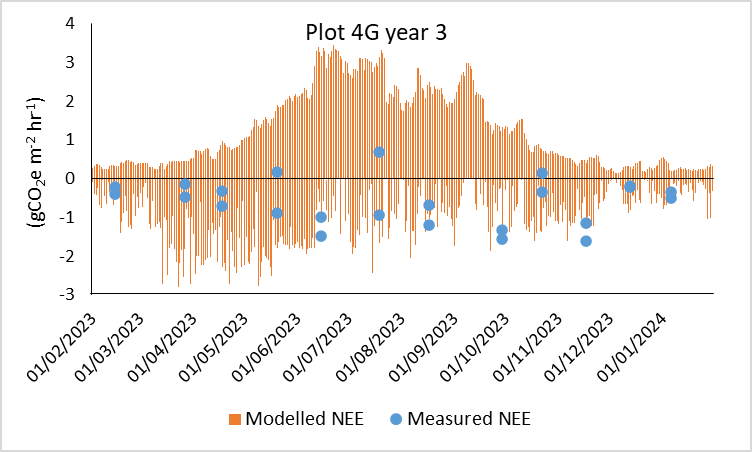 | 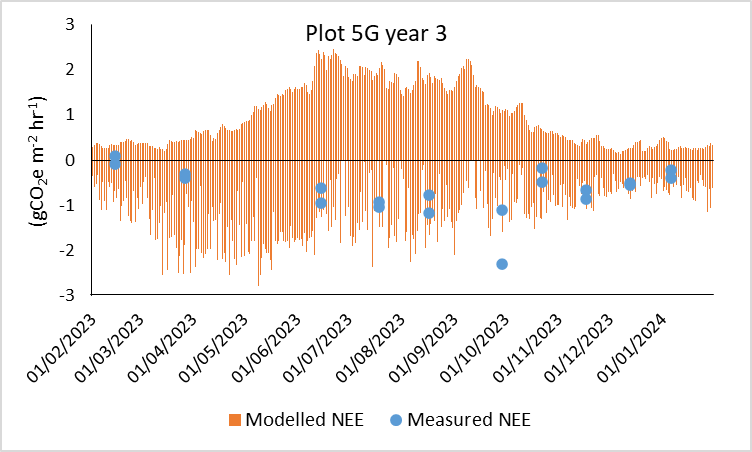 | 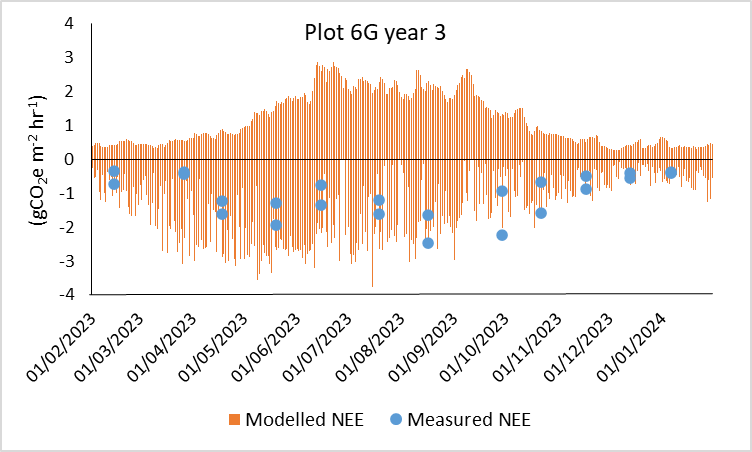 |

**Fig. S1** Graphs of Carbon GHG fluxes showing modelled and measured Net Ecosystem Exchange (NEE) CO_2_e fluxes on each treatment at both pilots for each year, both combined data from all plots and each plot individually; y-axes vary due to different start times each year but each graph represents a full year; Winmarleigh year 3 y-axis altered to start on 01/02/2023 for display purposes (actual start was 30/01/2023). Error bars on combined plot data graphs show measured standard deviation from the mean (*n* = 6). Modelled NEE includes night-time respiration/emission, hence graphs show both positive and negative values.

**Table S2** Statistical tests of difference between modelled and measured values of Emission and GPP (Emission and NEE on LWM Bare treatment) on each pilot site for each monitoring year, showing both combined and individual plot data; V/Veg. = Vegetated (Restoration), Bare = Bare (Control); CF/S = Carbon Farm (Restoration); GP/G = Grazed Pasture (Control); *n* = number of values; values are mean ± SD.
